# Structurally divergent dynamic combinatorial chemistry on racemic mixtures

**DOI:** 10.1038/s41467-020-17321-2

**Published:** 2020-07-15

**Authors:** Tiberiu-M. Gianga, G. Dan Pantoș

**Affiliations:** 1 Department of Chemistry, University of Bath, Bath, BA2 7AY UK; 2 Present Address: Beamline B23, Diamond Light Source, Ltd., Chilton, Didcot, OX11 0DE UK

**Keywords:** Origin of life, Organic chemistry, Dynamic combinatorial chemistry

## Abstract

Structurally Divergent Reactions on Racemic Mixtures are atypical processes in Nature. The few examples reported in the literature take place in organic solvents and are driven by the reagents’ interaction with bulky chiral catalysts. Herein, we describe a dynamic combinatorial approach to generate structural divergence from racemic building blocks. The divergence is due to a stereospecific electron-donor – electron-acceptor interaction of diastereomeric macrocycles, leading to structurally distinct pseudorotaxanes. The equilibrated dynamic combinatorial library contains, amongst various macrocycles, two different types of [2]catenanes that are non-isomeric. The formation of these [2]catenanes is due to a spontaneous stereo and structurally divergent assembly of the building blocks.

## Abstract (web summary)

Structurally divergent reactions on racemic mixtures, which produce distinct chemical species from an enantiomeric mixture, are extremely rare in the literature. Here, the authors are able to use a dynamic combinatorial approach to yield structurally divergent, non-isomeric [2]catenanes from an enantiomeric mixture.

## Introduction

Structurally divergent reactions on racemic mixtures (SDRRMs) utilise an enantiomeric mixture to produce two distinct chemical entities. This type of reaction is contrasting the predictable formation of diastereomers when two chiral entities react. SDRRM produces chemical species that are either structural isomers or non-isomeric. These species have different enthalpies of formation from starting materials that are isoenergetic. The energetic divergence observed in SDRRMs is due to each enantiomer following a different reaction pathway, and thus allowing for the formation of structurally different products. The energetic and structural divergence that occurs when these racemic mixtures react with a chiral molecule can provide insight into Nature’s chiral propagation question, as it explains how a small change at the molecular level leads to vastly different products.

To the best of our knowledge, only four SDRRMs^1^ have been reported in the literature^2–8^; of these, just two^3,4^ lead to non-isomeric structures. The structural divergence was usually achieved through the ability of one of the diastereomeric products to undergo a rearrangement or participate in a subsequent reaction with another compound present in the reaction mixture. The four known SDRRMs were developed in organic solvents and relied on structural constraints imposed by a bulky chiral catalyst.

We describe herein an aqueous SDRRM in a dynamic combinatorial environment to generate structural divergence from racemic building blocks. The reactions take place under the conditions of disulphide exchange in aqueous dynamic combinatorial libraries (DCL) and the resulting products are in stark contrast to our previous work, where enantiomers lead to the formation of diastereomers when reacted with a chiral building block^9,10^. In this dynamic combinatorial chemistry (DCC) system, the divergence is due to a combination of the assembly of diastereomeric macrocycles and a stereospecific electron-donor (*D*) – electron-acceptor (*A*) interaction, leading to structurally distinct pseudorotaxanes. These factors, combined with a difference in the stability of the final products, lead to the spontaneous assembly of two structurally different, non-isomeric [2]catenanes: one homochiral with a *DAAD* aromatic stack (**Cat I RRRR**), while the other is heterochiral with a *DADD* stack (**Cat II RSRR**). This work enhances our understanding of SDRRMs and raises the possibility of supramolecular interactions in aqueous media, playing a crucial role in biological homochirality^11–13^.

## Results

### Building block design

**Fig. 1:**
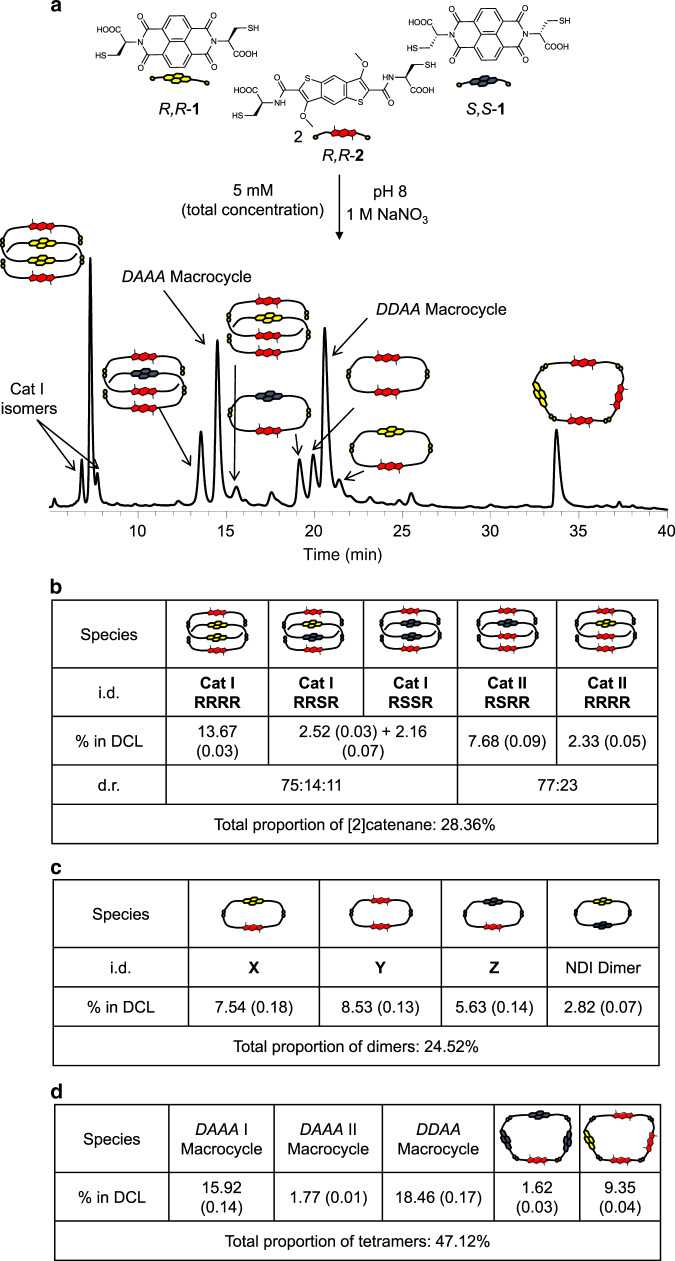
Racemic DCL. **a** Reverse-phase HPLC analysis at 389 nm of *R,R*-**1**:*S,S*-**1**:*R,R*-**2** (1:1:2 molar ratio, 5 mM total concentration) library in the presence of 1 M NaNO_3_; **b** the table shows the [2]catenanes i.d., percentages, diastereomeric ratios, and the total proportions of [2]catenanes in the DCL; **c** the table shows the dimers i.d., percentages and the total proportions of [2]catenanes in the DCL; **d** the table shows the macrocycles i.d., percentages and the total proportions of [2]catenanes in the DCL. The integrations were done in triplicate and the RMSD is reported in parenthesis along with the value. For a complete description of the library, see Supplementary Fig. 71.

The building blocks used throughout this study have cysteine appendages for disulphide exchange and water solubility, having either a naphthalenediimide (NDI, electron-acceptor) or benzodithiophene (BDT, electron-donor) cores. The enantiomeric NDIs (*R,R*-**1**, *S,S*-**1**) are readily available^14^, and have extensively been used in dynamic combinatorial chemistry^9,15–19^. The seven step synthesis of BDT, *R,R*-**2**, is reported here (see Supplementary Fig. 1). The BDT core is an electron-donor^20,21^ with a large negative quadrupole moment (−23 B), thus making it an ideal partner for aromatic stacking interactions with the electron-acceptor NDI, *Q*_zz_ = 14.2 B. A key feature in the structure of BDT is the presence of the two methoxy groups in positions 3 and 7, which form strong intramolecular H-bonds with the amide NH groups, thus further rigidifying the structure and making it a quasi-pentacyclic fused system. The additional rigidity makes the BDT building block more susceptible to the hydrophobic effect. The DCLs were set up by dissolving the building blocks in 10 mM aqueous NaOH to a total concentration of 5 mM. The pH was adjusted to 8 using a stock solution of 100 mM NaOH/100 mM HCl. The DCLs were stirred in the presence or absence of salt additives, and/or competitors (*vide infra*) for three days under air in capped vials to allow full oxidation of the building blocks. The DCL composed of 1.25 mM *R,R*-**1**, 1.25 mM *S,S*-**1** and 2.5 mM *R,R*-**2** contained, after the equilibrium was reached, a complex mixture of macrocycles and [2]catenanes. A detailed analysis revealed that the two enantiomers of **1** predominantly formed non-isomeric species. One of the most striking differences is the formation of the *DAAD* [2]catenane (**Cat I RRRR**), containing two NDI (*R,R*-**1**) and two BDT cores, while the *S,S*-**1** building block led to the formation of the *DADD* [2]catenane (**Cat II RSRR**), which contains one NDI (*S,S*-**1**) and three BDT cores (Fig. 1). These [2]catenanes are non isomeric despite one common (BDT) building block interacting with enantiomeric species (NDIs *R,R*-**1** and *S,S*-**1**). While the synthesis of [2]catenanes is in and of itself exciting, this work is set apart by the discovery of a different type of SDRRM driven by supramolecular interactions. This can open the door for the development of other divergent reactions on racemic mixtures based on aqueous, metal-free chemistry.

### Homochiral DCL

**Fig. 2:**
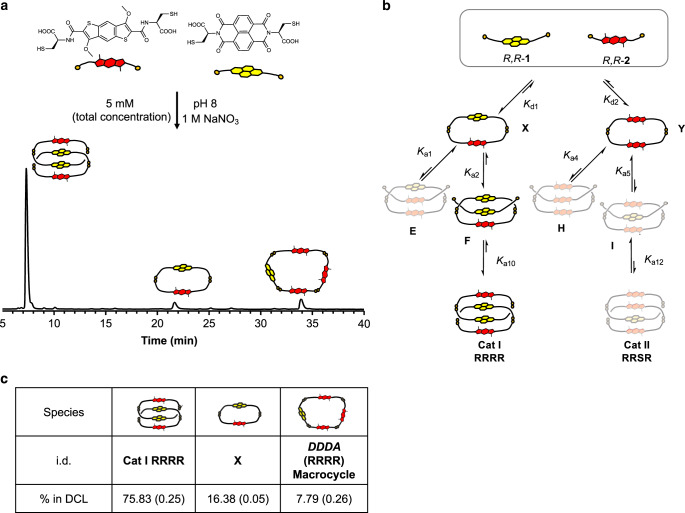
Homochiral DCL. **a** Reverse-phase HPLC analysis at 389 nm of *R,R*-**1**:*R,R*-**2** (1:1 molar ratio, 5 mM total concentration) library in the presence of 1 M NaNO_3_; **b** schematic representation of equilibria showing the pathway for the formation of **Cat I RRRR** and **c** table containing the yields for each species in DCL; the integrations were done in triplicate and the RMSD is reported in parenthesis along with the value.

To further interrogate this outcome, we set up DCLs containing *R,R*-**2** and each of the NDI enantiomers separately. The *R,R*-**1** (NDI) and *R,R*-**2** (BDT) library led, in the presence of 1 M NaNO_3_, to the assembly of the *DAAD* [2]catenane (**Cat I RRRR**) in 76% of the total library distribution (Fig. 2, for full characterisation see Supplementary Figs. 26–35). Sodium nitrate was added in order to increase the hydrophobic effect which leads to an increase in the yield of interlocked species (Supplementary Fig. 26)^16,18,22^.

### Heterochiral DCL

**Fig. 3:**
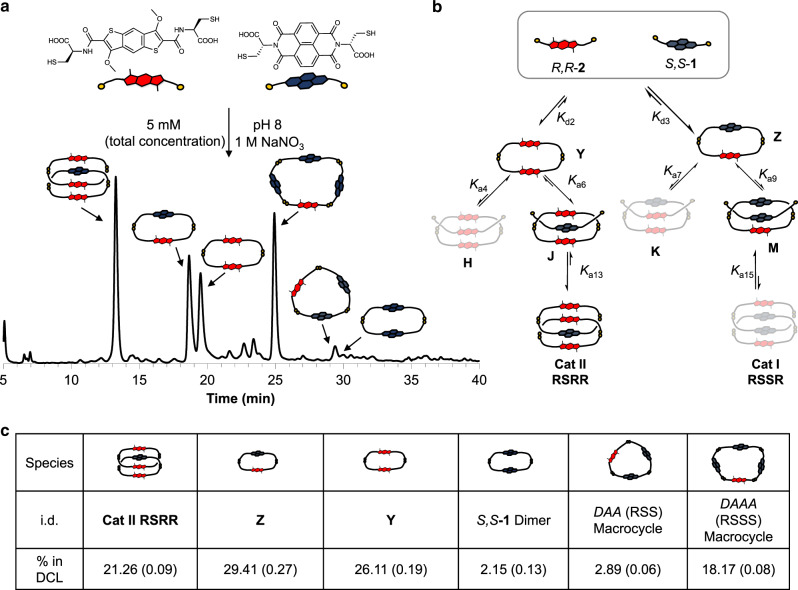
Heterochiral DCL. **a** Reverse-phase HPLC analysis at 389 nm of *S,S*-**1**:*R,R*-**2** (1:1 molar ratio, 5 mM total concentration) library in the presence of 1 M NaNO_3_; **b** schematic representation of equilibria showing the pathway for the formation of **Cat II RSRR** and **c** table containing the yield for each species in DCL; the integrations were done in triplicate and the RMSD is reported in parenthesis along with the value.

Contrasting the behaviour described above, the 1:1 *S,S*-**1**:*R,R*-**2** library led, in the presence of 1 M NaNO_3_, to the assembly of the *DADD* [2]catenane (**Cat II RSRR**) in 21% of the total library distribution (Fig. 3, for full characterisation see Supplementary Figs. 36–46). Changing the building block stoichiometry to 1:3 *S,S*-**1**:*R,R*-**2**, increased the proportion of **Cat II RSRR** up to 41%. The other major library members consisted of a donor dimer (**Y**), *DA* heterodimer (**Z**) and a *DAAA* (RSSS) macrocycle based on three *S,S*-**1** and one *R,R*-**2** molecules.

**Fig. 4:**
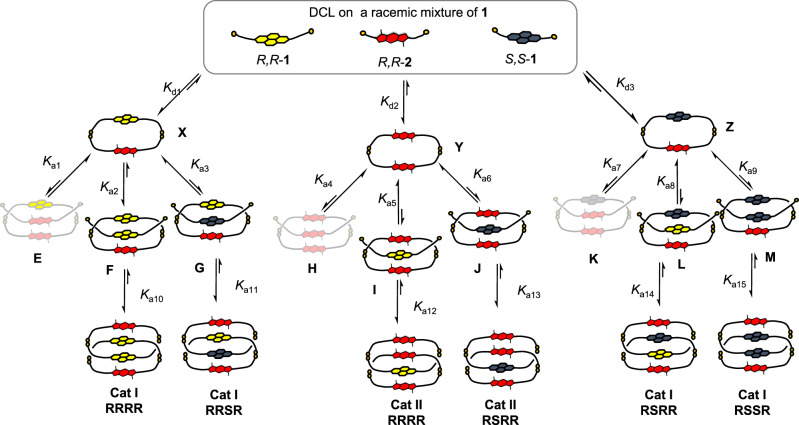
Rationalising the DCLs outcome. Schematic representation (simplified) of the pathways leading to **Cat I RRRR** and **Cat II RSRR**. The equilibrium arrows show the relative position of the equilibrium based on theoretical and experimental data. The faded structures are either present in less than 1% or not present in the DCLs analysed. *K*_d_ = dimerisation constant, *K*_a_ = association constant.

Once we characterised the outcomes of the racemic (Fig. 1) and enantiopure DCLs (Figs. 2, 3), we investigated in detail the behaviour of this interesting set of libraries along with other DCLs in which we varied the ratio of the donor and acceptor building blocks, the solvent polarity and composition (Supplementary Figs. 71, 74, 81). The DCL of a racemic mixture of **1** and *R,R*-**2** is an SDRRM because, with the exception of the *DAAD* catenane and the two *DA* heterodimers where we see some erosion of chirality, all of the other library members contain either only one of the enantiomers of **1** or a mixture of *R,R*-**1**, *S,S*-**1** and *R,R-***2**. All libraries have been studied at regular intervals until equilibration/full oxidation has been achieved (Supplementary Figs. 78–80). This allowed us to monitor and understand the evolution of all species involved in the assembly of **Cat I RRRR** and **Cat II RSRR**. Figure 4 depicts the major species and equilibria involved in their assembly (a full depiction of the possible library members—up to tetramers—is presented in Supplementary Fig. 91)^23,24^.

The kinetic study of the DCL evolution of *R,R*-**1** and *R,R*-**2** gives insight into the mechanism leading to the efficient assembly of **Cat I RRRR** (Supplementary Figs. 56–57). As expected, the first species formed in the DCL is the linear heterodimer, which is closely followed by the cyclic heterodimer (**X**, Fig. 2). Once a critical concentration of these two species is reached, **Cat I RRRR** starts to form, presumably by the threading of the linear heterodimer through the cyclic analogue, and subsequent final oxidative ring closing.

In contrast to this, the kinetic analysis of the DCL of *S,S*-**1** and *R,R*-**2** (Supplementary Figs. 67–68) reveals that the corresponding heterodimer **Z** (Fig. 3) forms in similar concentration as homodimer **Y** (a species not observed in the homochiral DCL, *vide supra*). **Cat II RSRR** is assembled through the complexation of an NDI core (*S,S*-**1**) in the cavity of **Y**, followed by ring closure. This process outcompetes the threading of the NDI core in the heterodimer **Z**, as indicated by the lack of the diastereomer of **Cat I RSSR** in this library.

The kinetic analysis of the DCL containing a racemic mixture of **1** and *R,R*-**2** reveals a very complex behaviour, in which all building blocks combine to form linear and cyclic species. The fastest forming species are the linear homochiral heterodimer **X** (20% of the library, based on HPLC peak integration after 30 min of equilibration), followed by its diastereomer, the heterochiral heterodimer **Z** (12%). This chiral differentiation is further amplified at the cyclisation stage, as after 85 min of DCL equilibration, the homochiral heterodimer **X** represents 13% of the DCL, while the heterochiral heterodimer **Z** amounts to only 4%. At this stage, the homodimer **Y** accounts for 8% of the DCL (Supplementary Figs. 79–80). Based on these observations, we propose that the structural divergence observed is due to two factors: (a) the preferential formation of two of the possible nine pseudorotaxanes (Fig. 4), and (b) the relative stability of the final reaction products, **Cat I RRRR** and **Cat II RSRR**.

The first species to form in all libraries are the linear and cyclic homodimers and heterodimers^25^. The cyclic homochiral heterodimer **X** forms in a larger proportion than the corresponding heterochiral heterodimer **Z** in the racemic libraries, which implies that the chirality mismatch in **Z** leads to less effective aromatic stacking interactions between the NDI and BDT cores. This is further supported by the fact that in the *S,S*-**1**, *R,R*-**2** library **Z** and the BDT homodimer **Y** are present in similar proportion despite that statistically, the ratio **Z**:**Y** should be 2:1 (the formation of **Cat II RSRR** does not influence the **Z**:**Y** ratio as it contains equal amount of both molecules in its composition).

The second key stage that contributes to the structural divergence is the formation of the pseudorotaxanes via the threading of a building block or linear dimer through the hetero or homodimers **X**, **Y** or **Z**. The homochiral heterodimer **X** will thread a NDI over a BDT core, accounting for better donor–acceptor stacking interactions (*K*_a2_, *K*_*a*3_ » *K*_a1_). Furthermore, the threading with a *R,R*-**1** is favoured over a *S,S*-**1** as indicated by the formation of **Cat I RRRR** via pseudorotaxane **F** over the corresponding diastereomer **G**. Control experiments, in which enantiopure serine analogues of **1** were used as competitors in DCLs containing *R,R*-**1** and *R,R*-**2**, confirmed this hypothesis as the NDI*-S,S*-serine lowers the yield of **Cat I RRRR** in comparison to NDI-*R,R*-serine (Supplementary Fig. 47), thus *K*_a2_ > *K*_a3_ » *K*_a1_ (NDI*-S,S*-serine is an analogue of *R,R*-**1**, as *S*-serine and *R*-cysteine are both l-amino acids). We used DCLSim^26^ to simulate the DCLs in the presence and absence of the NDI-serine competitors (**DCLSim** part in Supporting Information). The simulated DCLs distributions are in good agreement with the observed ones and confirmed that *K*_a2_ > *K*_a3_.

The BDT homodimer (**Y**) is the dominant species after 130 min in the DCL of *S,S*-**1**, *R,R*-**2** and, based on the stacking interactions, it likely generates the most stable pseudorotaxane (**J**) with *S,S*-**1** (Supplementary Figs. 67–68). In the racemic DCL, the competing species **I** does not form to a large extent, as confirmed by control DCLs with the enantiopure NDI-serine analogues and by the lack of the corresponding catenane (Supplementary Fig. 74). Additionally, UV–Vis titrations of **Y** with the DCC inert serine analogues^27^ highlighted a higher association constant between **Y** and NDI-*R,R*-serine compared to NDI-*S,S*-serine (Supplementary Figs. 86–87), therefore *K*_a6_ > *K*_a5_. This inequality is supported by DCLSim calculations, which also indicate that heterodimer **Z** binds *S,S*-**1** more strongly than *R,R*-**1**, with *K*_a9_ > *K*_a8_.

Pseudorotaxanes **E**, **K**, and **H** have unfavourable donor–donor stacking interactions^28,29^ due to the formation of an *ADD* stack (**E**, **K**) and a *DDD* stack (**H**) which allows us to conclude that relationship of the association constant is *K*_a1_, *K*_a7_ > *K*_a4_.

As in all DCLs, the stability of the final products plays a crucial role in the composition at equilibrium. **Cat I RRRR** and **Cat II RSRR** are more stable than their corresponding diastereomers as indicated by molecular modelling (PM7, COSMO water model, see “Methods” section). The **Cat I RRRR** is about 36.5 kJ/mol more stable than **Cat I RSSR**, and about 50 kJ/mol more stable than **Cat I RSRR**. There is a difference in stability for the **Cat II** diastereomers, where the **Cat II RSRR** is more stable than **Cat II RRRR** by almost 9 kJ/mol. In this case, the selectivity for **Cat II RSRR** in the racemic DCL is likely due to the incorporation of *R,R*-**1** in **Cat I RRRR** at the expense of **Cat II RRRR**. These differences are not surprising as the stacked structure of the catenanes forces the carboxylate groups to be in close proximity, therefore a change in the point chirality imposes new steric and electrostatic repulsion demands on the overall structure (Supplementary Table 6).

## Discussion

In conclusion, we have merged dynamic combinatorial chemistry with structurally divergent reactions on racemic mixtures. The structural divergence is due to the formation of two diastereomeric macrocycles and stereospecific formation of pseudorotaxanes under disulphide exchange conditions. This is an example of aqueous systems chemistry in which small changes in the building blocks’ structures lead to complex molecules through interconnected mechanisms. This SDRRM is driven by supramolecular interactions and highlights the importance of stereospecific supramolecular recognition observed in Nature. Here, we showed how a chiral reagent reacts with a racemic mixture to lead to compounds with non-isomeric structures based on which enantiomer is incorporated in the final product. This type of structural divergence based on supramolecular interactions could be the seed that led to the demise of one set of protobiomolecules resulting in the observed homochirality of the biological world. We are currently investigating the formation of other conformationally restricted molecules from racemic mixtures.

## Methods

### Materials and characterisation

All reagents were purchased from commercial suppliers: Acros Organics, Alfa Aesar, Sigma Aldrich, TCI Europe, Fluorochem, and used without further purification. ^1^H and ^13^C nuclear magnetic resonance (NMR) spectra were recorded on 500 MHz Agilent Propulse or 500 MHz Bruker Avance II+ (^1^H 500 MHz, ^13^C 125 MHz) instruments, as stated. Chemical shifts (*δ*) are reported in parts per million (ppm). Coupling constants are reported in Hertz (Hz), and signal multiplicity is denoted as singlet (s), doublet (d), doublet of doublet (dd), triplet (t), dt (doublet of triplets), td (triplet of doublets), h (heptet), multiplet (m), and broad (br). All spectra were acquired at 25 °C and were referenced to the residual solvent peaks. The common solvent impurities in ^1^H and ^13^C NMR in small amounts were water, acetone or DMF. COSY, NOESY, and HSQC spectra were recorded on a Bruker AV III 500 MHz fitted with Prodigy (nitrogen-cooled) cryoprobe. The microwave reactions were carried out in either CEM Discover or CEM Explorer 12. LC-MS studies were carried out on a Thermo Surveyor PDA Plus LC and LCQ classic ESI MS. Data was processed using the XCalibur software. The individual HPLC/LC-MS methods are detailed in the LC-MS Analysis Section. The LC CD data was acquired on a Jasco CD 2095 connected to a SS420× A/D converter. All the CD data was acquired on an Applied Photophysics Chirascan spectro-photometer equipped with a Peltier temperature controller.

### DCL set-up

A 5 mM library was prepared by dissolving the building block in 10 mM aqueous NaOH, followed by titration with 100 mM NaOH/100 mM HCl to adjust the pH to 8. The DCL solutions were stirred in close-capped vials and analysed by LC-MS. Preparative libraries (≥ 5 mL scale) were made using the same method as for the analytical libraries.

### LC-MS method

ESI-MS spectra (negative ion) were acquired with drying temperature of 250 °C, spray current 0.5 μA, sheath gas flow of 40 arb, spray voltage was set to 4.5 kV, capillary voltage 13 V, tube lens −15.0 V. The mass range was set from *m/z* 150–2000, the number of microscans in scan time was 5 and the maximum injection time was 150.0 ms.

### MS/MS setting

Parent mass (*m/z*): dependant on the species, ionisation width (*m/z*): 4.0, normalised collision energy (%): 20, activation Q: 0.250, activation time (ms) 30.0, the number of microscans in scan time was 5 and the maximum injection time was 150.0 ms.

### Analytical HPLC method for DCLs of Cat I RRRR (5 mM)

Column**:** Phenomenex Develosil RPAqueous-3 C_30_, 5 × 0.3 cm, 3 μm. Injection volume: 2 μL; flow rate: 1 mL/min; temperature: 39 °C; run time: 7 min; elution profile: See Supplementary Table 1.

### Preparative HPLC method for DCLs of Cat I RRRR (5 mM)

Column**:** Phenomenex Develosil Combi-RP C_30_, 5 × 2 cm, 5 μm; injection volume: 900 μL; Flow rate: 6 mL/min; temperature: 39 °C; Run time: 34 min; elution profile: see Supplementary Table 2.

### Analytical HPLC method for DCLs of Cat II RSRR (5 mM)

Column**:** Phenomenex Develosil RPAqueous-3 C_30_, 5 × 0.3 cm, 3 μm. Injection volume: 2 μL; flow rate: 1 mL/min; temperature: 39 °C; run time: 7 min; elution profile: see Supplementary Table 3.

### Preparative HPLC method for DCLs of Cat II RSRR (5 mM)

Column**:** Phenomenex Develosil Combi-RP C_30_, 5 × 2 cm, 5 μm; injection volume: 600 μL; flow rate: 6 mL/min; temperature: 39 °C; run time: 24 min; elution profile: See Supplementary Table 4.

### Analytical HPLC method for DCLs of all libraries (5 mM)

Column**:** Restek Ultra BiPhenyl, 5 × 0.3 cm, 3 μm. Injection volume: 3 μL; flow rate: 0.5 mL/min; temperature: 39 °C; run time: 43 min; elution profile: See Supplementary Table 5.

### Circular dichroism settings for isolated Cat I RRRR and Cat II RSRR analysis

The isolated catenane (after purity checking by HPLC), **Cat I RRRR** or **Cat II RSRR**, was assessed for optical and chiroptical properties. The catenane has a CD band across the 550 nm region, which is expected for *D*–*A* interaction. The experiments were done in a 1 or 10 mm pathlength cuvettes depending on the concentration, with a 4 nm bandwidth. Scan mode: 1 point/nm, time-per-point 0.5 s. The VT experiments (10 °C increment and setting time 300 s) were performed in the same cuvette using a stirrer bar.

### UV–Vis titration of NDI-*R,R*/*S,S*-serine into Y

**Y** was prepared by dissolving the *R,R*-**2** building block in water at pH 8 and allowed to equilibrate over two days, yielding **Y** homodimer as the major product (more than 90%). From this stock solution, two solutions (3.57 mM) were prepared: one containing the homodimer and 1 M NaNO_3_ (soln. T) and the other with NDI-*R,R*-serine or NDI-*S,S*-serine (35.7 mM, 10 equiv) and 1 M NaNO_3_ (soln. U). Small aliquots of soln. U were gradually added into soln. T, having 0.1, 0.3, 0.5, 0.7, 0.8, 0.9, 1.0, 1.1, 1.2, 1.5, 2.0, 3.0, 4.0, and 5.0 equiv of NDI-serine at each titration point in the final solution.

### Testing reversibility with DTT

In order to test the reversible behaviour of the reactions, both **Cat I RRRR** and **Cat II RSRR** were subjected to dithiothreitol (DTT) after isolation. DTT is a dithiol-based compound that can restart the disulphide exchange and reform the library.

### Computational studies

Geometry optimisation was performed using Avogadro^30^ (Force field: UFF, Algorithm: Conjugate gradients). This was followed by MOPAC 2016^31^ (Version 18.117 M) optimisation PM7 semi-empirical optimisation using the COSMO water model with a 0.1 kcal/mol/Ångstrom convergence factor, using Gabedit^32^ 2.5.0 as interface.

## Supplementary information

Supplementary Information

## Data Availability

The data that support the findings of this study are available within the paper and its Supplementary Information with 10.1038/s41467-020-17321-2 All data underlying the findings of this work are available from the corresponding author upon reasonable request.
