# Supplementary material for: Structurally divergent dynamic combinatorial chemistry on racemic mixtures
Source: Nat Commun. 2020 Jul 15;11:3528. doi: 10.1038/s41467-020-17321-2 (PMC7363883; doi:10.1038/s41467-020-17321-2)
Supplement: Supplementary file 1 — Supplementary Information [file 41467_2020_17321_MOESM1_ESM.pdf]

Supplementary Information

Structurally Divergent Dynamic Combinatorial Chemistry on Racemic Mixtures

Gianga *et al.*

Table of Contents

Supplementary Methods *3*

Synthesis of building blocks 3

NMR Spectra 9

HPLC methods for analyzing the DCLs: 19

Supplementary Note 1 - Methods for the integration of the HPLC peaks *20*

Analysis of Cat I RRRR 23

Analysis of Cat II RSRR 31

Analysis of DCLs containing R,R-1 and R,R-2 41

Analysis of DCLs containing S,S-1 and R,R-2 54

Analysis of DCLs containing R,R-1, S,S-1 and R,R-2 65

UV-Vis / CD spectra of building blocks 73

UV-Vis titration of NDI-R,R/S,S-serine into Y 74

Testing reversibility with DTT 76

Computational studies 77

DCLSim 78

Supplementary References *86*

Supplementary Methods

Synthesis of building blocks

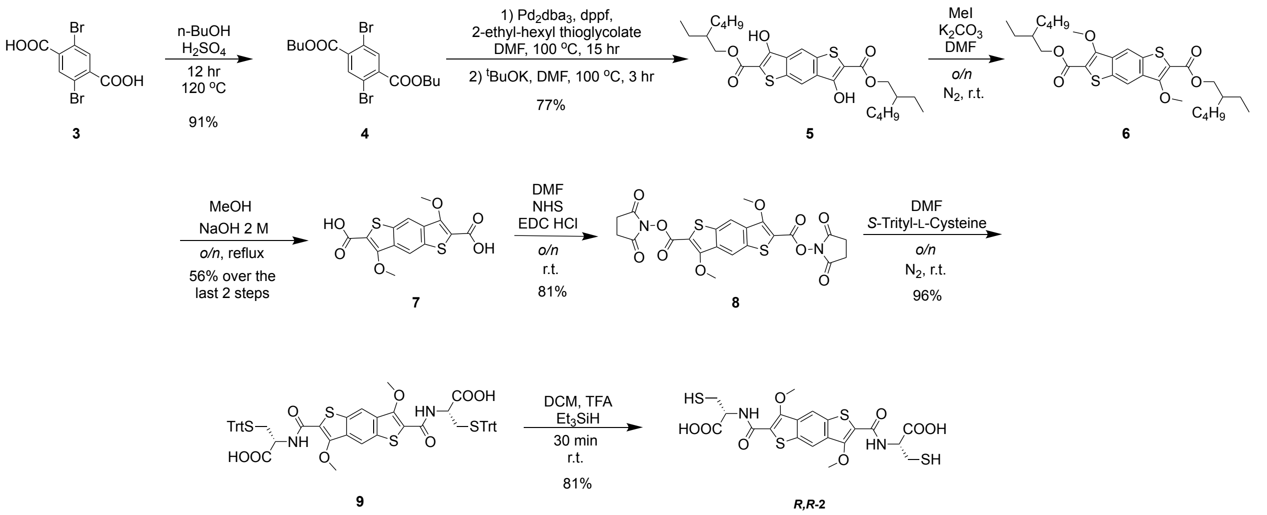

Supplementary Figure 1. The synthetic scheme of *R,R*-**2**.

Compound **4** was synthesized following the published procedures: 2,4-dibromoterephthalic acid and *n*-BuOH (4.7 mL for each mmol of **3**) was added in a round bottom flask, followed by addition of H_2SO_4 (0.07 mL for each mmol of **3**) and heated at 120 °C overnight. After evaporating most of *n*-BuOH (about 10 mL left) and neutralisation with aqueous $NaHCO_3$, the product was extracted with ethyl acetate and the solution was dried over anhydrous $MgSO_4$. After the full removal of the solvent, the residue was purified using column chromatography (petroleum ether : ethyl acetate 98:2) to give the product as a white solid. The ^{1}H NMR spectrum matches the one reported in literature.[1]

Compound **5** was synthesised using a modified procedure:

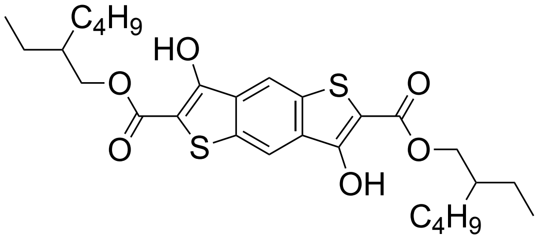

4 (5.51 g, 12.70 mmoles, 1 equiv), $Pd_2(dba)_3$ (0.578 g, 0.63 mmoles, 0.05 equiv) and dppf (0.70 g, 1.26 mmoles, 0.1 equiv) were dissolved in DMF (70 mL) under N_2. To this mixture, $^iPr_2NEt$ (11 mL, 63.14 mmoles, 4.97 equiv) and 2-ethylhexyl thioglycolate (6.34 mL, 30.16 mmoles, 2.37 equiv) were added and the resulting solution was heated at 100 °C for 15 h. After cooling to r.t., ^tBuOK (4.10 g, 36.53 mmoles, 2.87 equiv) and DMF (40 mL) were added and the solution reheated to 100 °C for further 3 h. After cooling to r.t., 1 M HCl was added to acidify the solution, followed by extraction with toluene, filtration over Celite® and washings with ethyl acetate. After removing the solvent, the compound was purified by column chromatography (CH_2Cl_2 : Petroleum Ether 60:40 *v*/*v*), giving compound **5** (5.19 g, 9.71 mmoles, 77%). The ^{1}H NMR spectrum is matching the one reported in literature.[1]

Synthesis of **6**:

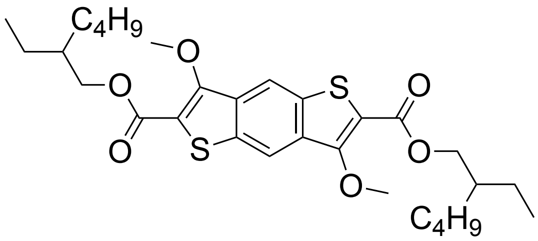

In a round-bottom flask, **5** (3.00 g, 5.60 mmoles,1 equiv) was dissolved in DMF (200 mL) under N_2 atmosphere followed by addition of K_2CO_3 (3.88 g, 28.68 mmoles, 5 equiv). After stirring half an hour at r.t., MeI (1.4 mL, 22.48 mmoles, 4 equiv) was added and further stirred overnight. Water was added, and the compound was extracted with CH_2Cl_2. The organic phase was dried over $MgSO_4$ anhydrous and the solvent removed *in vacuo* to yield an oily liquid, which was dried under high pressure. At this stage the compound was impure but used as it was; no yield was determined at this stage. ^{1}H NMR (500 MHz, $CDCl_3$): δ 8.26 (s, 2H), 4.27 (dd, *J* = 5.7, 3.6 Hz, 4H), 4.20 (s, 6H), 1.73 (h, *J* = 5.9 Hz, 4H). The rest of the protons cannot be assigned because of the complexity due to impurities. ^{13}C NMR (125 MHz): δ 169.6, 162.5, 161.6, 155.5, 140.0, 134.5, 134.3, 122.7, 117.1(3), 117.0(8), 117.0(2), 68.0, 67.8, 62.8, 38.9, 38.9, 36.4, 31.4, 30.5(1), 30.4(7), 29.0, 28.9, 23.9(3), 23.9(0). There are more peaks in the ^{13}C NMR spectrum than expected because of the impurity. TOF MS ASAP+: *m/z* calcd for $C_{30}H_{42}O_6S_2$ $[M+H]^+$ 563.2501, found 563.2504.

Synthesis of **7**:

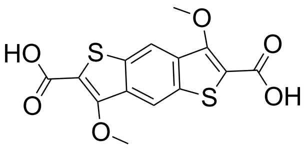

In a round-bottom flask, **6** (3.95 g) was dissolved in acetone (5 mL), followed by addition of MeOH (500 mL) and 2 M NaOH (250 mL); the reaction was stirred overnight at reflux. Upon cooling, concentrated HCl was added until the pH became acidic and a yellow solid precipitated. The precipitate was filtered and washed with CH_2Cl_2 and acetone. The precipitate was collected and dried to yield **7** as a yellow solid (1.05 g, 3.12 mmoles, 58% over 2 steps). ^{1}H NMR (500 MHz, DMSO-d_6): δ 13.46 (br, 2H), 8.50 (s, 2H), 4.09 (s, 6H). ^{13}C NMR (125 MHz): δ 162.9, 154.8, 134.5, 134.0, 118.8, 117.9, 63.0. TOF MS ASAP+: *m/z* calcd for $C_{14}H_{10}O_6S_2$ $[M+H]^+$ 338.9997, found 338.9996.

Synthesis of **8**:

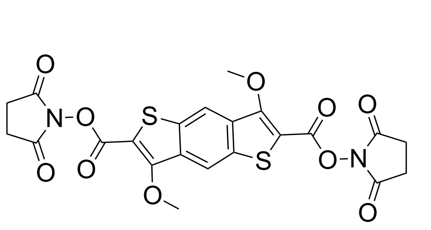

Compound **7** (1.05 g, 3.10 mmoles, 1 equiv) and *N*-hydroxysuccinimide (1.44 g, 12.50 mmoles, 4 equiv) were dissolved in DMF (110 mL) and cooled to 0 °C. EDC·HCl (2.41 g, 12.50 mmoles, 4 equiv) was added and the reaction was stirred for 15 minutes in the melting ice bath. The reaction was further stirred overnight at r.t. The solvent was

removed under reduced pressure and a small amount of acetone was added. The suspension formed was added dropwise to a 1 M HCl solution vigorously stirred. The precipitate obtained was collected by vacuum filtration and dried under reduce pressure to obtain a brownish precipitate (1.40 g, 2.60 mmoles, 85%). ^{1}H NMR (500 MHz, DMSO-*d*$_6$): δ 8.86 (s, 2H), 4.21 (s, 6H), 2.89 (s, 8H). ^{13}C NMR (125 MHz): δ 170.6, 159.4, 157.3, 135.4, 134.1, 119.7, 110.6, 63.7, 26.0. FTMS+pNSI: *m/z* calcd for $C_{22}H_{16}N_2O_{10}S_2$ $[M+NH_4]^+$ 550.0585, found 550.0580.

Synthesis of **9**:

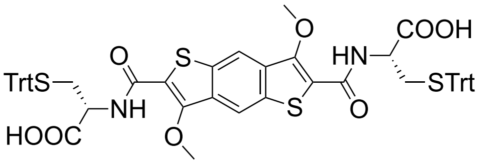

In a flame-dried round-bottom flask, *S*-trityl-L-cysteine (612.2 mg, 1.70 mmoles, 2.2 equiv) was added to a solution of **8** (401.8 mg, 0.76 mmoles, 1 equiv) in DMF (40 mL) under N_2. Et_3N (1 mL) was added and the reaction was stirred overnight at r.t. The solvent was removed under pressure and a small volume of acetone was added to the mixture, which was precipitated dropwise into a 1 M HCl solution vigorously stirred. The yellow precipitate was collected by filtration and dried (727.8 mg, 0.76 mmoles, quant.). ^{1}H NMR (500 MHz, DMSO-*d*$_6$): δ 13.19 (br, 2H), 8.70 (s, 2H), 8.42 (d, J = 7.6 Hz, 2H), 7.35-7.15 (m, 30H), 4.55 (td, *J* = 7.3, 4.6 Hz, 2H) 4.16 (s, 6H), 2.79 (dd, *J* = 12.3, 7.0 Hz, 2H). The other beta proton of cysteine moiety is under the satellite of the deuterated solvent. ^{13}C NMR (125 MHz): δ 176.3, 165.4, 155.1, 149.3, 138.7, 137.2, 134.3, 133.2, 132.1, 71.3, 67.7, 56.5. FTMS-pNSI: *m/z* calcd for $C_{58}H_{48}N_2O_8S_4$ $[M-H]^-$ 1027.2221, found 1027.2198.

Synthesis of *R,R*-**2**:

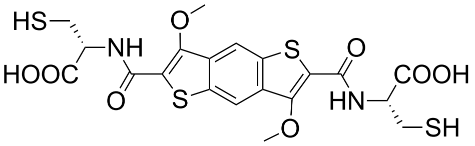

In a round-bottom flask, **9** (500.4 mg, 0.49 mmoles) was dissolved in CH_2Cl_2 (10 mL) followed by addition of TFA (10 mL) and $SiEt_3H$ (1 mL). The reaction mixture was stirred 30 minutes at r.t. and the volatiles were subsequently removed. Et_2O was added, removed under pressure and re-dissolved in Et_2O to ensure the precipitation of the desired product. To this, n-hexane was added and the solid was filtered and further washed with *n*-hexane to yield *R,R*-**2** as an yellow precipitate that was dried (228.2 mg, 0.42 mmoles, 86%). ^{1}H NMR (500 MHz, DMSO-*d*$_6$): δ 13.29 (br, 2H), 8.69 (s, 2H), 8.47 (d, *J* = 7.4 Hz, 2H), 4.76 (dt, *J* = 7.4, 4.9, 2H) 4.20 (s, 6H). 3.15-3.00 (m, 4H). ^{13}C NMR (125 MHz): δ 171.6, 160.8, 150.5, 134.0, 132.6, 124.4, 118.2, 63.0, 54.3, 26.2. FTMS-pNSI: *m/z* calcd for $C_{20}H_{19}N_2O_8S_4$ $[M-H]^-$ 543.0030, found 543.0032.

NDI-Trt-*R*-Cys and **NDI-Trt-*S*-Cys** were synthesised following the previously published procedures: NDA (1 equiv) and *S*-trityl-L-cysteine (for **NDI-Trt-*R*-Cys**) (2.1 equiv) (or *S*-trityl-D-cysteine in the case of **NDI-Trt-*S*-Cys**) were dissolved in DMF (for <300 mg of NDA use 5 mL, otherwise use 10 mL) in a 20 mL microwave tube (maximum amount of NDA is 1 g). Dry triethylamine (1 mL) was added and the mixture was sonicated until a

clear solution was formed. The solution was heated in a microwave reactor for 5 min at 120 °C. When the reaction was finished, the solvent was removed under reduced pressure. The residue was redissolved in acetone (1-2 mL) and the solution was added dropwise to a vigourously stirred 1 M HCl aqueous solution. The suspension was filltered and the precipitate formed was dried under high pressure. The ^{1}H NMR spectra matched the ones reported in literature.[2]

Synthesis of *S*,*S*-**1**:

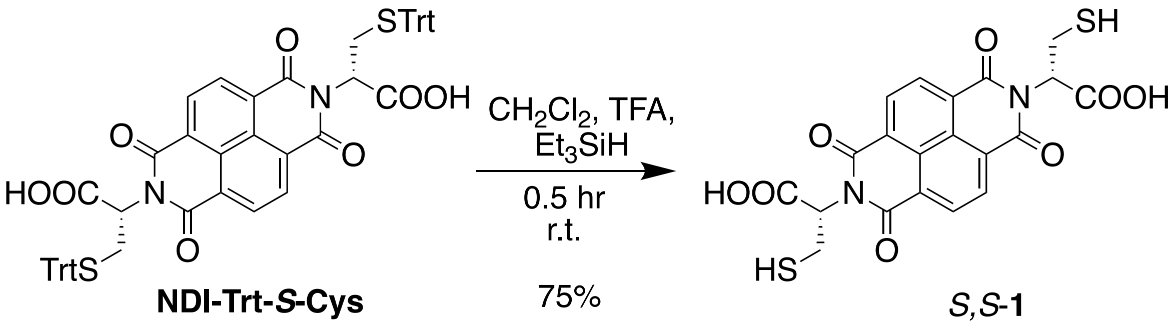

Supplementary Figure 2. Synthesis scheme of *S,S*-**1**.

NDI-Trt-*S*-Cys (201.0 mg, 0.21 mmoles, 1 equiv) was dissolved in a mixture of CH_2Cl_2 (3 mL) and TFA (3 mL), followed by the addition of Et_3SiH (0.3 mL). The reaction mixture was stirred for half an hour at r.t. and the volatiles were removed under reduced pressure. Et_2O was added the suspension followed by the addition of *n*-hexane. The precipitate formed was filtered and dried (74.9 mg, 0.16 mmoles, 75%). ^{1}H NMR (500 MHz, DMSO-*d6*) δ 13.22 (s, 2H), 8.78 (s, 4H), 5.73 (dd, *J* = 9.3, 5,3 Hz, 2H), 3.22 (dt, *J* = 14.2, 9.3 Hz, 2H), 2.72 (t, *J* = 8.9 Hz, 2H). The other two beta protons are under the water peak. ^{13}C NMR (125 MHz) δ 170.0, 162.9, 131.8, 126.9, 126.5, 56.5, 23.2. TOF MS ASAP+: *m/z* calcd for $C_{20}H_{14}N_2O_8S_2$ $[M+H]^+$ 475.0270, found 475.0273.

R,R-**1** was synthesised using the same procedure as *S,S*-**1**.

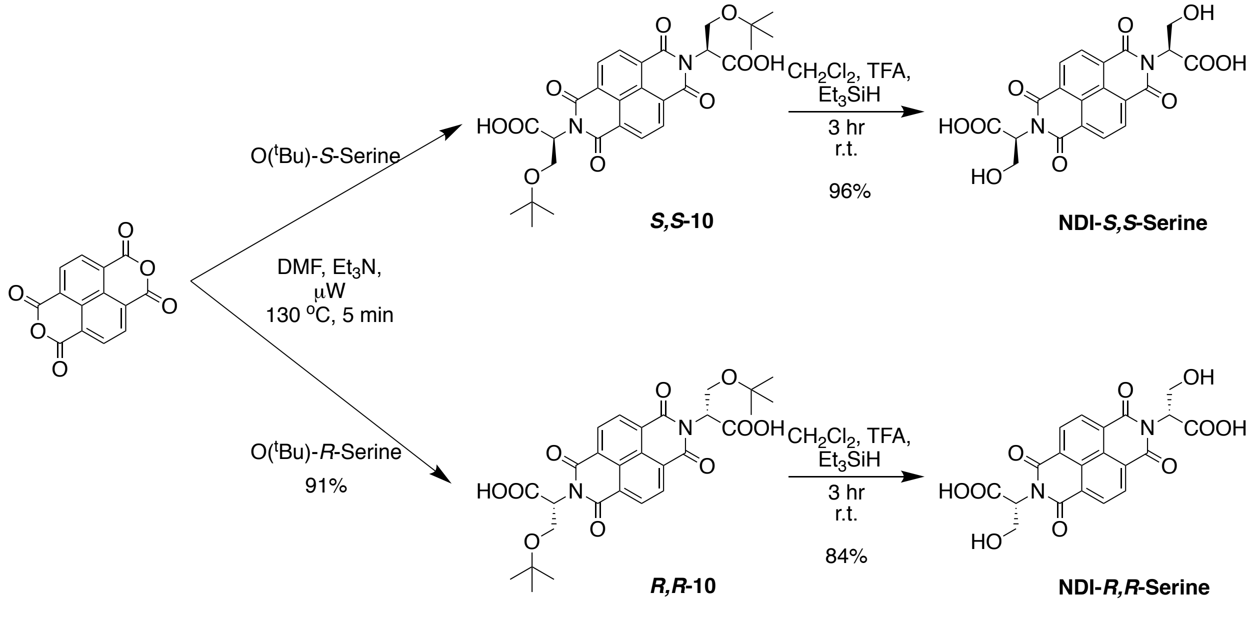

Supplementary Figure 3. Synthesis scheme of **NDI-*S,S*-Serine** and **NDI-*R,R*-Serine**.

S,S-**10** and *R,R*-**10** were synthesised using the published protocols and the same procedure as for **NDI-Trt-*R*-Cys** and **NDI-Trt-*S*-Cys**.

Synthesis of *S,S*-**10**:

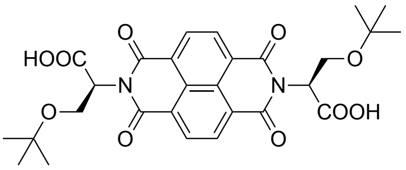

S,S-**10**: Started from NDA (202.6 mg, 0.76 mmoles, 1 equiv) and *O*-^{t}Bu-L-Serine (253.8 mg, 1.57 mmoles, 2.1 equiv). Obtained: (378.1 mg, 0.68 mmoles, 91%) *S,S*-**10**. ^{1}H NMR (500 MHz, $CDCl_3$) δ 8.80 (s, 4H), 5.74 (t, *J* = 6.3 Hz, 2H), 4.43 (dd, *J* = 11.5, 6.3 Hz, 2H), 4.04 (dd, *J* = 11.6, 6.3 Hz, 2H), 1.46 (s, 18H). ^{13}C NMR (125 MHz) δ 167.6, 162.6, 131.4, 126.5, 83.2, 60.9, 55.7, 27.9. FTMS-pNSI: *m/z* calcd for $C_{28}H_{30}N_2O_{10}$: 553.1828 [*M*-H]$^-$; found 553.1829.

Synthesis of *R,R*-**10**:

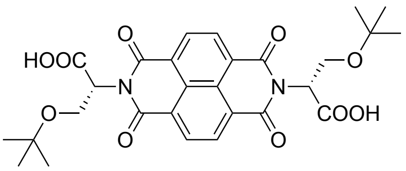

R,R-**10**: Started from NDA (200.7 mg, 0.75 mmoles, 1 equiv) and *O*-^{t}Bu-D-Serine (253.8 mg, 1.57 mmoles, 2.1 equiv). Obtained: (378.1 mg, 0.68 mmoles, 91%) *R,R*-**10**. ^{1}H NMR (500 MHz, $CDCl_3$) δ 8.77 (s, 4H), 5.93 (t, *J* = 7.7 Hz, 2H), 4.35 (t, *J* = 8.6 Hz, 2H), 3.92 (dd, *J* = 9.1, 7.3 Hz, 2H), 1.24 (s, 18H). ^{13}C NMR (125 MHz) δ 162.3, 131.5, 126.4, 58.9, 52.9, 27.3. FTMS-pNSI: *m/z* calcd for $C_{28}H_{30}N_2O_{10}$: 553.1828 [*M*-H]$^-$; found 553.1828.

Deprotection procedure:

The protected species was dissolved in a mixture of CH_2Cl_2 (3 mL) and TFA (3 mL), followed by the addition of Et_3SiH (0.3 mL). The reaction mixture was stirred for 3 hours at r.t. and the volatiles were removed under reduced pressure. Et_2O was added to the residue and the solid formed was filtered and dried.

Synthesis of **NDI-*S,S*-serine**:

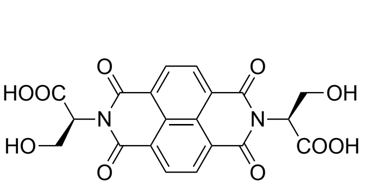

NDI-*S,S*-serine: Started from *S,S*-**10** (200.4 mg, 0.36 mmoles, 1 equiv) and formed **NDI-*S,S*-Serine** (152.6 mg, 0.35 mmoles, 96%). ^{1}H NMR (500 MHz, DMSO-d_6) δ 13.01 (br s, 2H), 8.75 (s, 4H) 5.71 (t, *J* = 7.2 Hz, 2H), 4.90 (s, 2H), 4.09 (d, *J* = 7.2 Hz, 4H). ^{13}C NMR (125 MHz) δ 169.3, 162.5, 131.1, 126.1, 58.2, 55.6. FTMS-pNSI: *m/z* calcd for $C_{20}H_{14}N_2O_{10}$: 441.0576 [*M*-H]$^-$; found 445.0576.

Synthesis of **NDI-*R,R*-serine**:

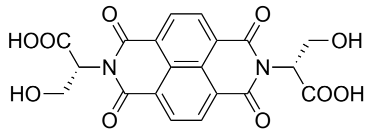

NDI-*R,R*-serine: Started from *R,R*-**10** (199.7 mg, 0.36 mmoles, 1 equiv) and formed **NDI-*R,R*-Serine** (133.3 mg, 0.30 mmoles, 84%). ^{1}H NMR (500 MHz, DMSO-d_6) δ 13.01 (br s, 2H), 8.74 (s, 4H), 5.70 (t, J = 7.2 Hz, 2H), 4.83 (s, 2H*),* 3.98 (t, J = 7.2 Hz, 4H). ^{13}C NMR (125 MHz) δ 169.7, 162.9, 131.6, 126.6, 58.7, 56.0. FTMS-pNSI: *m/z* calcd for $C_{20}H_{14}N_2O_{10}$: 441.0576 [*M*-H]$^-$; found 445.0575.

NMR Spectra

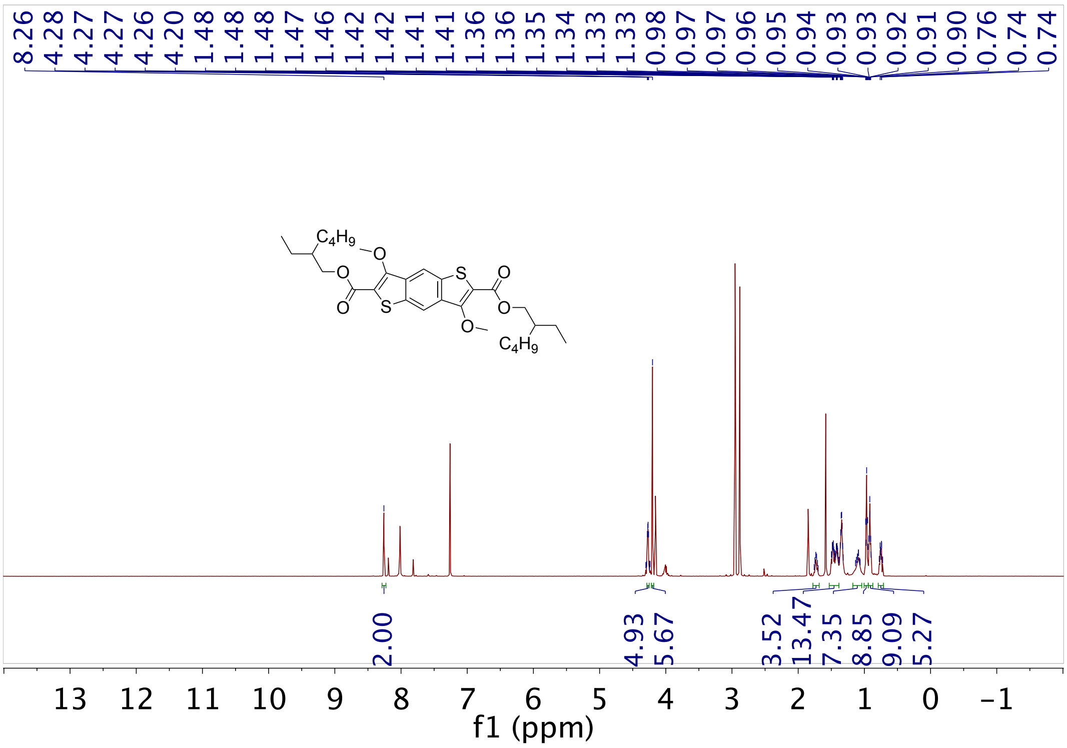

Supplementary Figure 4. ^{1}H NMR of **6** in $CDCl_3$.

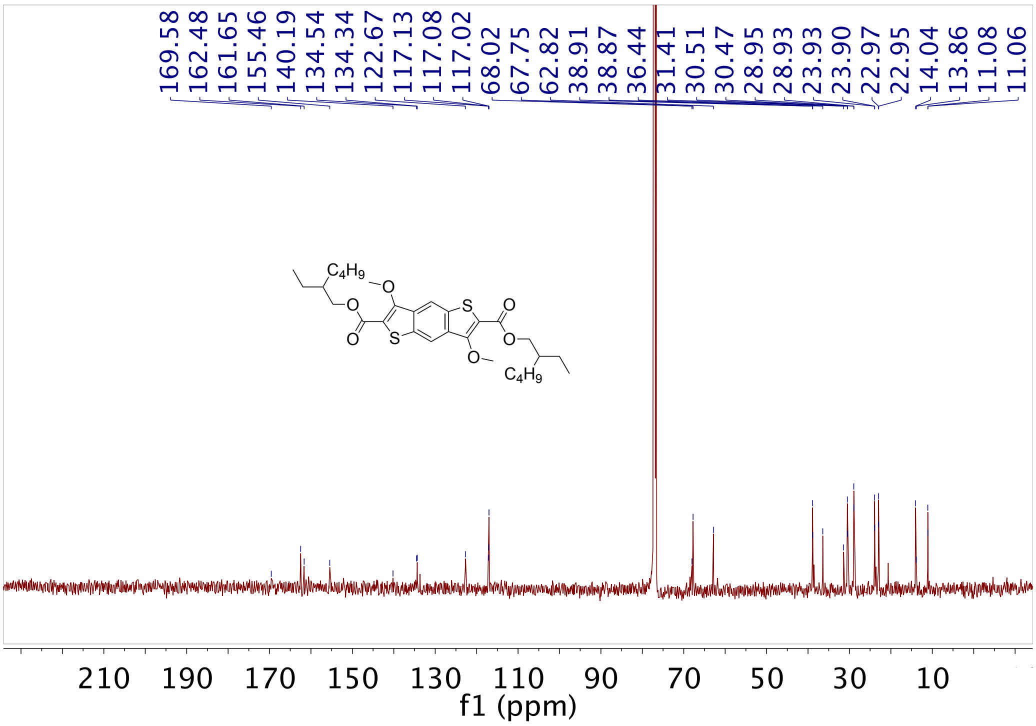

Supplementary Figure 5. ^{13}C NMR of **6** in $CDCl_3$.

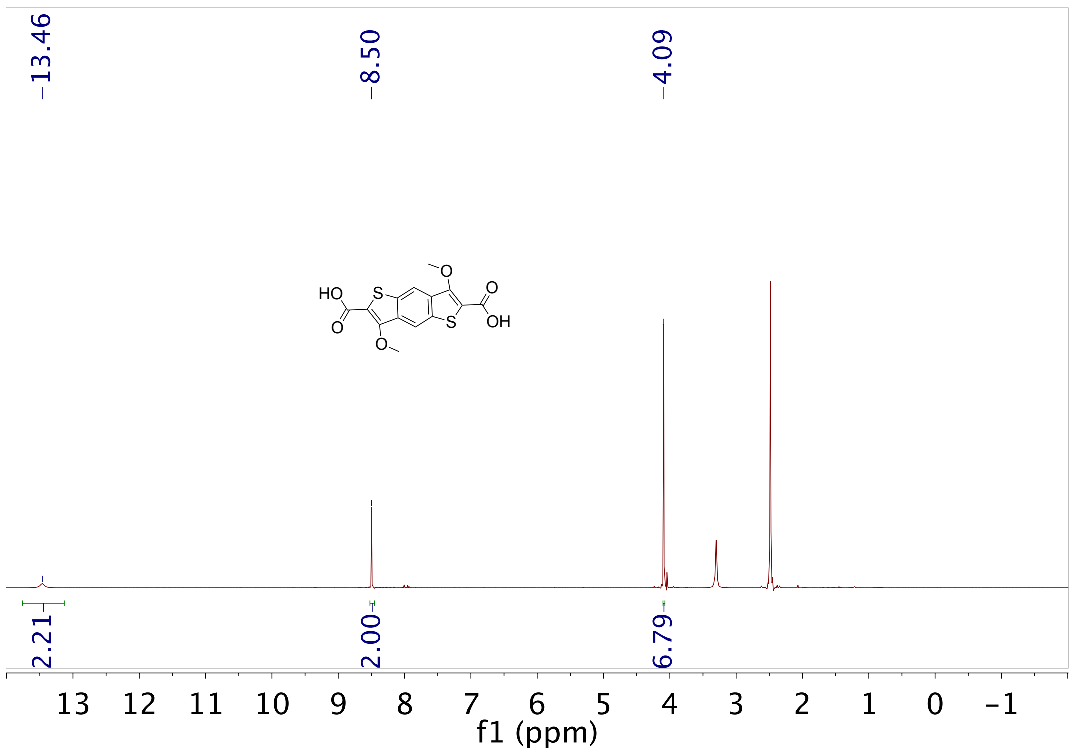

Supplementary Figure 6. ^{1}H NMR of **7** in DMSO-d_6.

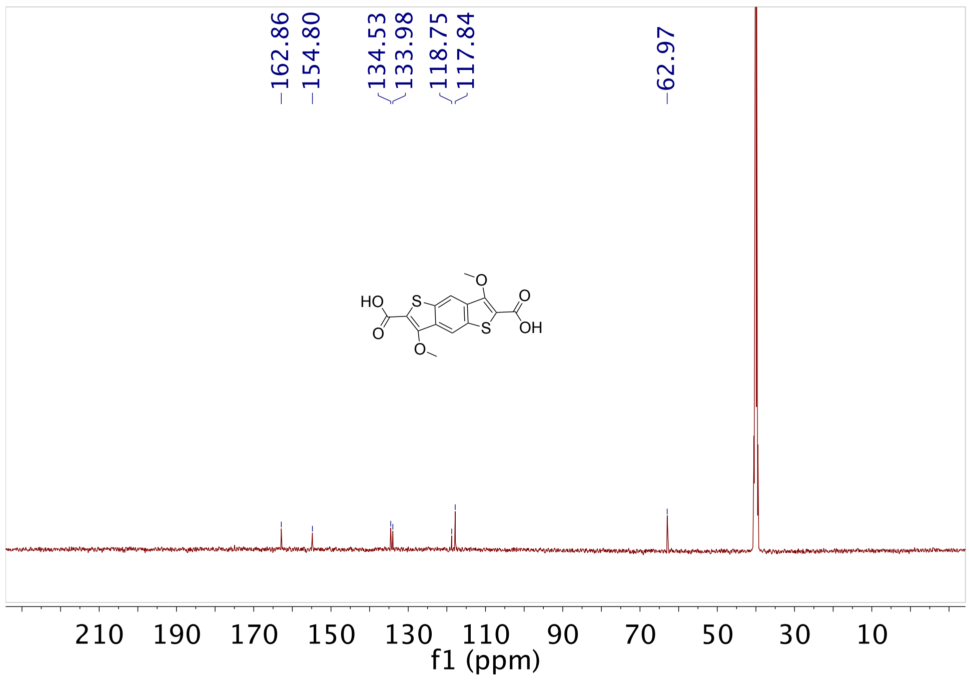

Supplementary Figure 7. ^{13}C NMR of **7** in DMSO-d_6.

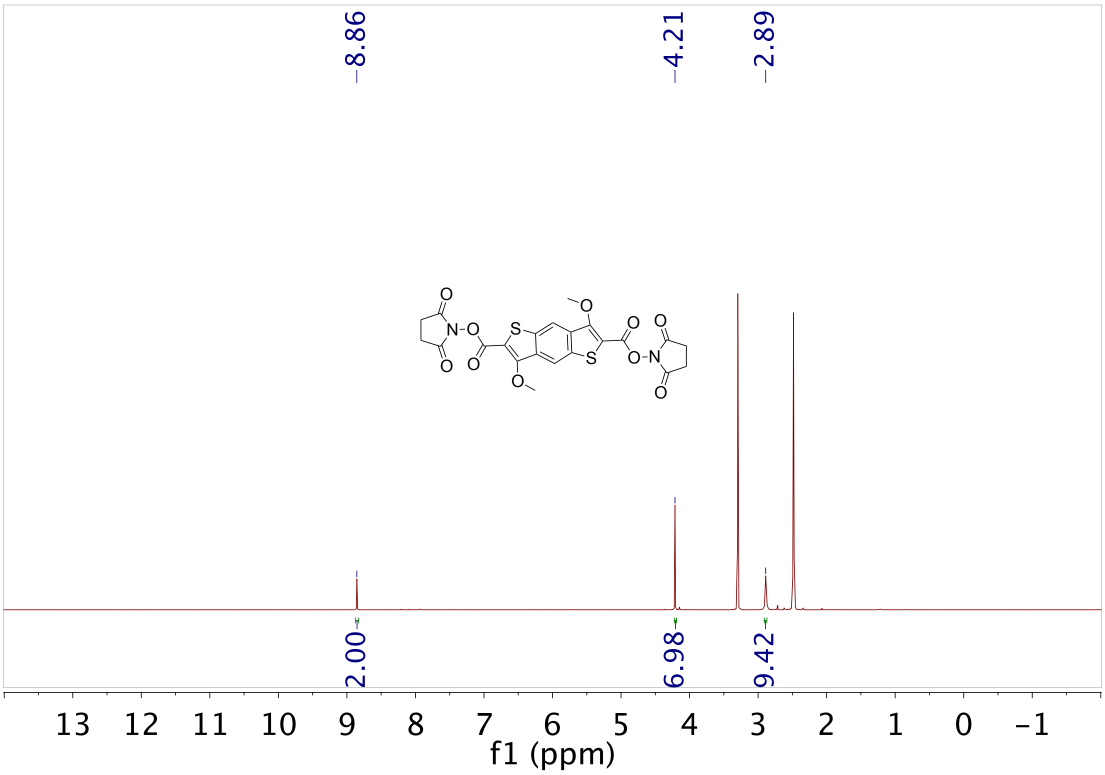

Supplementary Figure 8. ^{1}H NMR of **8** in DMSO-d_6.

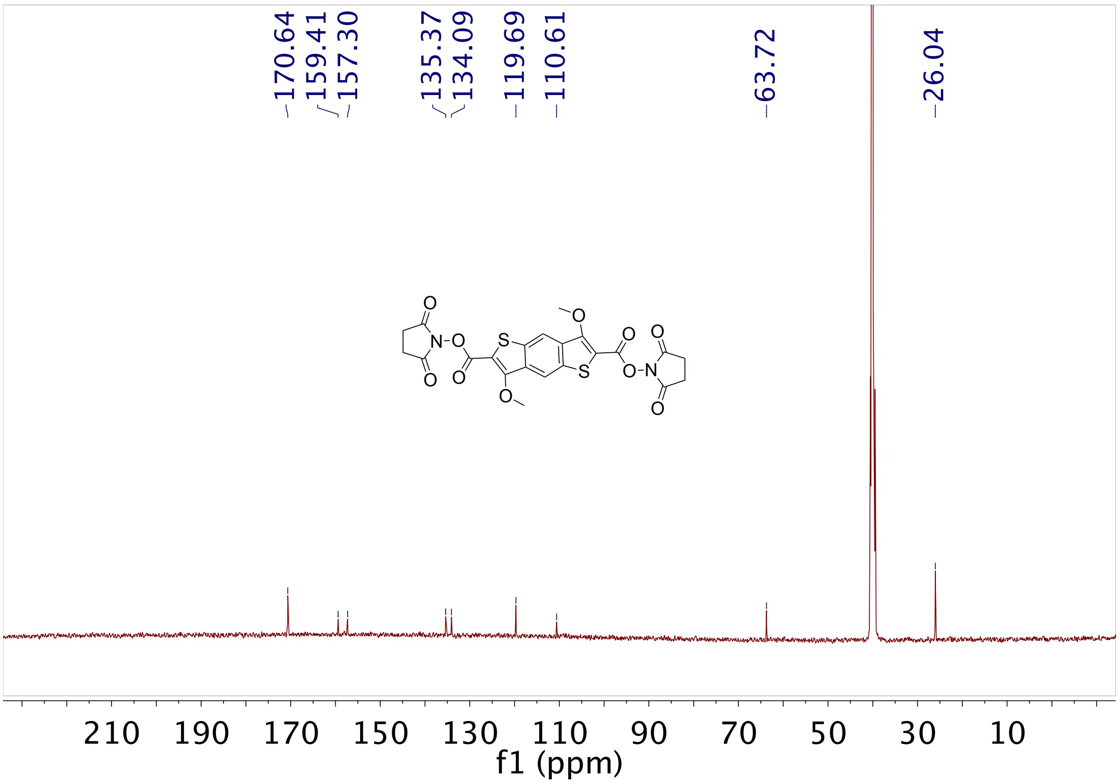

Supplementary Figure 9. ^{13}C NMR of **8** in DMSO-d_6.

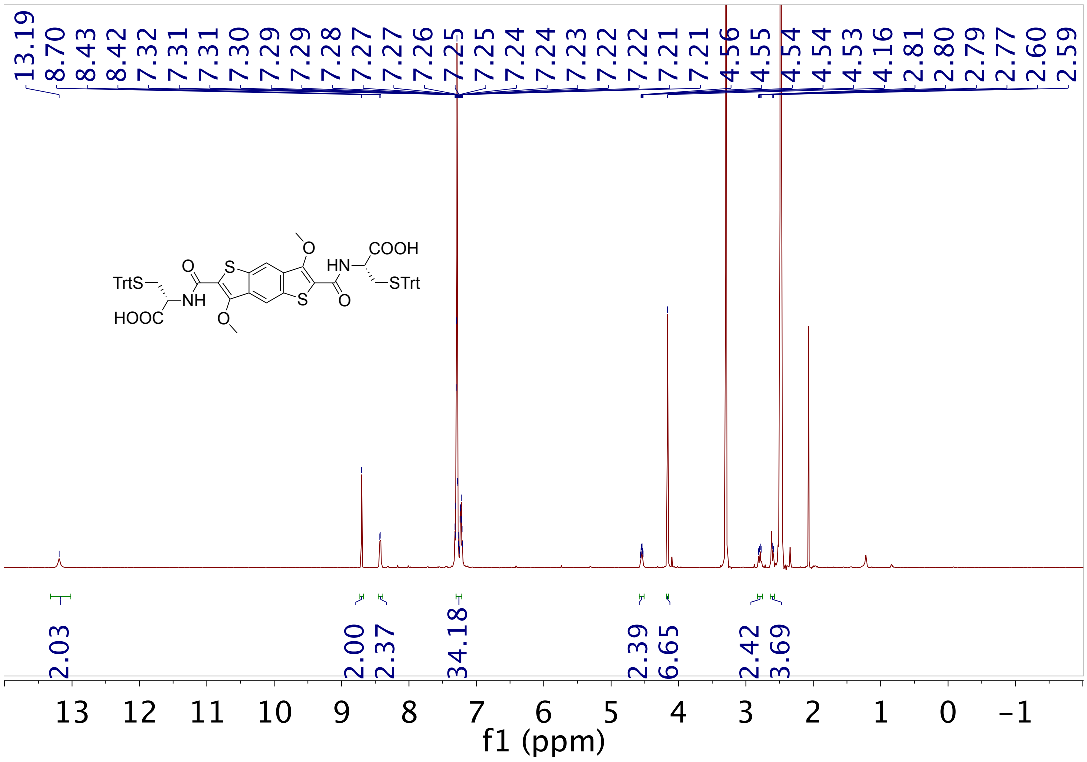

Supplementary Figure 10. ^{1}H NMR of **9** in DMSO-d_6.

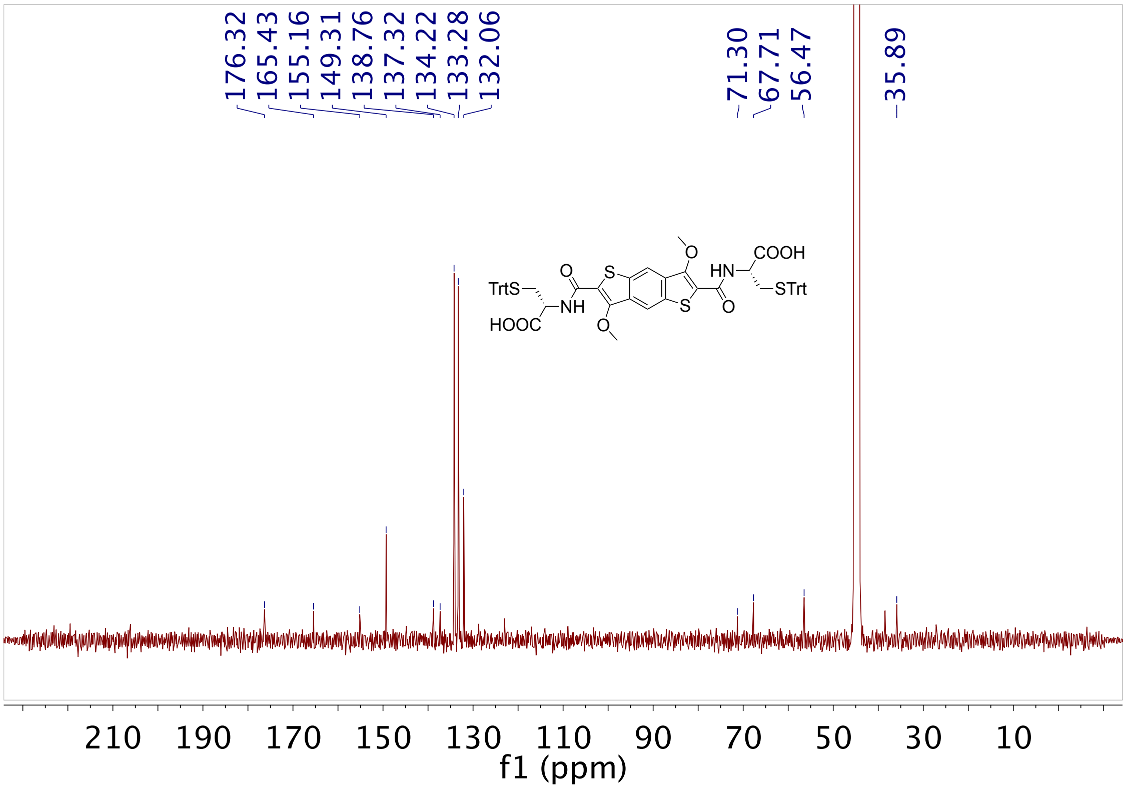

Supplementary Figure 11. ^{13}C NMR of **9** in DMSO-d_6.

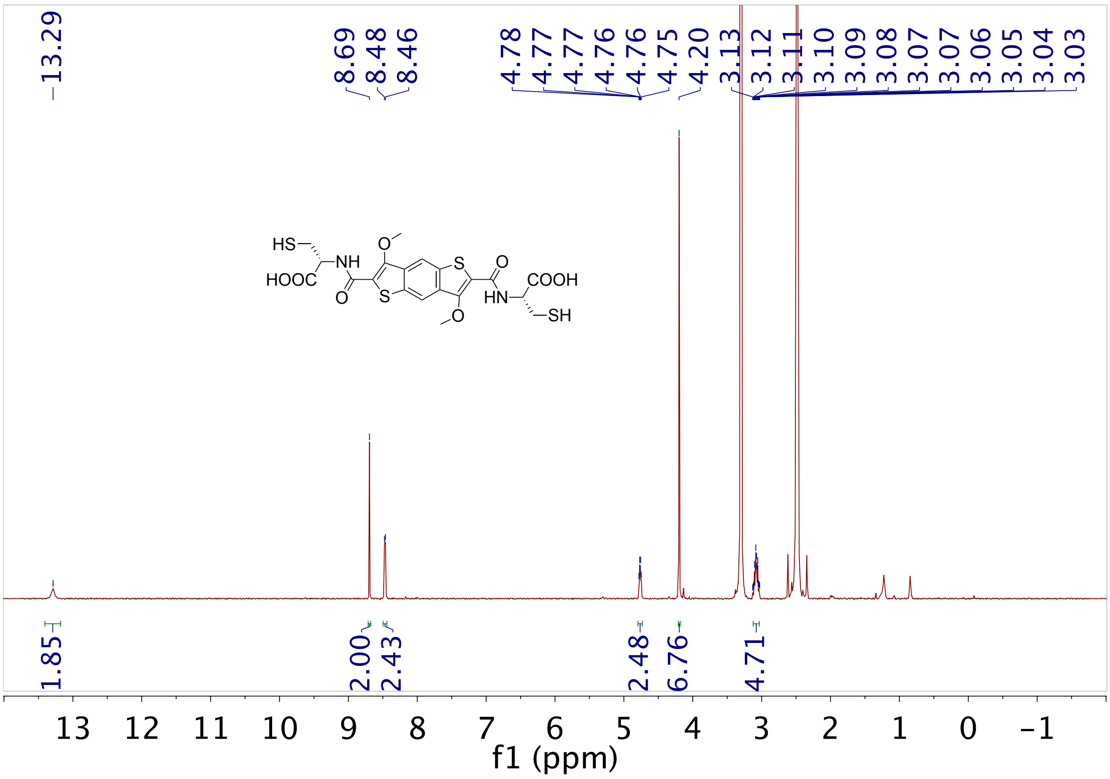

Supplementary Figure 12. ^{1}H NMR of *R,R*-**2** in DMSO-d_6.

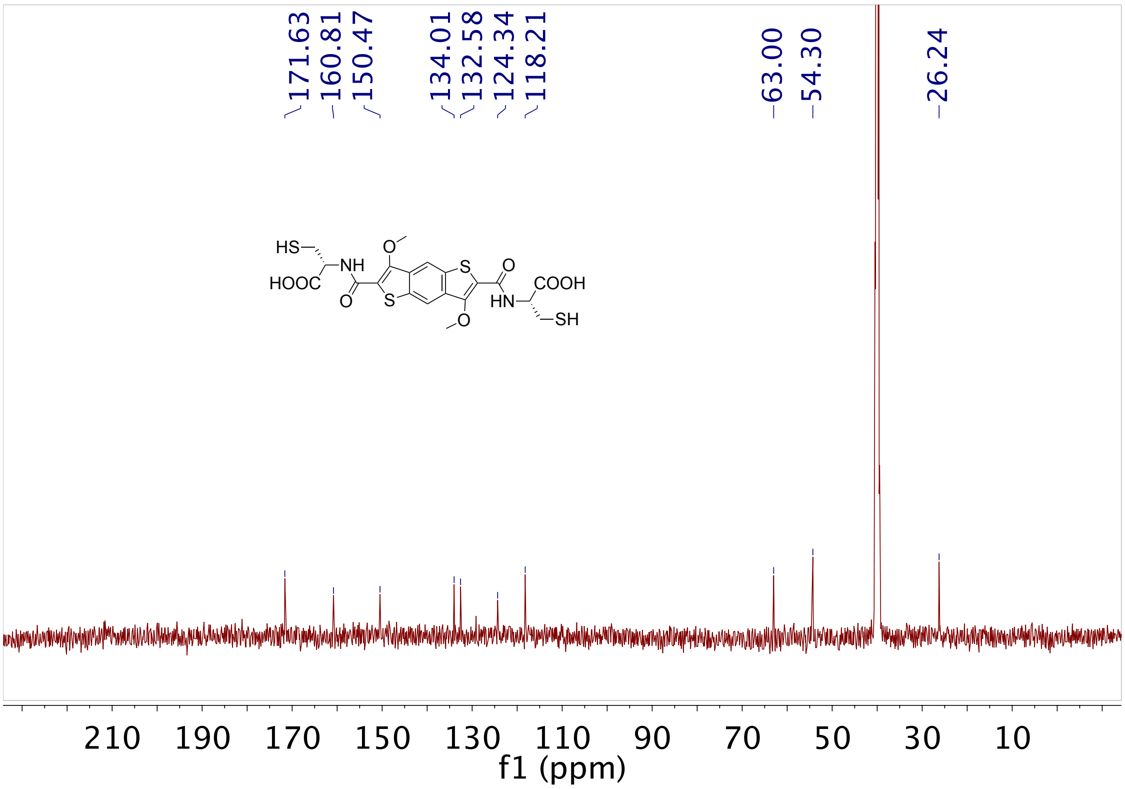

Supplementary Figure 13. ^{13}C NMR of *R,R*-**2** in DMSO-d_6.

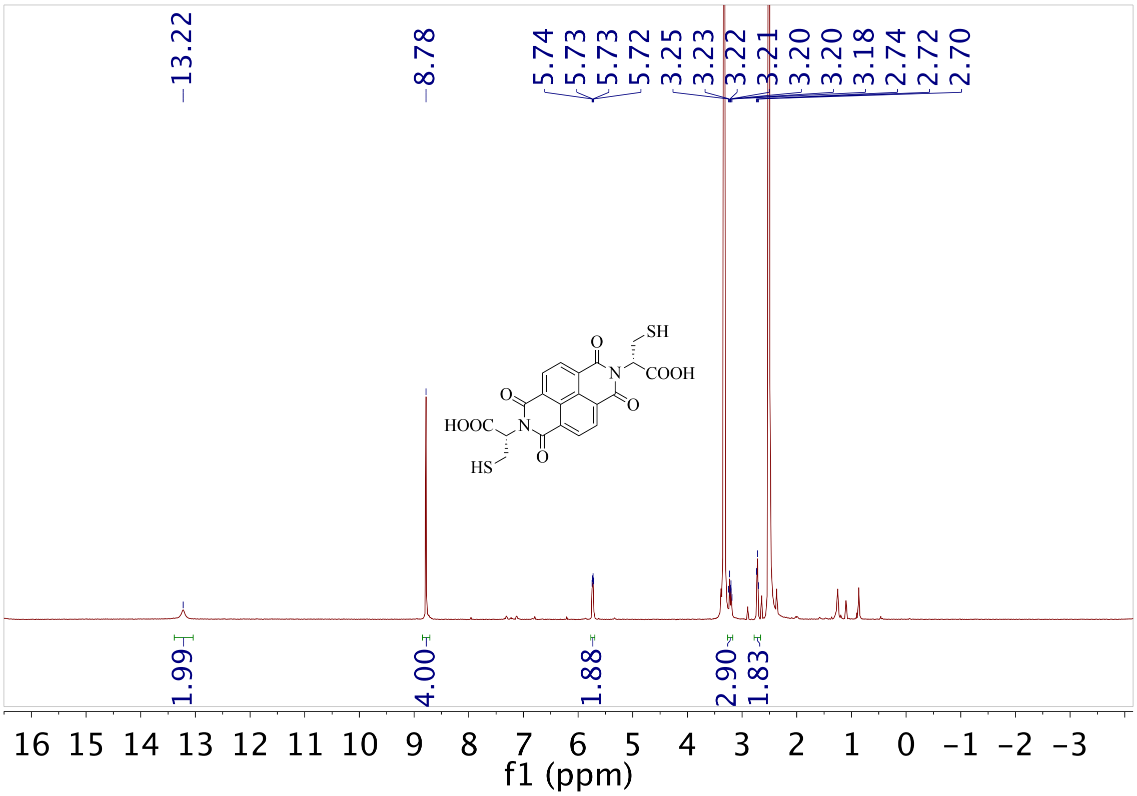

Supplementary Figure 14. ^{1}H NMR of *S,S*-***1*** in DMSO-*d*$_6$.

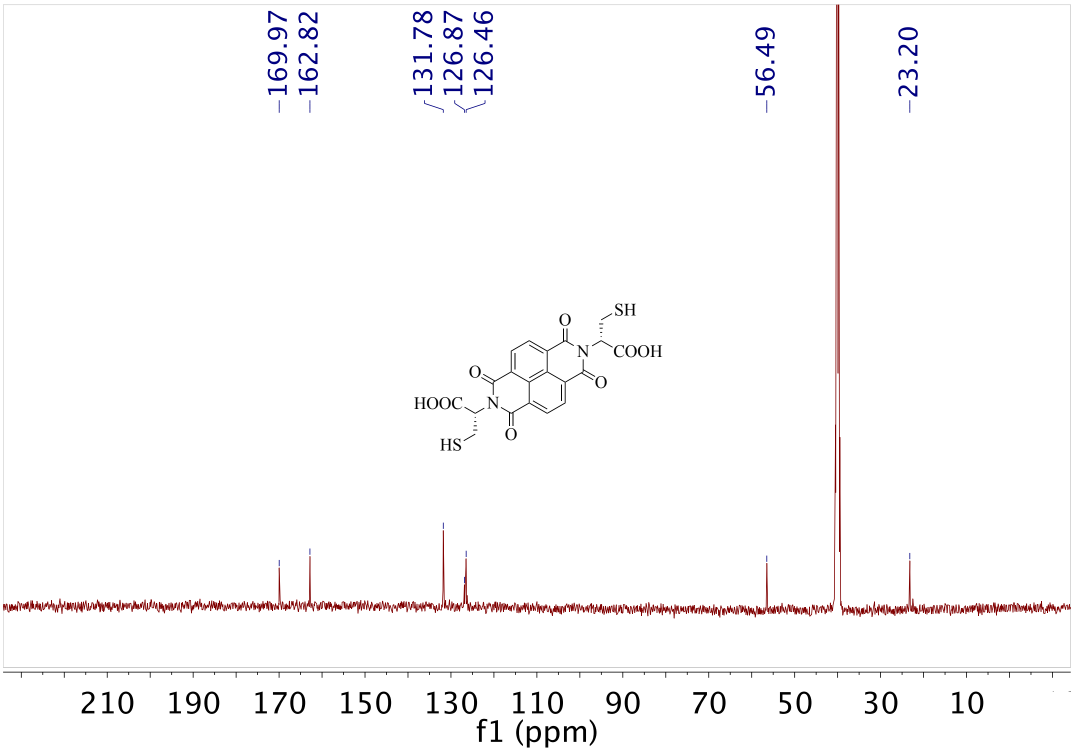

Supplementary Figure 15. ^{13}C NMR of *S,S*-***1*** in DMSO-*d*$_6$.

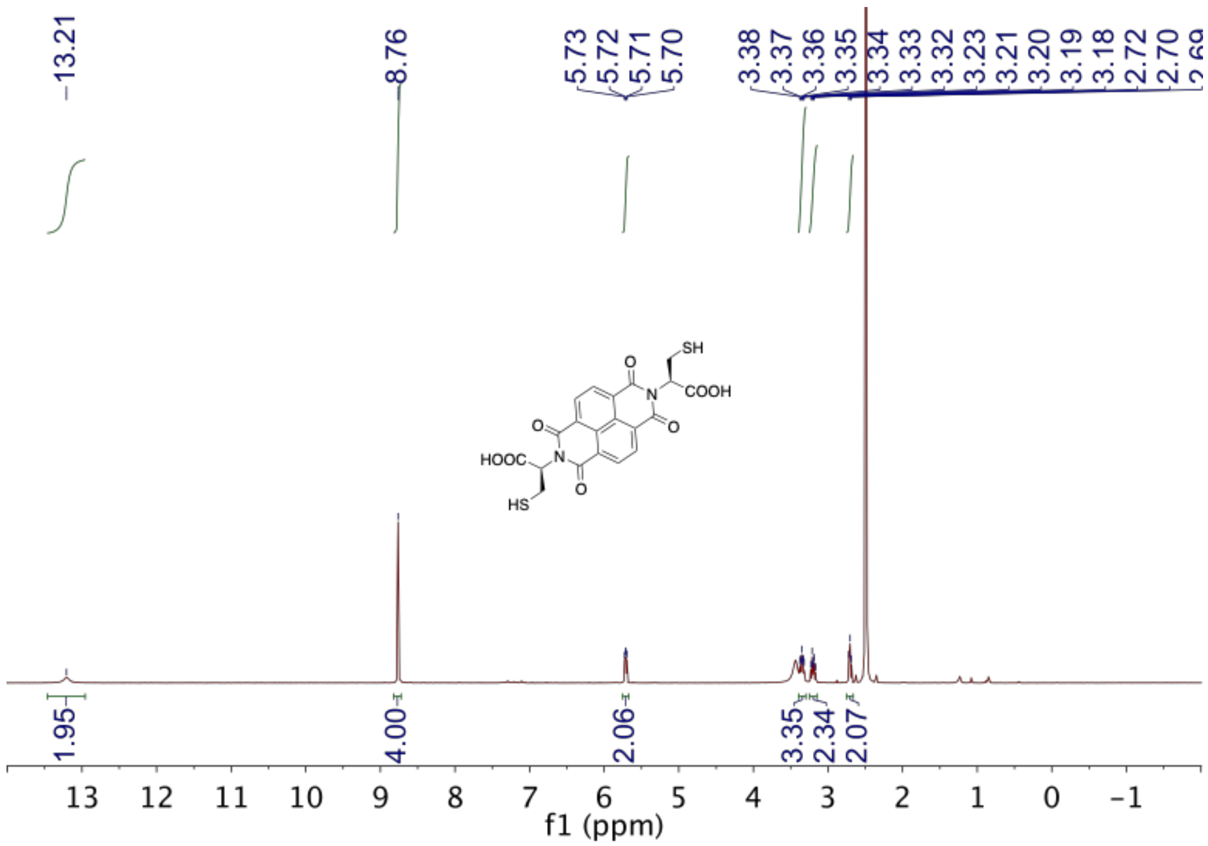

Supplementary Figure 16. ^{1}H NMR of *R,R*-**1** in DMSO-d_6.

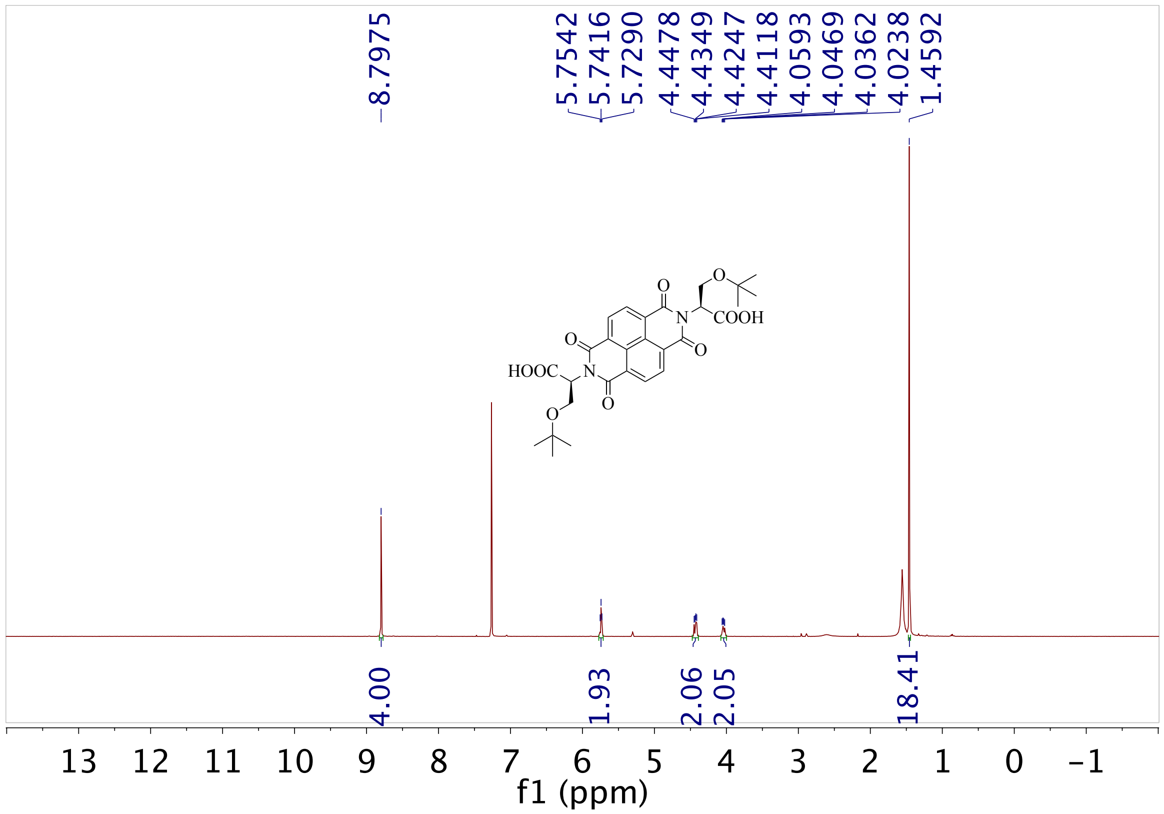

Supplementary Figure 17. ^{1}H NMR of *S,S*-**10** in $CDCl_3$.

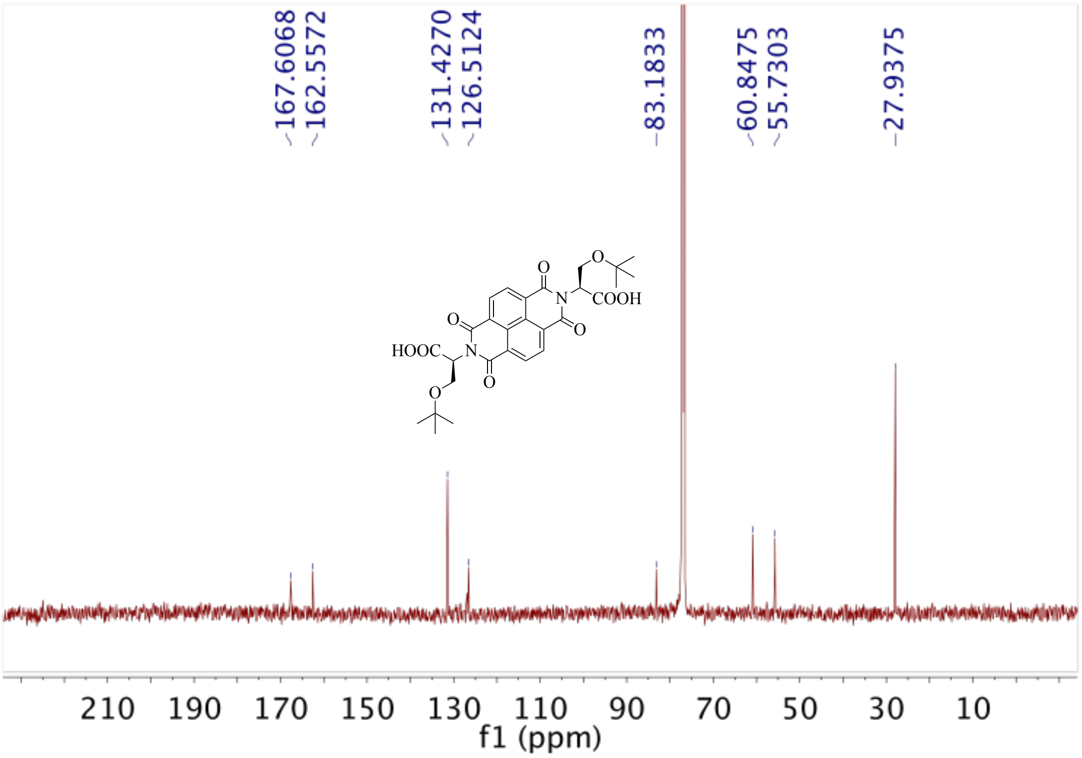

Supplementary Figure 18. ^{13}C NMR of *S,S*-**10** in $CDCl_3$.

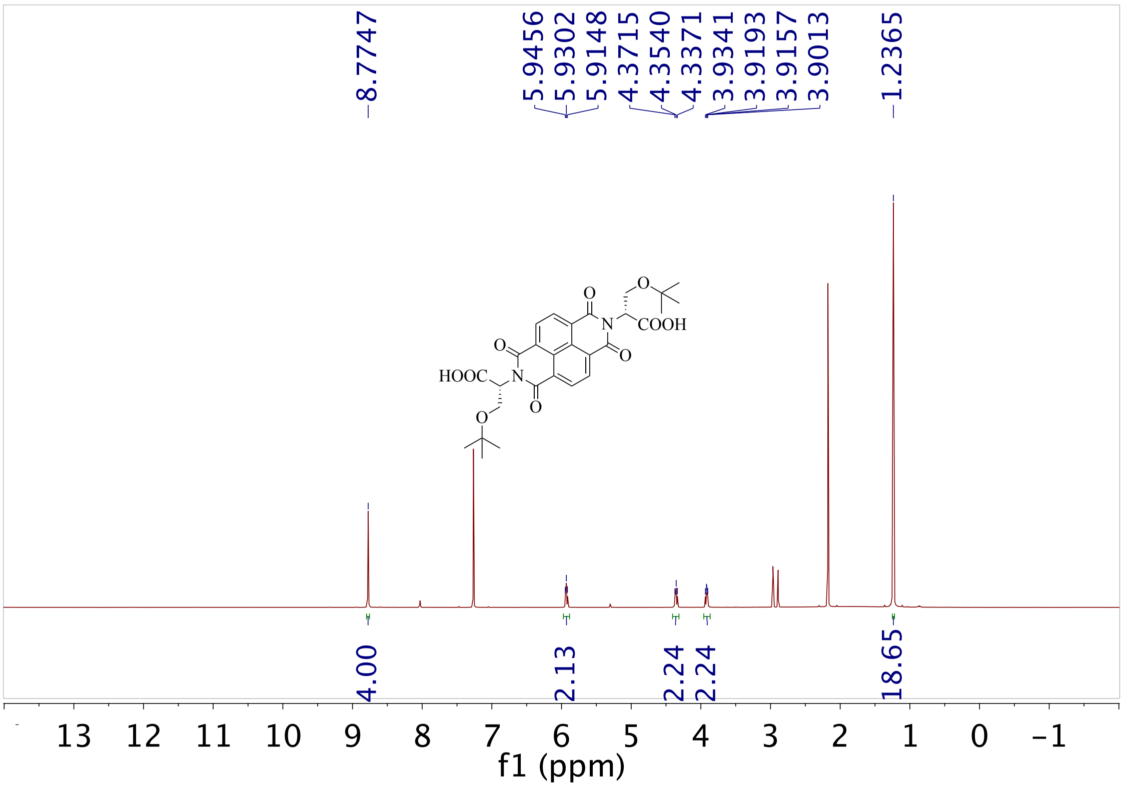

Supplementary Figure 19. ^{1}H NMR of *R,R*-**10** in $CDCl_3$.

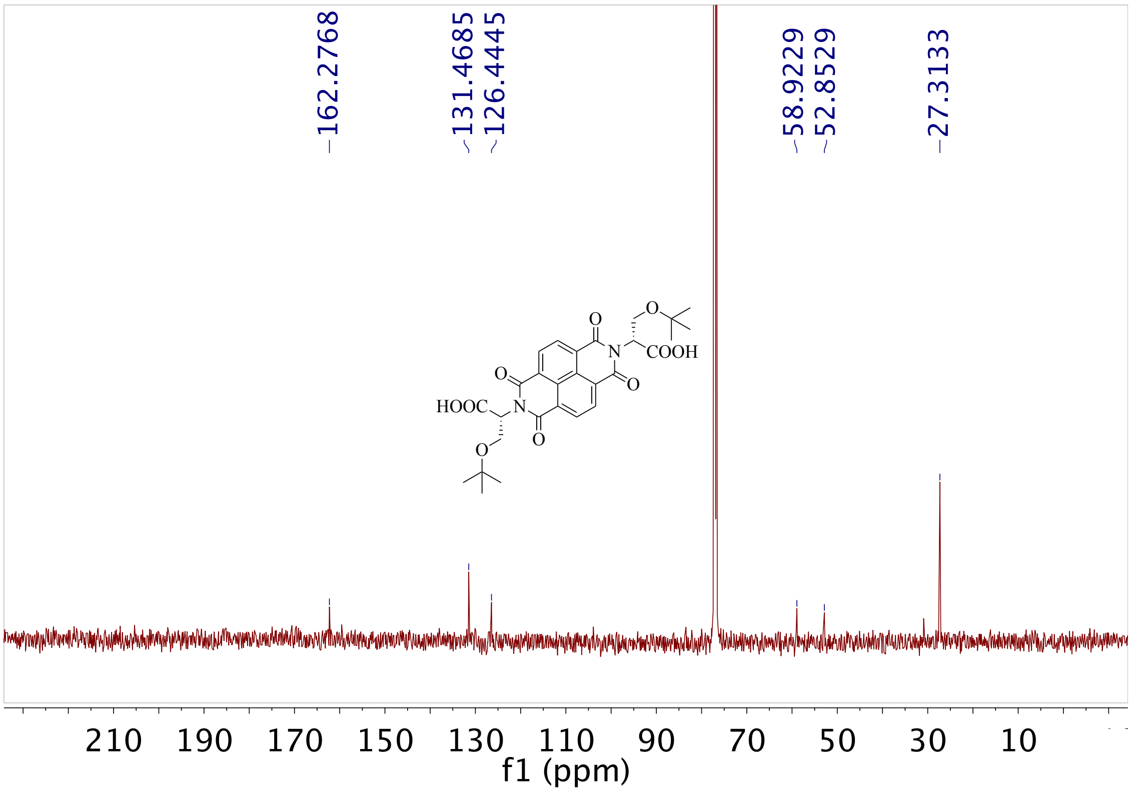

Supplementary Figure 20. ^{13}C NMR of *R,R*-**10** in $CDCl_3$.

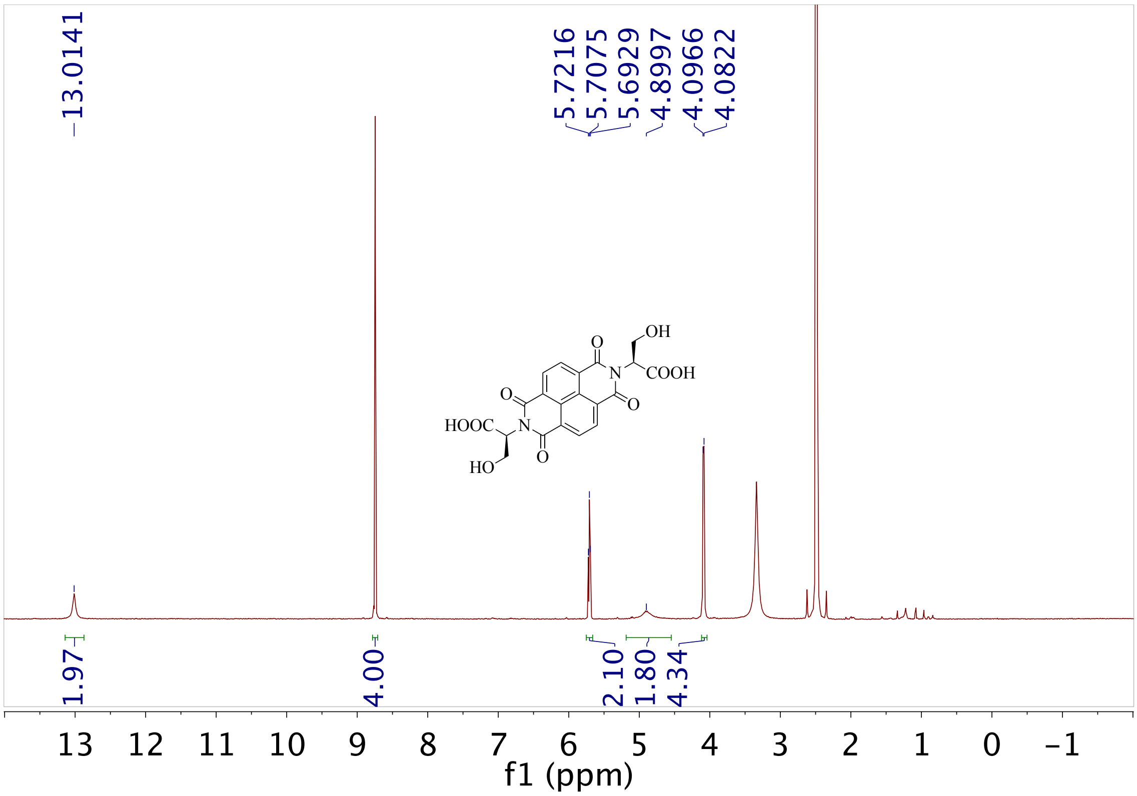

Supplementary Figure 21. ^{1}H NMR of **NDI-*S,S*-serine** in DMSO-d_6.

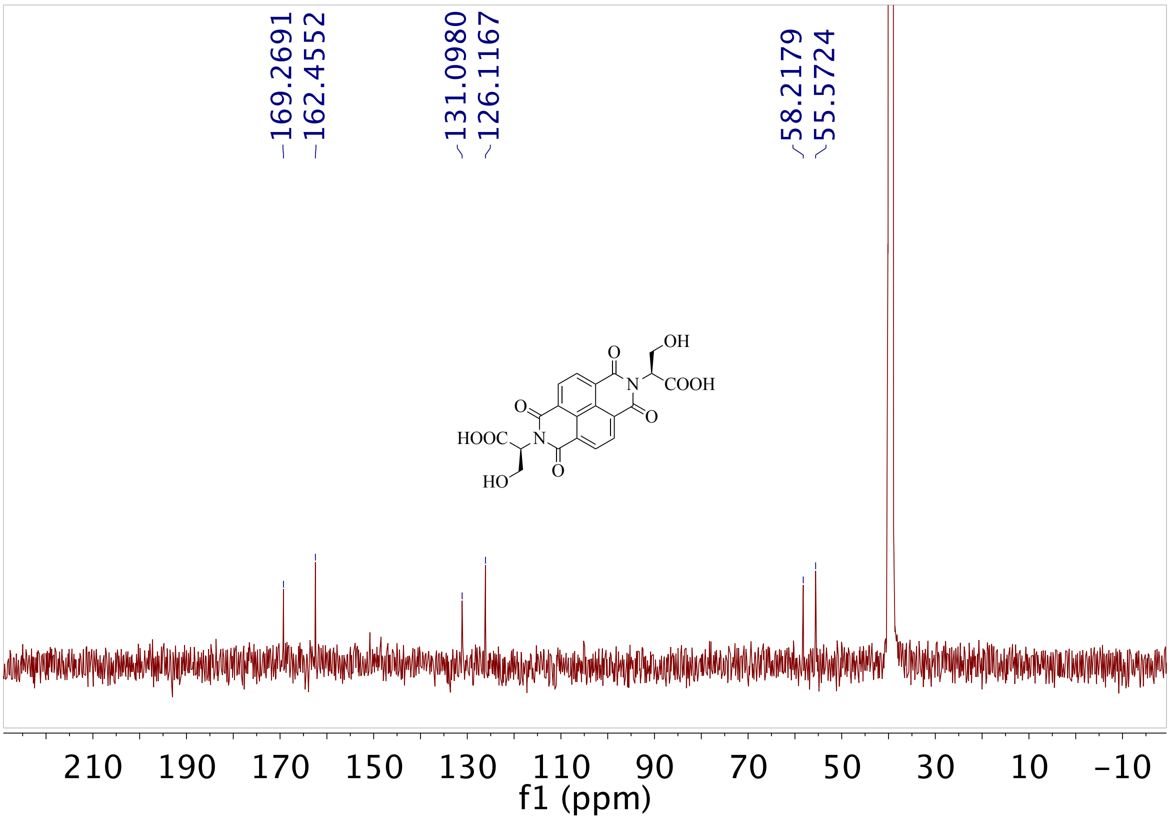

Supplementary Figure 22. ^{13}C NMR of **NDI-*S,S*-serine** in DMSO-d_6.

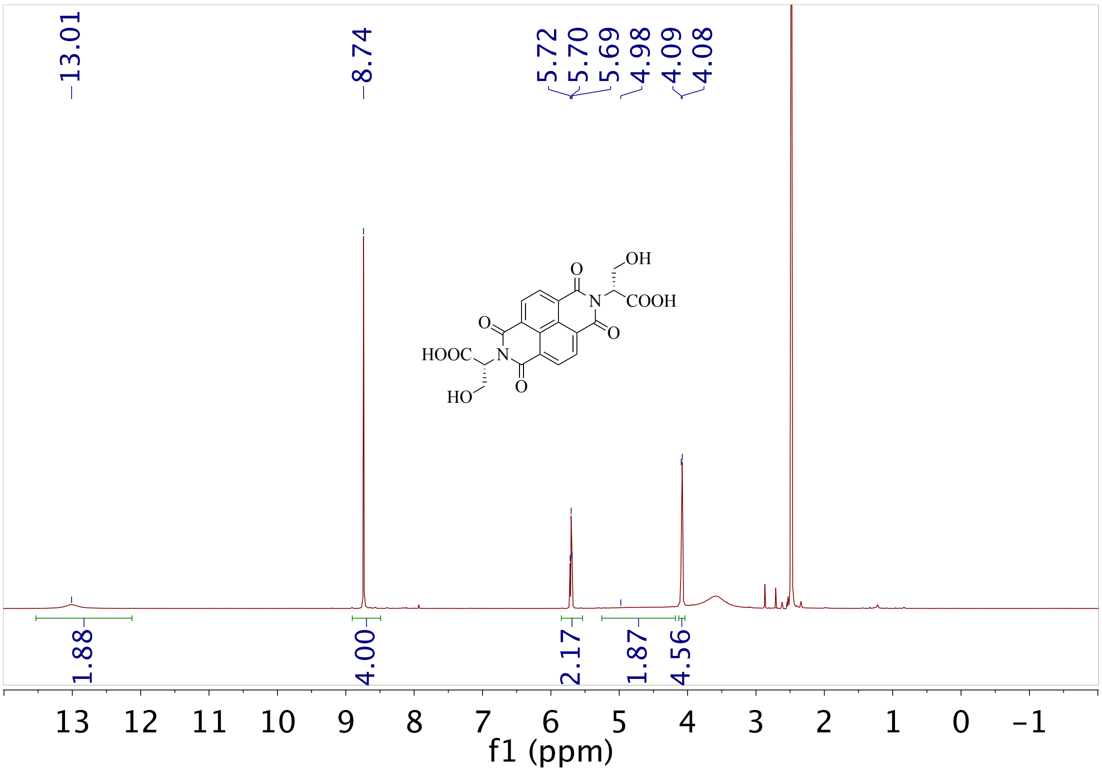

Supplementary Figure 23. ^{1}H NMR of **NDI-*R,R*-serine** in DMSO-d_6.

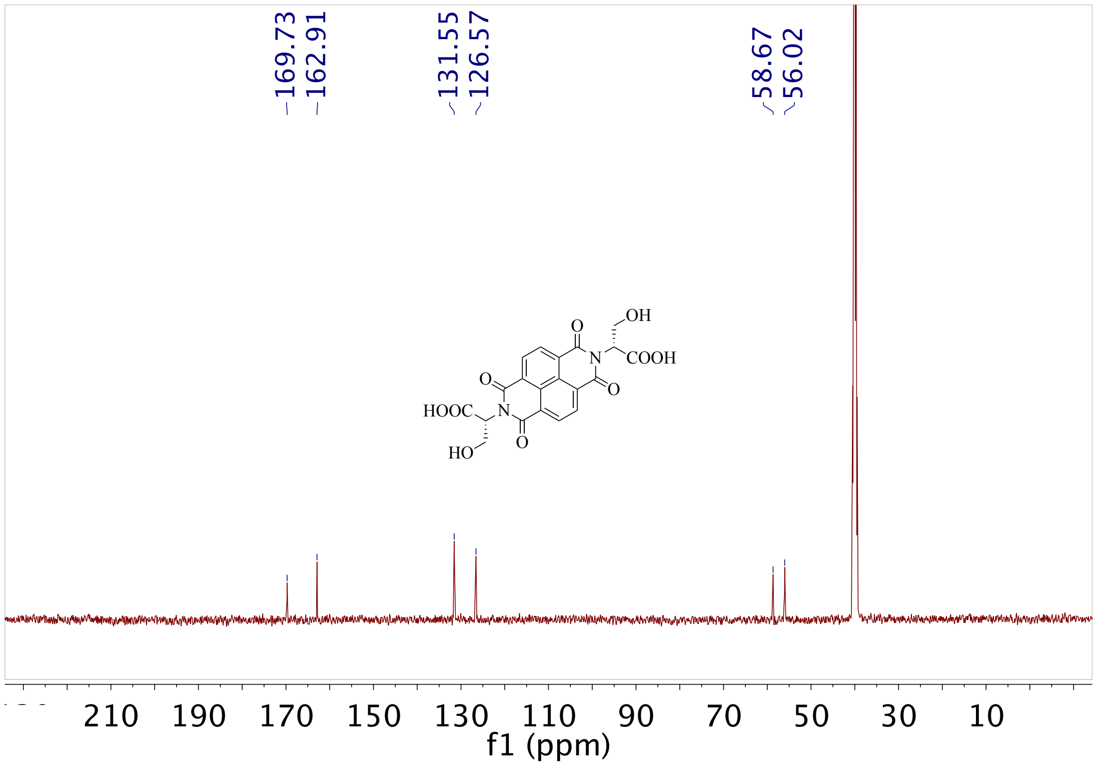

Supplementary Figure 24. ^{13}C NMR of **NDI-*R,R*-serine** in DMSO-d_6.

HPLC methods for analyzing the DCLs:

Supplementary Table 1:

Time / min	Water (0.1% Formic Acid - FA)	CH_3CN (0.1% FA)
**0**	80%	20%
**7**	40%	60%
**9**	80%	20%

Supplementary Table 2:

Time / min	Water (0.1% FA)	CH_3CN (0.1% FA)
**0**	90%	10%
**2**	90%	10%
**3**	75%	25%
**23**	58%	42%
**30**	40%	60%
**32**	35%	65%
**34**	80%	20%

Supplementary Table 3:

Time / min	Water (0.1% FA)	CH_3CN (0.1% FA)
**0**	75%	25%
**7**	40%	60%
**9**	75%	25%

Supplementary Table 4:

Time / min	Water (0.1% FA)	CH_3CN (0.1% FA)
**0**	75%	25%
**20**	55%	45%
**24**	75%	25%

Supplementary Table 5:

Time / min	Water (0.1% FA)	CH_3CN (0.1% FA)
**0**	80%	20%
**5**	75%	25%
**20**	70%	30%
**30**	65%	35%
**35**	60%	40%
**40**	50%	50%
**43**	10%	90%

Supplementary Note 1 - *Methods for the integration of the HPLC peaks:*

Solutions of identical concentrations of the building blocks (*R,R*-**1** and *R,R*-**2**) were prepared by dissolving each compound in a water:acetonitrile mixture. The UV-vis spectra of these solutions were recorded and the absorbance spectra of the two building blocks were superimposed. We chose the wavelength for which the absorbance was equal for both building blocks (in this case it was 389 nm).

First method – for well-separated peaks:

The chromatogram was loaded in XCalibur v.2.0.7 and, using the Integration function, each known (based on MS analysis) peak was integrated. The results (the values of the integrals) were exported to Excel. The value obtained for each species was divided by the number of chromophores forming the species. (*e.g.*, a catenane has 4 chromophores, so the value was divided by 4; for a dimer, we divided by 2). In this way, each adjusted integral value is directly comparable between different types of species with different numbers of chromophores. After this, the sum of all species in the chromatogram was calculated, and the value representing each individual species was divided by this sum and multiplied by 100 to get the percentage (yield) for each species.

Second method – for overlapping peaks:

QtiPlot v.8.9 was used for this method. The chromatogram was replotted in Qtiplot (the original data was exported as a text .csv file then imported in QtiPlot). Then, parts of chromatogram with overlapped peaks were analysed using Lorentzian or Gaussian deconvolution (this is an algorithm that reconstructs the entire peak which can therefore be integrated). For each of these peaks, a value representing the integrated area was generated. For the well-separated peaks in such chromatograms, the integration option in QtiPlot was used. After integrating all peaks, the data was exported to Excel and the steps from the first method were repeated: the value obtained for each species was divided by the number of chromophores forming that species. As before each adjusted value is directly comparable between different types of species with different number of chromophores. After this, the sum of all species in the chromatogram was calculated, and the value representing each individual species was divided by this sum and multiplied by 100 to get the percentage (yield) for each species.

For triplicate measurements, the areas were integrated three times. After the integration was done three times for each peak, the average for the values was calculated

- this average is the value before parentheses in the tables detailing the HPLC analyses. As for the value in parentheses, this is the RMSD value (the square root of the sum of the differences between the average and each individual value to the power of 2 divided by the square root of three).

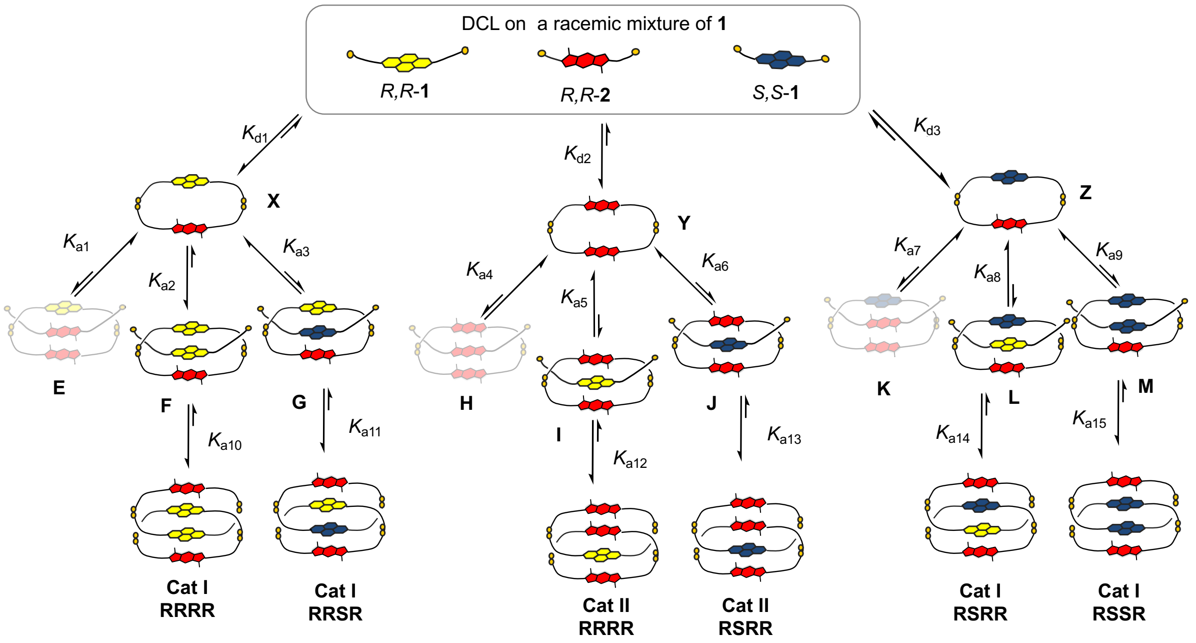

Supplementary Figure 25: Schematic representation (simplified) of the pathways leading to **Cat I RRRR** and **Cat II RSRR**. The equilibrium arrows show the relative position of the equilibrium based on theoretical and experimental data. The faded structures are either present in less than 1% or not present at all in the DCLs analysed.

Analysis of Cat I RRRR

i)

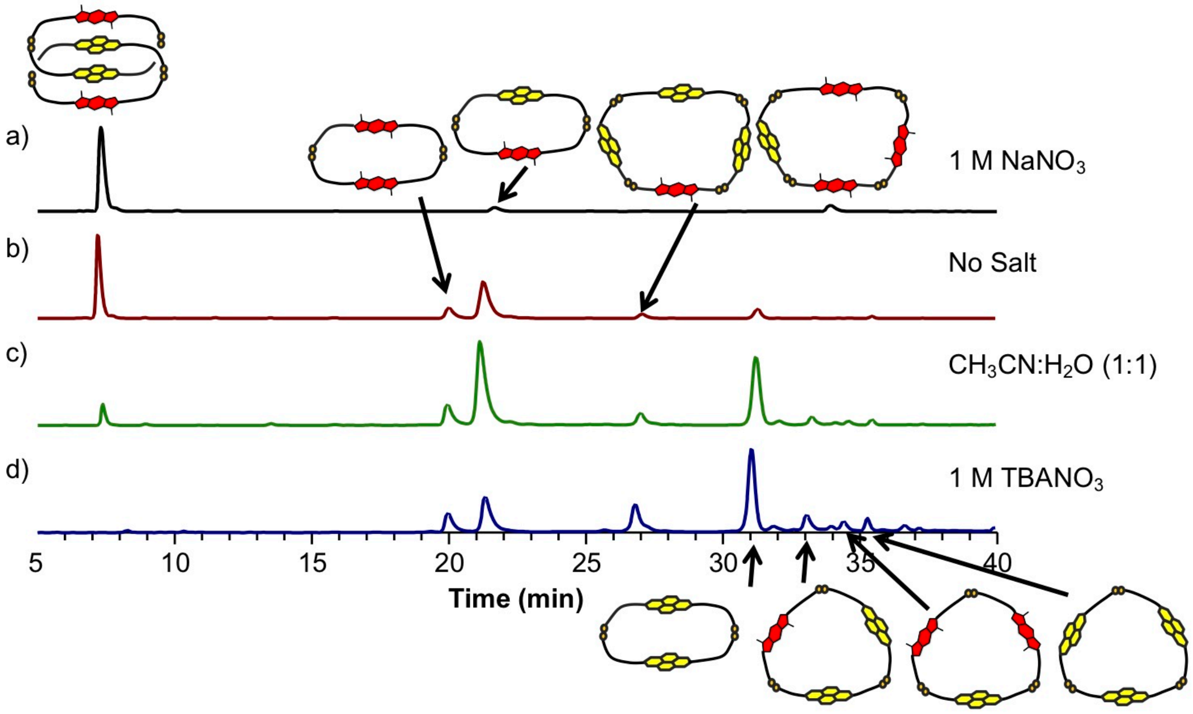

ii)

	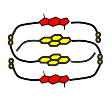	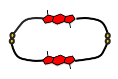	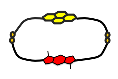	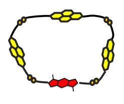	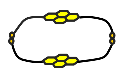	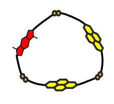	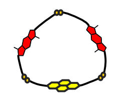	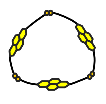	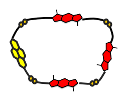
a)	75.83 (0.25)	-	16.38 (0.05)	-	-	-	-	-	7.79 (0.26)
b)	28.33 (0.19)	12.45 (0.08)	51.92 (0.25)	2.51 (0.05)	-	-	-	-	4.79 (0.04)
c)	2.83 (0.01)	10.12 (0.47)	46.90 (0.52)	3.00 (0.06)	32.35 (0.23)	2.45 (0.17)	0.94 (0.12)	1.41 (0.01)	-
d)	-	11.41 (0.20)	24.19 (0.69)	8.38 (0.19)	42.59 (0.49)	6.04 (0.25)	3.68 (0.05)	3.70 (0.02)	-

Supplementary Figure 26. i) Reverse-phase HPLC analysis of *R,R*-**1**:*R,R*-**2** (1:1 molar ratio, 5 mM total concentration) library a) in the presence of 1 M $NaNO_3$, b) no salt, c) in a mixture of $CH_3CN:H_2O$ (1:1) and d) in the presence of 1 M $TBANO_3$. Absorbances recorded at 389 nm. ii) The table shows the percentage of each species identified in the chromatograms above (i). The integrations were done in triplicate and the RMSD is reported in parenthesis along with the value. Each row of the table corresponds to the chromatogram bearing the same identification in the figure above. The unlabelled peaks did not ionise and could not be identified.

From these experiments, we can conclude that $K_{d1}>K_{d2}$ (from **Supplementary Figure 25**).

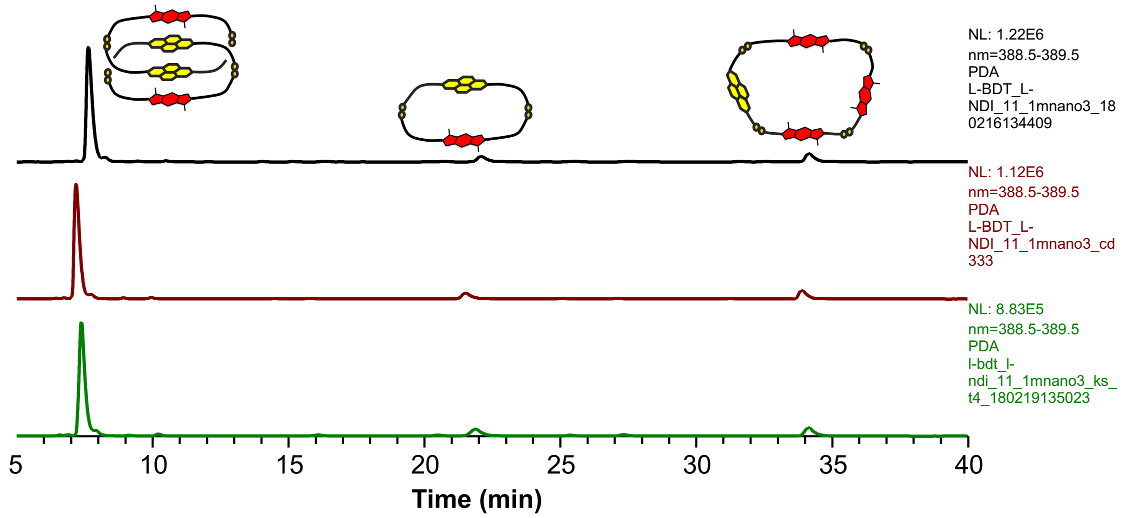

Supplementary Figure 27. Reverse-phase HPLC analysis of *R,R*-**1**:*R,R*-**2** (1:1 molar ratio, 5 mM total concentration) library in the presence of 1 M $NaNO_3$ of different libraries and/or the same library analysed at different times.

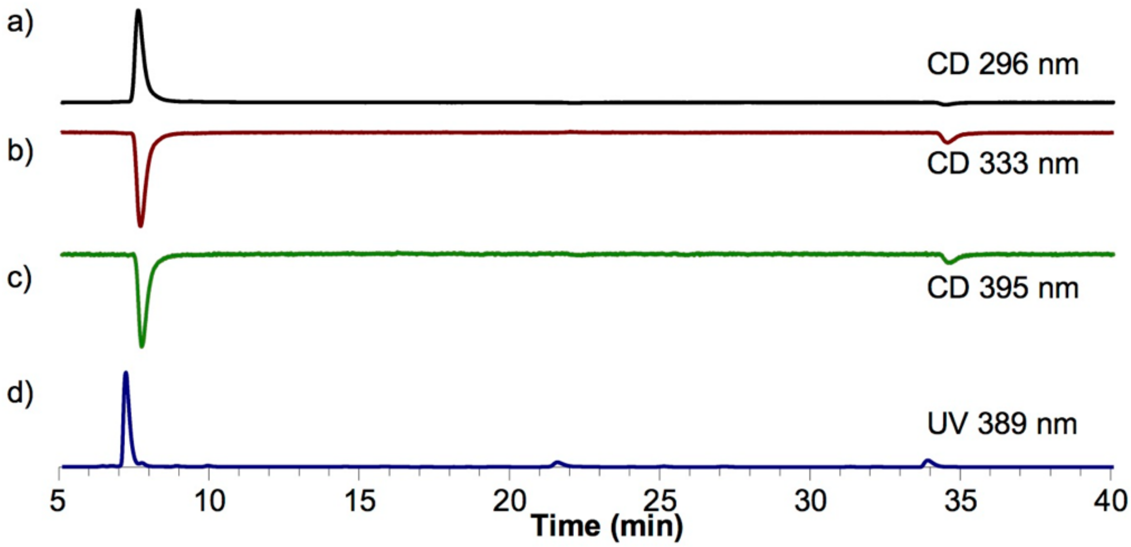

Supplementary Figure 28. Reverse-phase HPLC analysis of *R,R*-**1**:*R,R*-**2** (1:1 molar ratio, 5 mM total concentration) library in the presence of 1 M $NaNO_3$, showing: a) CD at 296 nm, b) CD at 333 nm and c) CD at 395 nm, d) absorbance at 389 nm.

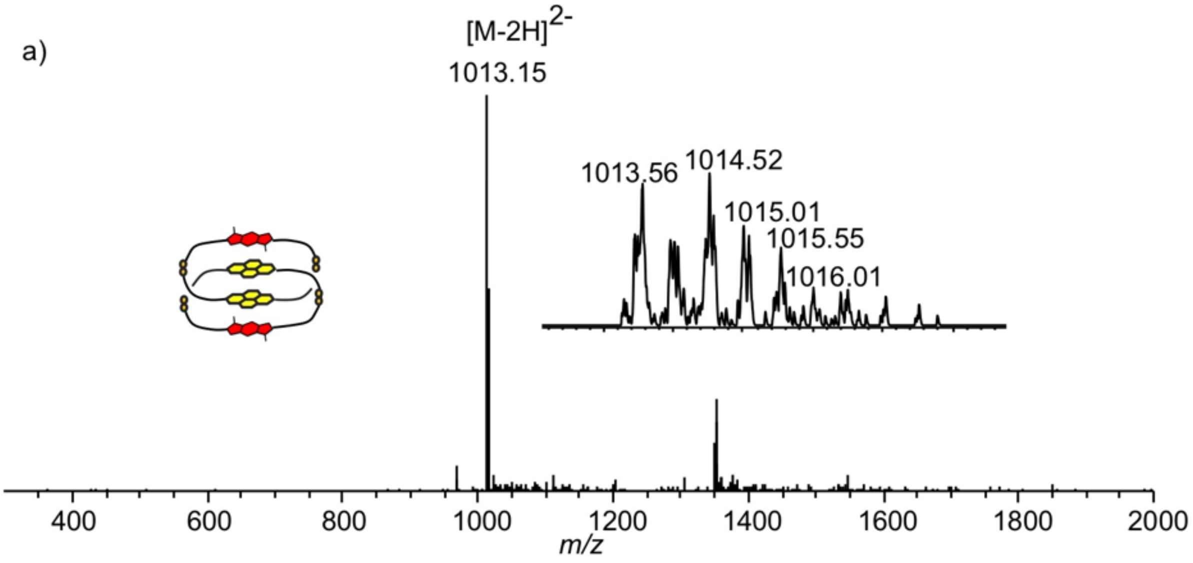

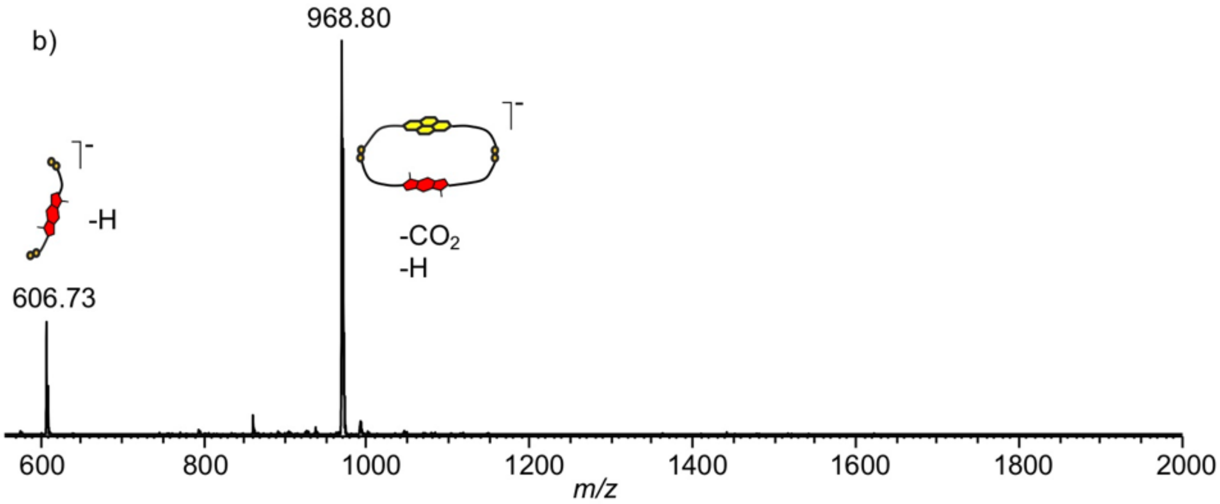

Supplementary Figure 29. a) MS (-*ve*) of **Cat I RRRR**; zoom of molecular ion is shown as inset. b) MS/MS (-*ve*) of **Cat I RRRR**. The presence of perthiolate was previously reported.[2]

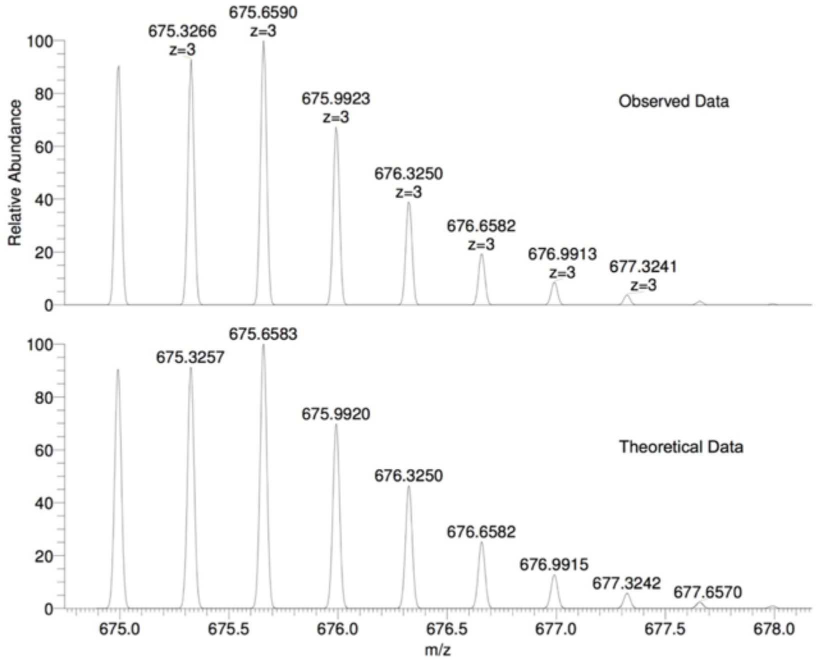

Supplementary Figure 30. High resolution MS of **Cat I RRRR**.

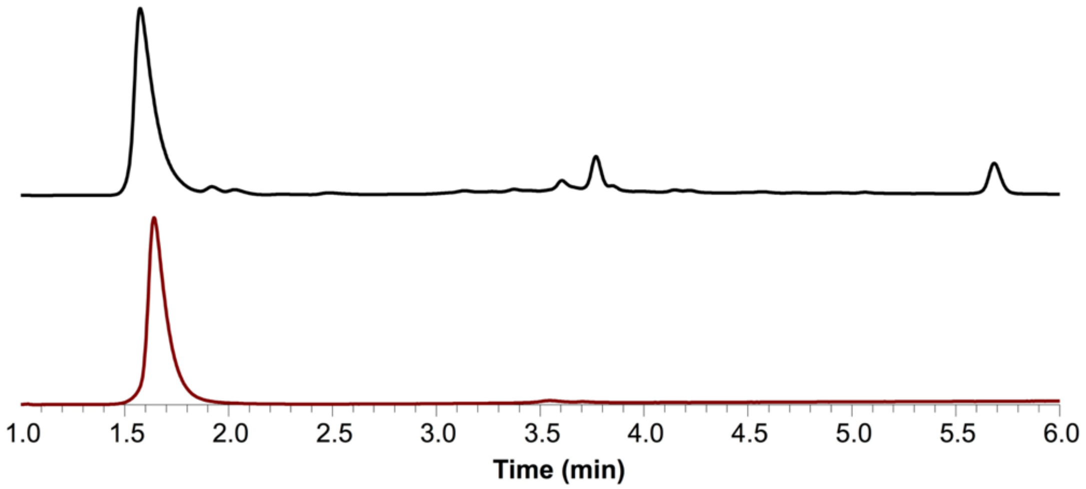

Supplementary Figure 31. Reverse-phase HPLC analysis of *R,R*-**1**:*R,R*-**2** (1:1 molar ratio, 5 mM total concentration) library (black trace) and **Cat I RRRR** after isolation (red trace). Absorbances recorded at 389 nm.

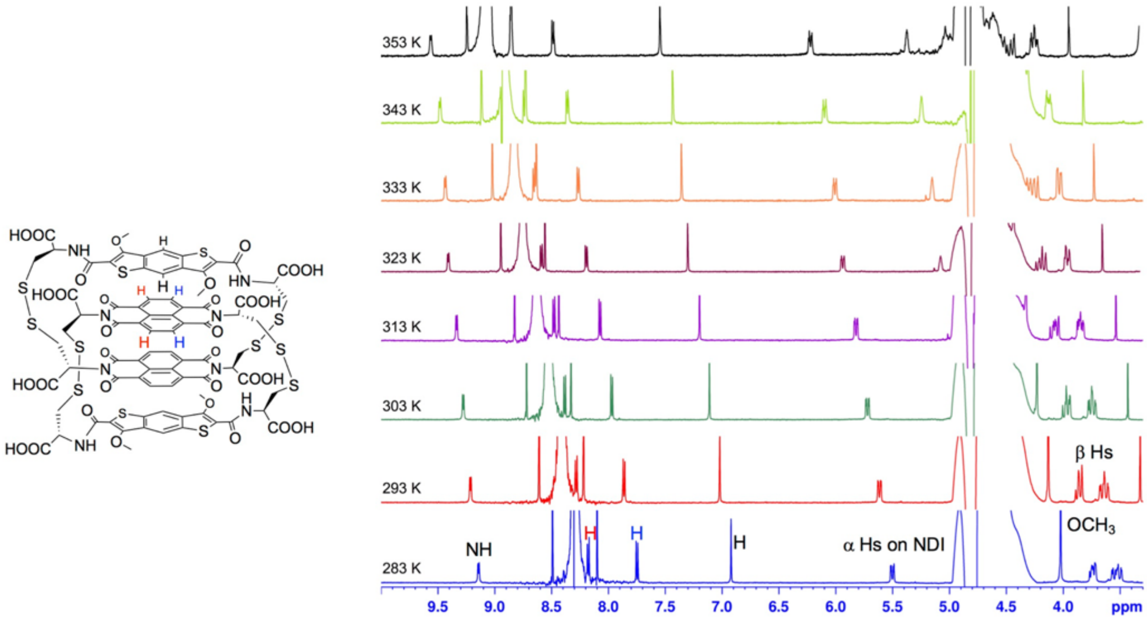

Supplementary Figure 32. Variable temperature ^{1}H NMR spectra (500 MHz) of **Cat I RRRR** at the temperatures indicated above each spectrum. The solvent (H_2O) was referenced at 4.79 ppm.

The VT-NMR experiment is useful in confirming that the structure is a catenane. In the case of an isomeric macrocycle, the ^{1}H NMR peaks would get broader as the temperature increases due to high thermal energy. The sharpness of the peaks and the relative constant shift as the temperature increases is supportive of an interlocked geometry for this molecule.

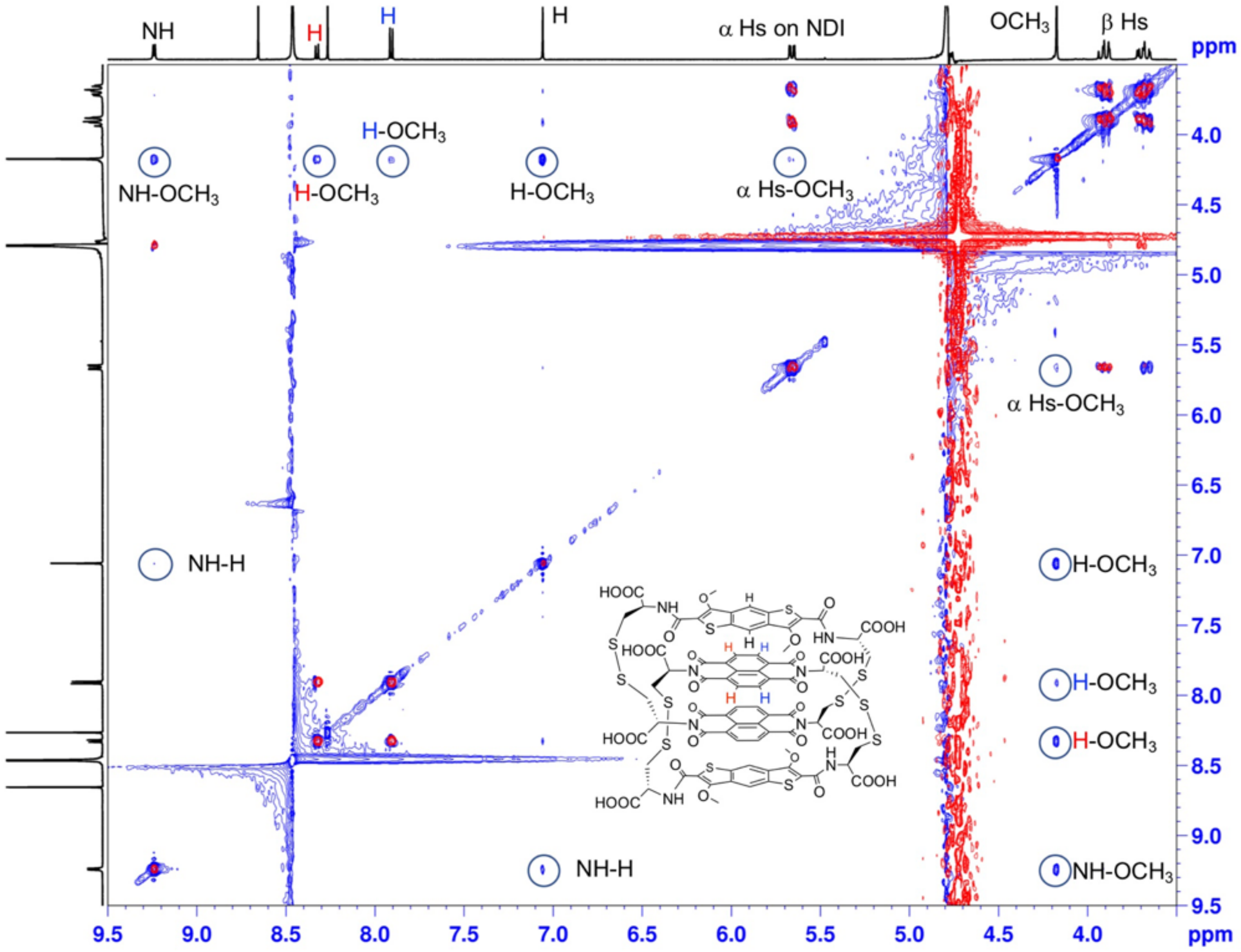

Supplementary Figure 33. ^1H-^1H COSY (red) and 2D NOESY (blue) spectra (500 MHz, 298 K) superimposed of **Cat I RRRR**. The solvent (H_2O) was referenced at 4.79 ppm.

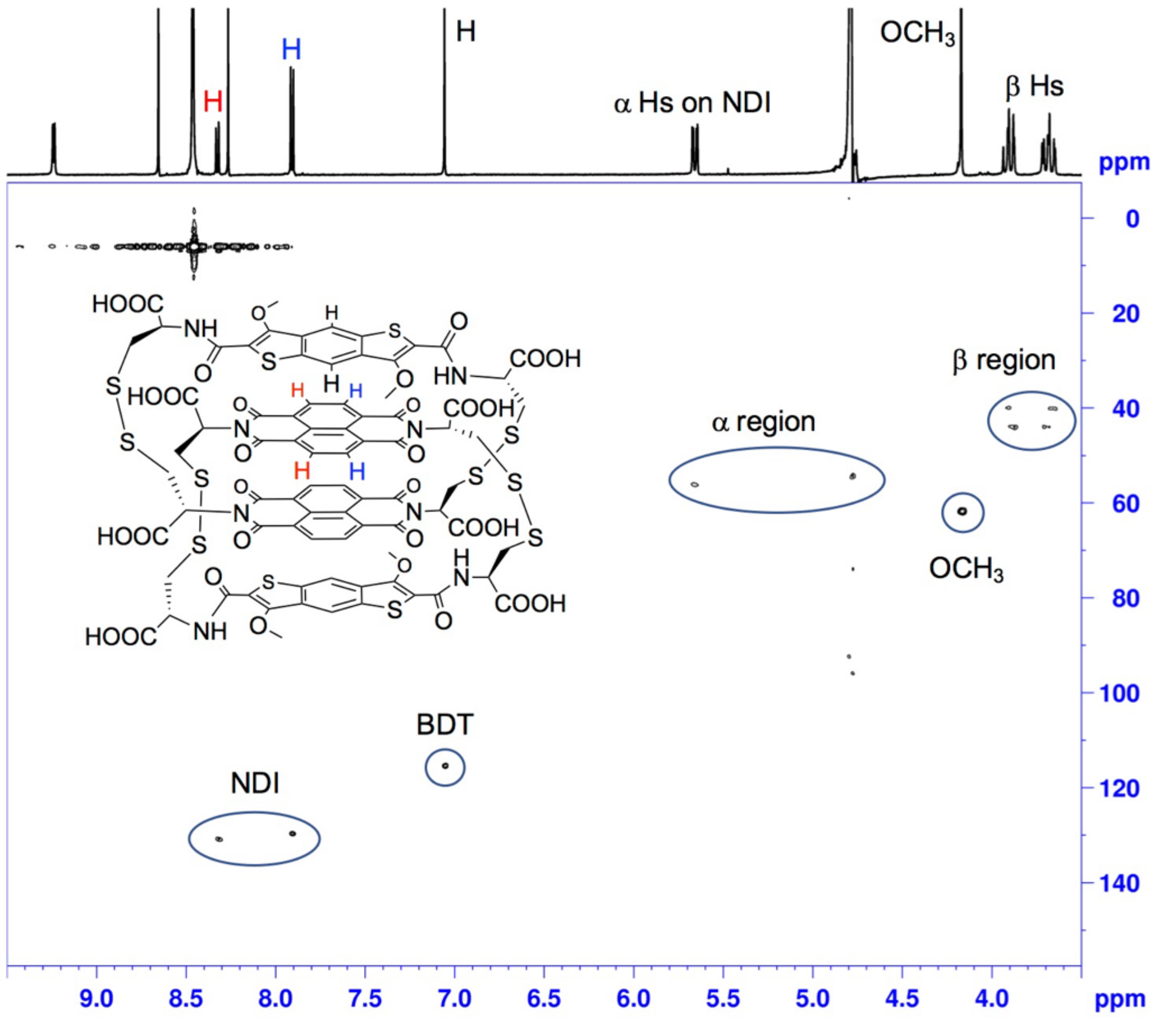

Supplementary Figure 34. HSQC spectrum (500 MHz, 298 K) of **Cat I RRRR.** The solvent (H_2O) was referenced at 4.79 ppm.

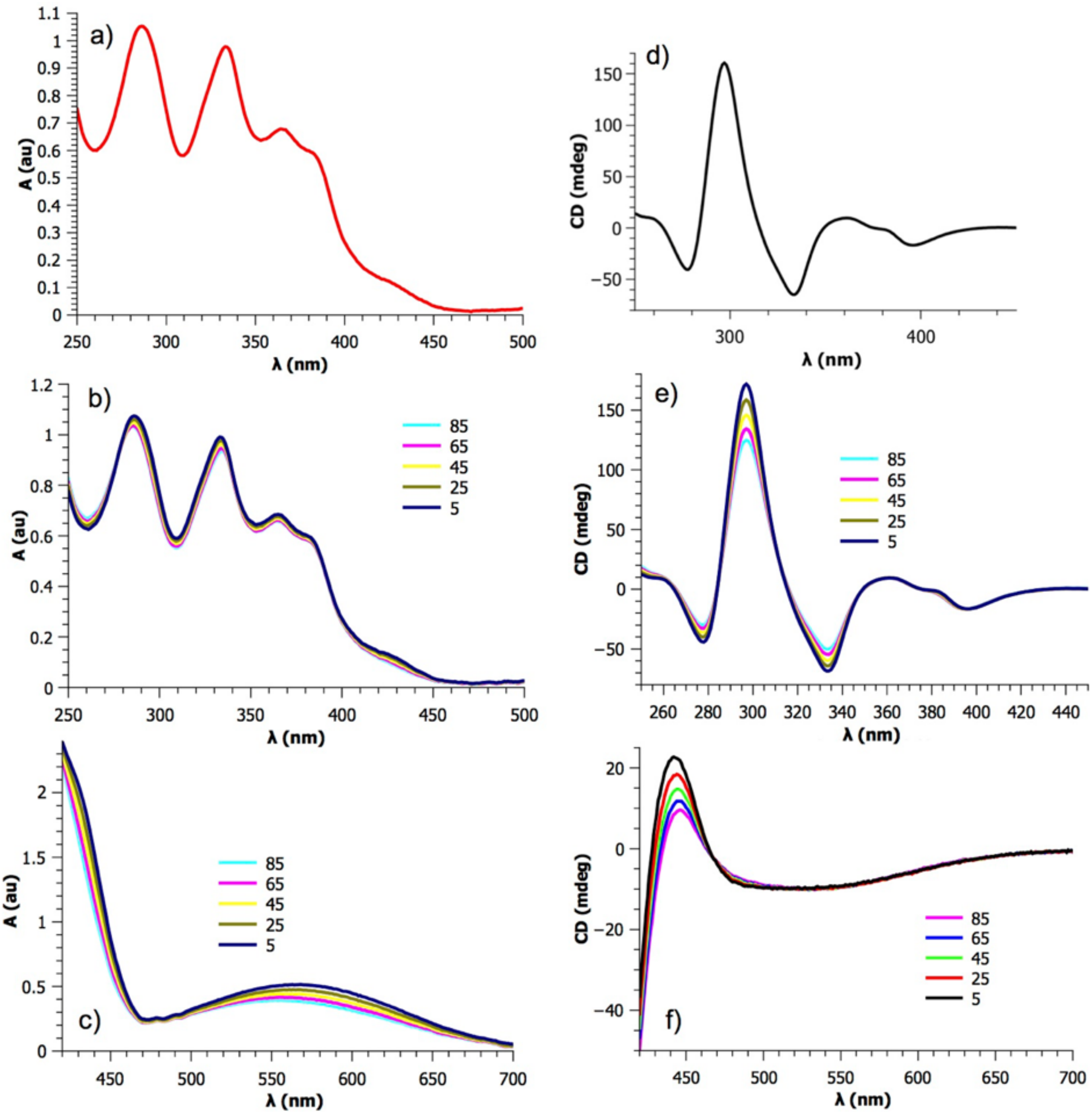

Supplementary Figure 35. a) UV-Vis spectrum of **Cat I RRRR** at 23 °C (296 K), b) VT UV-Vis spectra of **Cat I RRRR**, c) VT UV-Vis spectra of **Cat I RRRR** in CT region between 5 – 85 °C at the specified temperatures, d) CD spectrum of **Cat I RRRR** at 23 °C; e) VT CD spectra of **Cat I RRRR**, c) VT CD spectra of **Cat I RRRR** in CT region between 5 – 85 °C at the specified temperatures.

Analysis of Cat II RSRR

i)

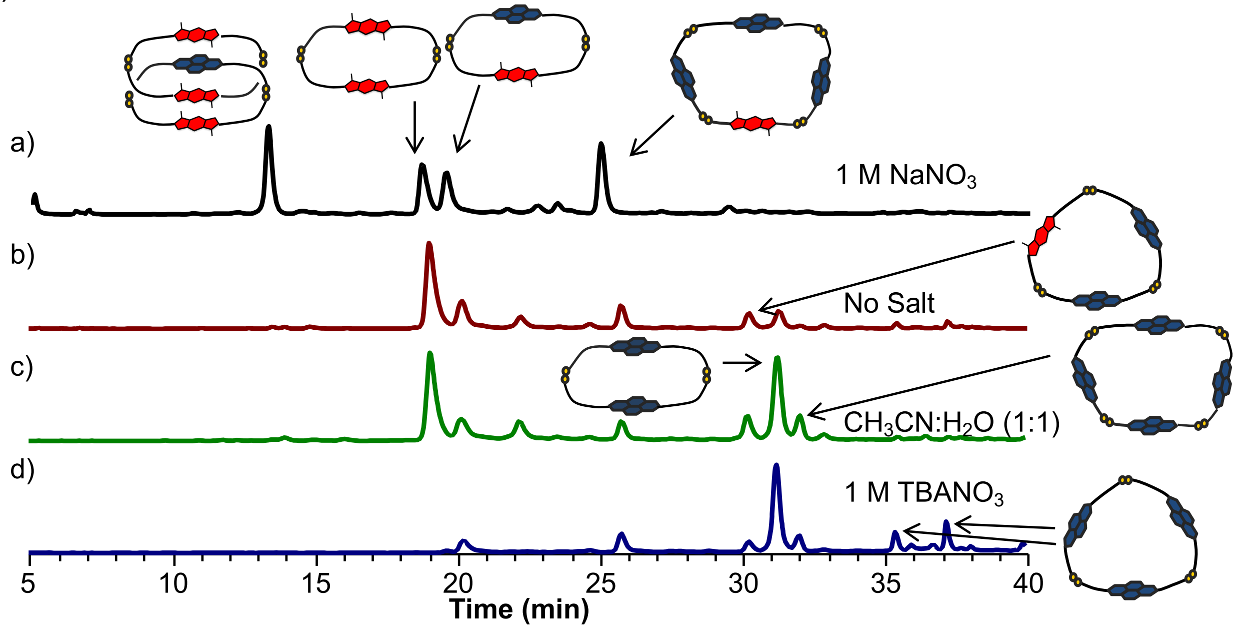

ii)

	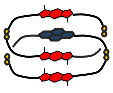	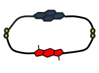	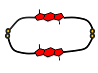	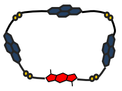	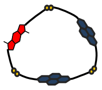	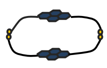	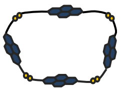	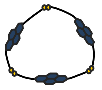	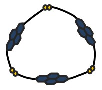
a)	21.26 (0.09)	29.41 (0.27)	26.11 (0.19)	18.17 (0.08)	2.89 (0.06)	2.15 (0.13)	-	-	-
b)	-	58.38 (0.43)	19.03 (0.32)	6.28 (0.02)	5.82 (0.12)	10.50 (0.18)	-	-	-
c)	-	39.34 (0.13)	11.81 (0.41)	3.62 (0.15)	6.64 (0.06)	33.20 (0.60)	4.64 (0.13)	0.75 (0.11)	-
d)	-	1.15 (0.06)	10.03 (0.09)	6.28 (0.09)	4.96 (0.09)	57.47 (0.38)	5.26 (0.38)	6.53 (0.26)	8.32 (0.02)

Supplementary Figure 36. i): Reverse-phase HPLC analysis of *S,S*-**1**:*R,R*-**2** (1:1 molar ratio, 5 mM total concentration) library a) in the presence of 1 M $NaNO_3$, b) no salt, c) in a mixture of CH_3CN:H_2O (1:1), d) in the presence of 1 M $TBANO_3$. Absorbances recorded at 389 nm. ii): The table shows the percentage of each species identified in the chromatograms above (i). The integrations were done in triplicate and the RMSD is reported in parenthesis along with the value. Each row of the table corresponds to the chromatogram bearing the same identification in the figure above. The unlabelled peaks did not ionise and could not be identified.

From these experiments, we can conclude that $K_{d2}>K_{d3}$ (from **Supplementary Figure 25**).

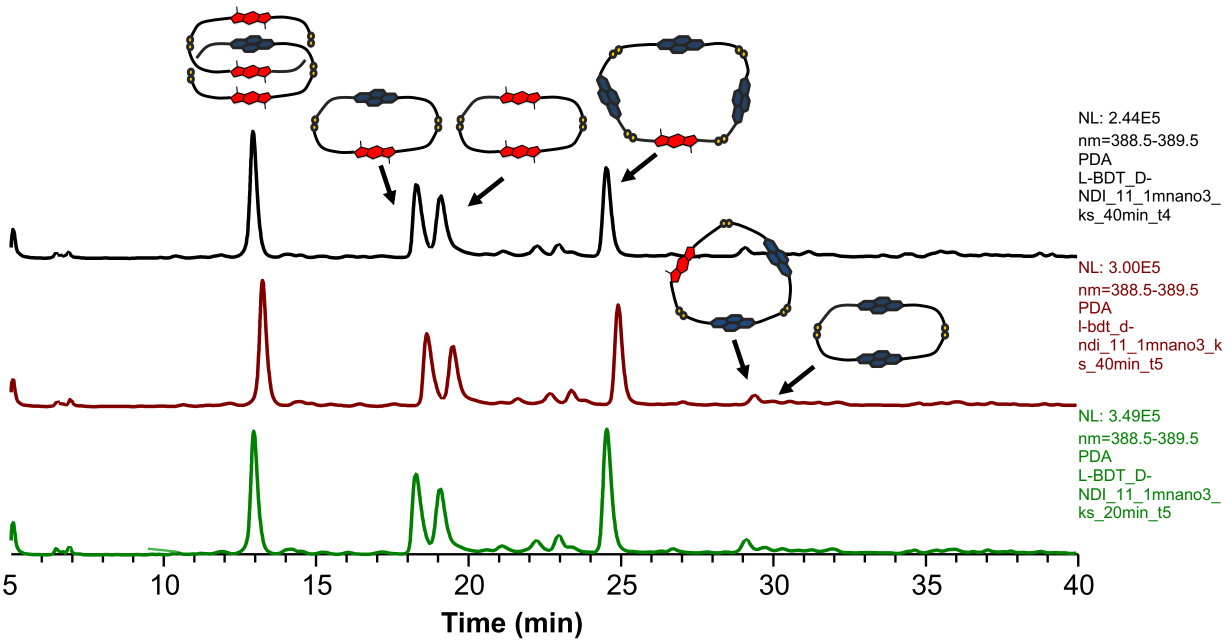

Supplementary Figure 37. Reverse-phase HPLC analysis of *S,S*-**1**:*R,R*-**2** (1:1 molar ratio, 5 mM total concentration) library in the presence of 1 M $NaNO_3$ of different libraries and/or the same library analysed at different times.

i)

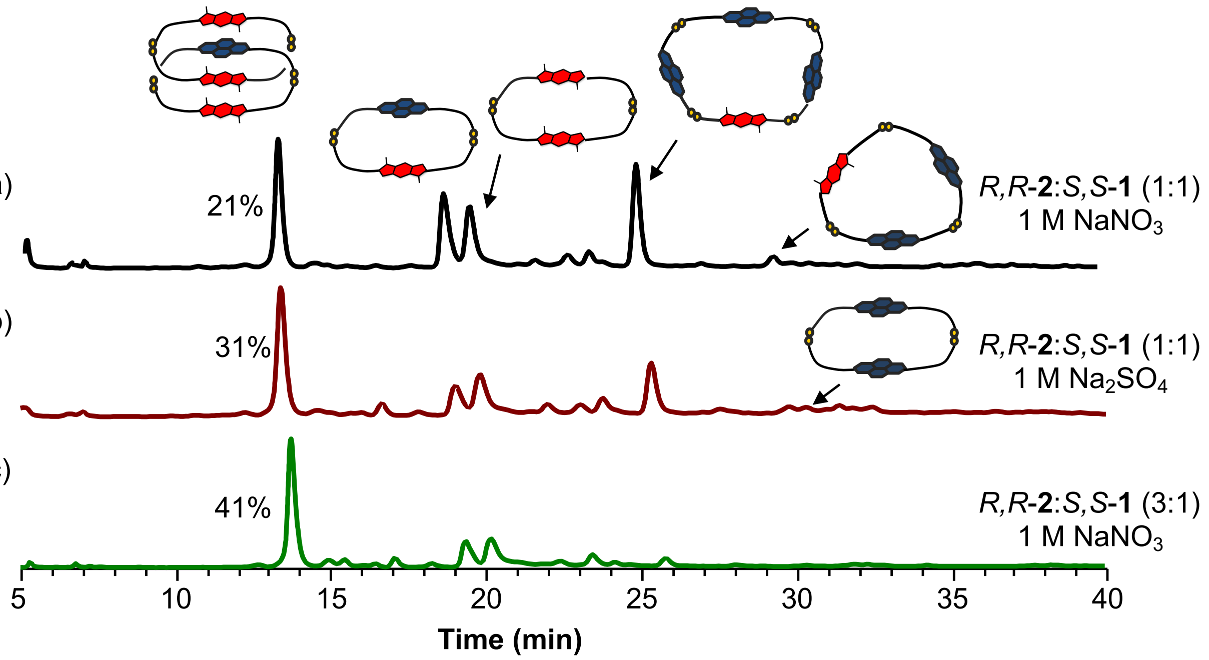

ii)

	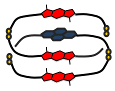	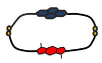	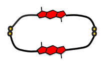	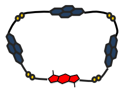	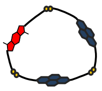	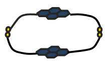
a)	21.26 (0.09)	29.41 (0.27)	26.11 (0.19)	18.17 (0.08)	2.89 (0.06)	2.15 (0.13)
b)	31.48 (0.00)	18.08 (0.17)	26.71 (0.32)	14.66 (0.17)	4.01 (0.22)	5.05 (0.41)
c)	40.61 (0.14)	22.07 (0.52)	34.17 (0.45)	3.15 (0.06)	-	-

Supplementary Figure 38. i) Reverse-phase HPLC analysis of S,S-**1**:R,R-**2** (1:1 molar ratio, 5 mM total concentration) library a) in the presence of 1 M $NaNO_3$, b) 1 M Na_2SO_4 and c) S,S-**1**:R,R-**2** (1:3 molar ratio, 5 mM total concentration) in the presence of 1 M $NaNO_3$. Absorbance spectra recorded at 389 nm. The yields of **Cat II RSRR** are shown on top of each chromatogram. ii) The table shows the percentage of each species identified in the chromatograms above (i). The integrations were done in triplicate and the RMSD is reported in parenthesis along with the value. Each row of the table corresponds to the chromatogram bearing the same identification in the figure above. The unlabelled peaks did not ionise and could not be identified.

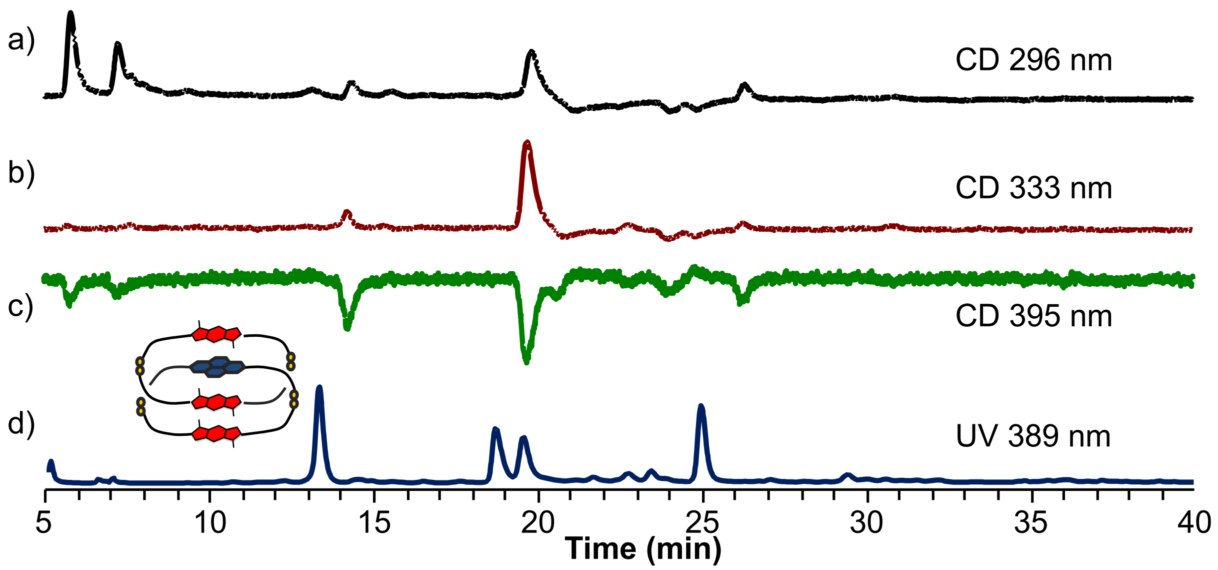

Supplementary Figure 39. Reverse-phase HPLC analysis of *S,S*-**1**:*R,R*-**2** (1:1 molar ratio, 5 mM total concentration) library in the presence of 1 M $NaNO_3$, showing a) CD at 296 nm, b) CD at 333 nm, c) CD at 395 nm and d) absorbance at 389 nm.

The large Cotton effect observed for the species with the retention time between 5 and 8 min is related to the overall low optical activity of the library members.

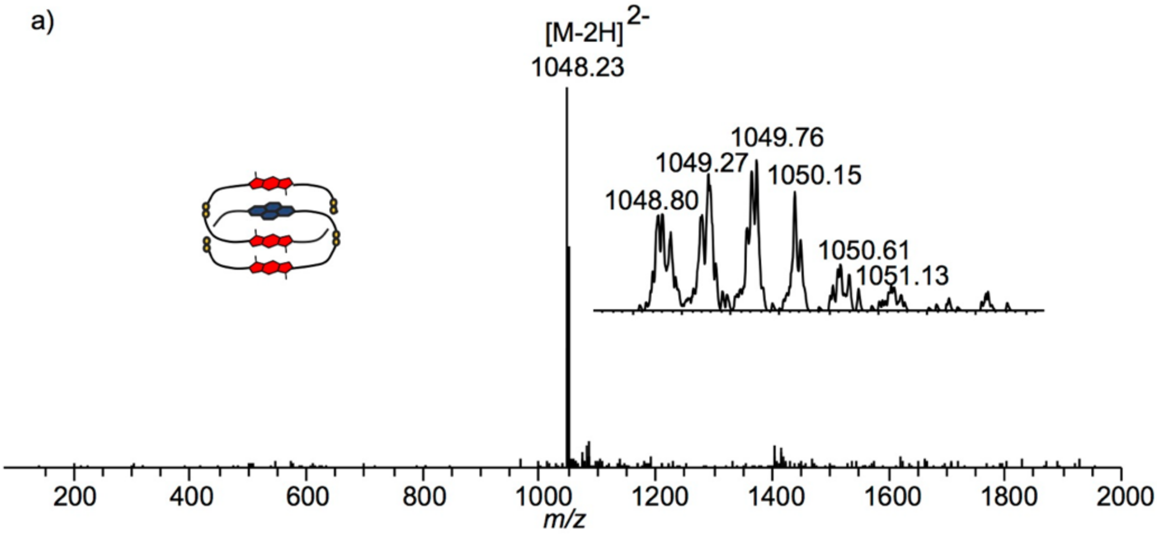

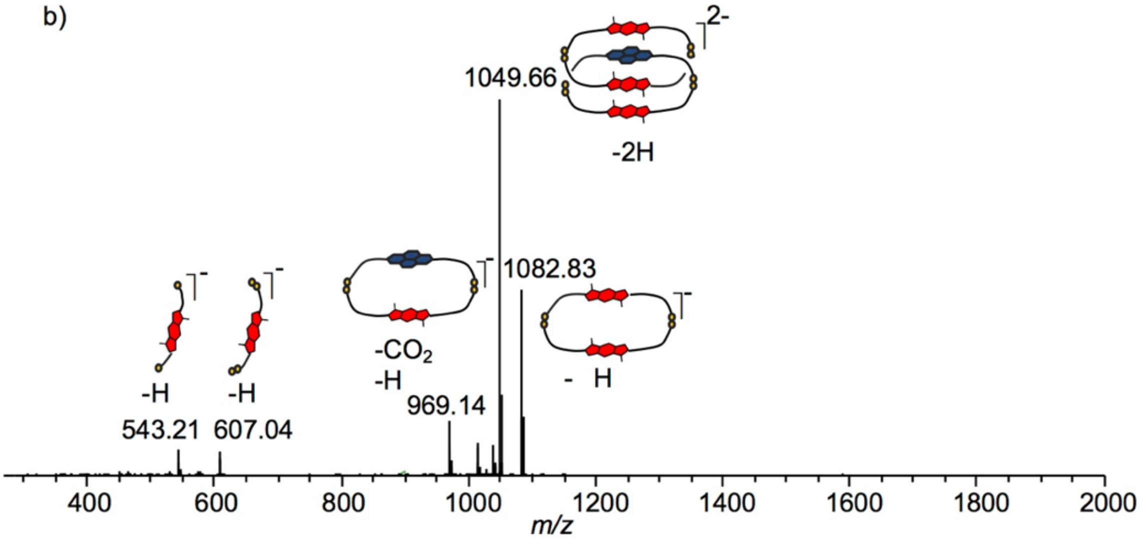

Supplementary Figure 40. a) MS (-*ve*) of **Cat II RSRR**; zoom of molecular ion is shown as inset. b) MS/MS (-*ve*) of **Cat II RSRR**.

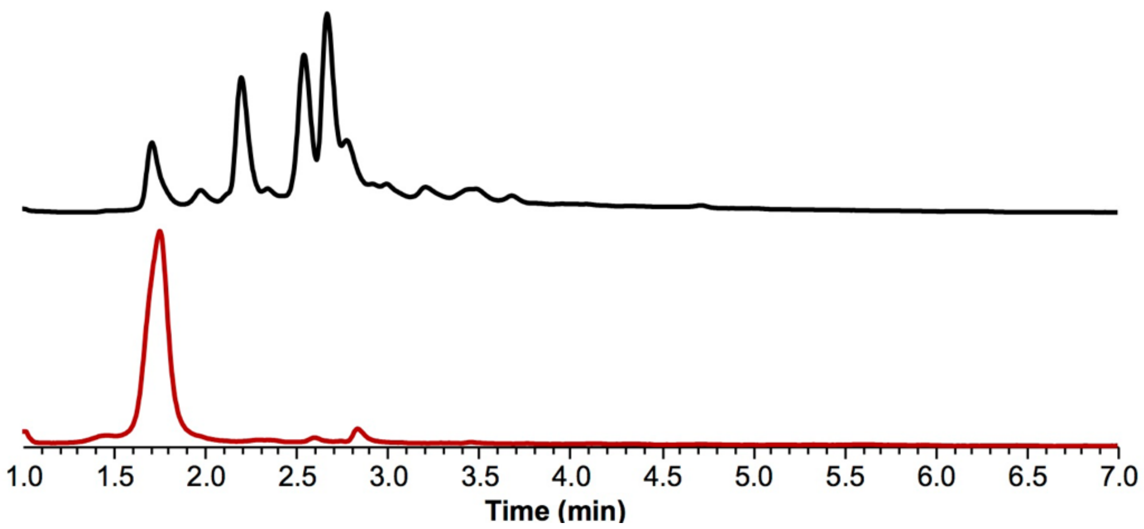

Supplementary Figure 41. Reverse-phase HPLC analysis of *S,S*-**1**:*R,R*-**2** (1:1 molar ratio, 5 mM total concentration) library (black trace) and **Cat II RSRR** after isolation (red trace). Absorbance spectra recorded at 389 nm.

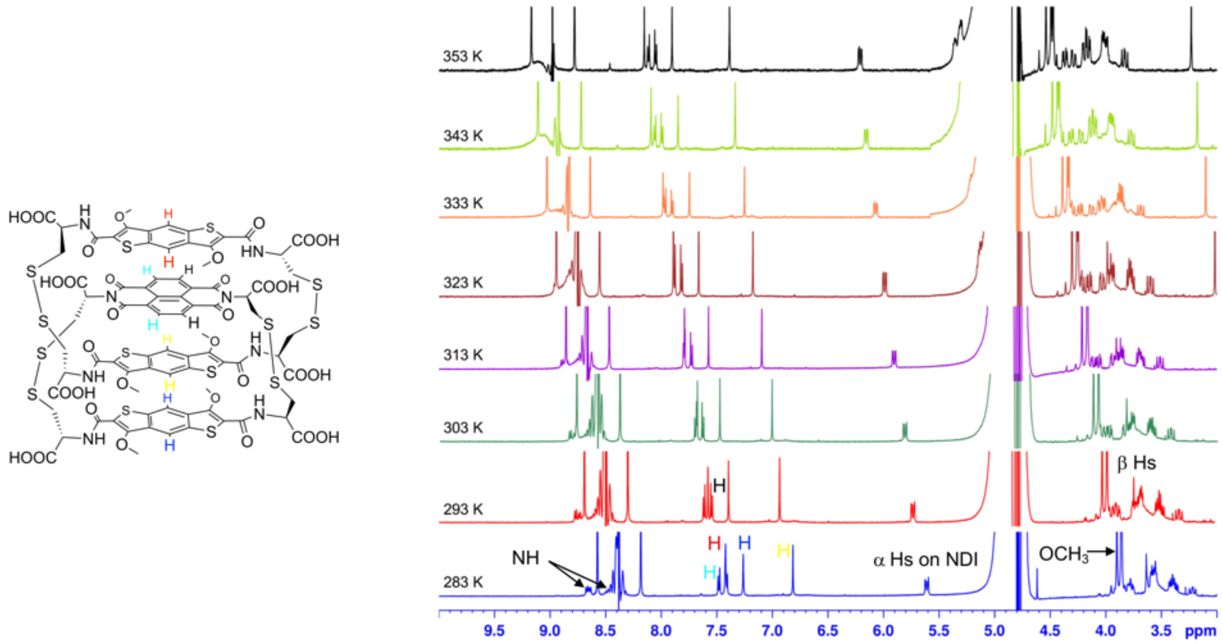

Supplementary Figure 42. Variable temperature ^{1}H NMR (500 MHz) of **Cat II RSRR** at the temperatures indicated above each spectrum. The solvent (H_2O) was referenced at 4.79 ppm.

The VT-NMR experiment is useful in confirming that the structure is a catenane. In the case of an isomeric macrocycle, the ^{1}H NMR peaks would get broader as the temperature increases due to high thermal energy. The sharpness of the peaks and the relative constant shift as the temperature increases is supportive of an interlocked geometry for this molecule.

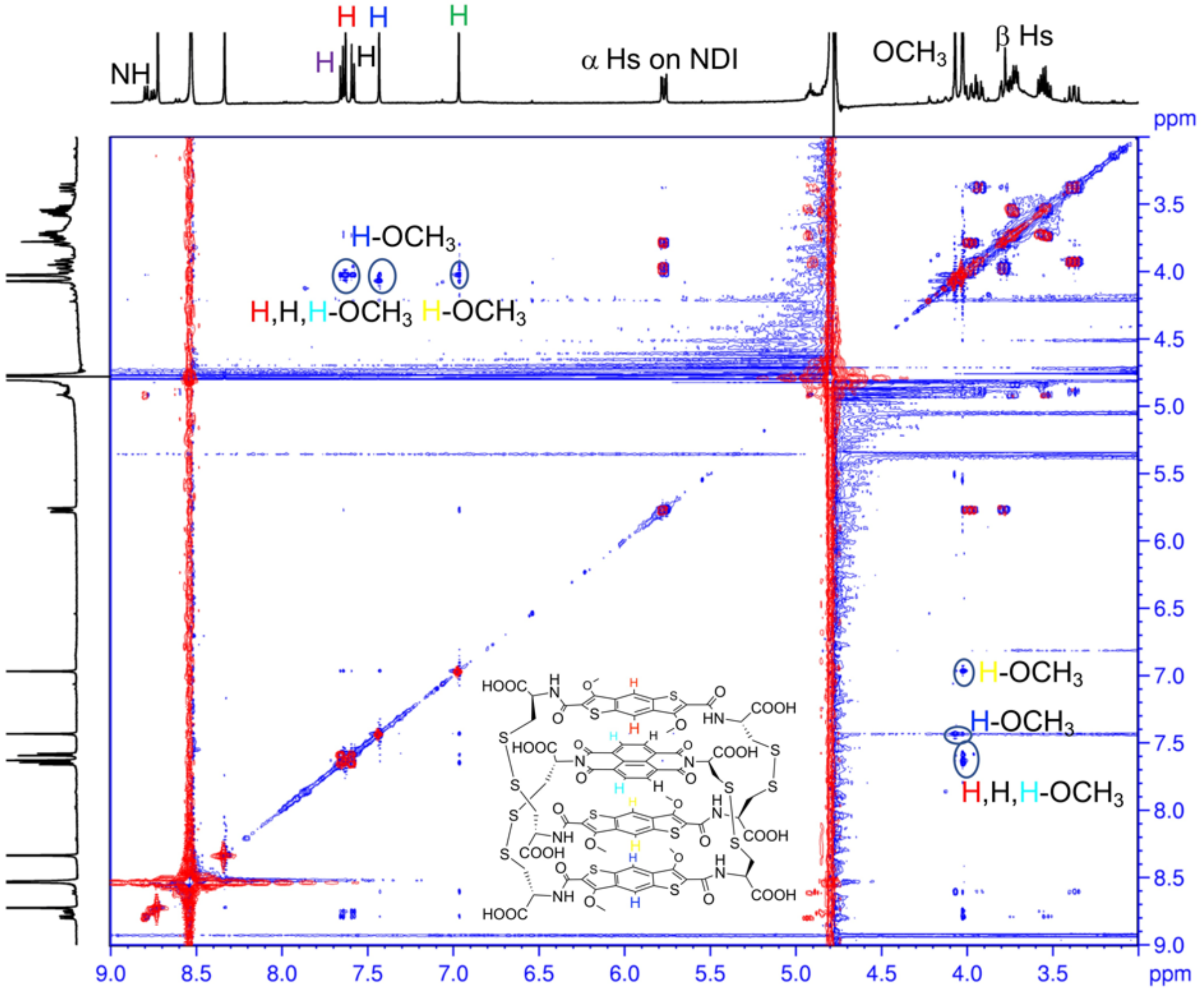

Supplementary Figure 43. ^{1}H-^{1}H COSY (red) and 2D NOESY (blue) spectra superimposed of **Cat II RSRR**. The solvent (H_2O) was referenced at 4.79 ppm.

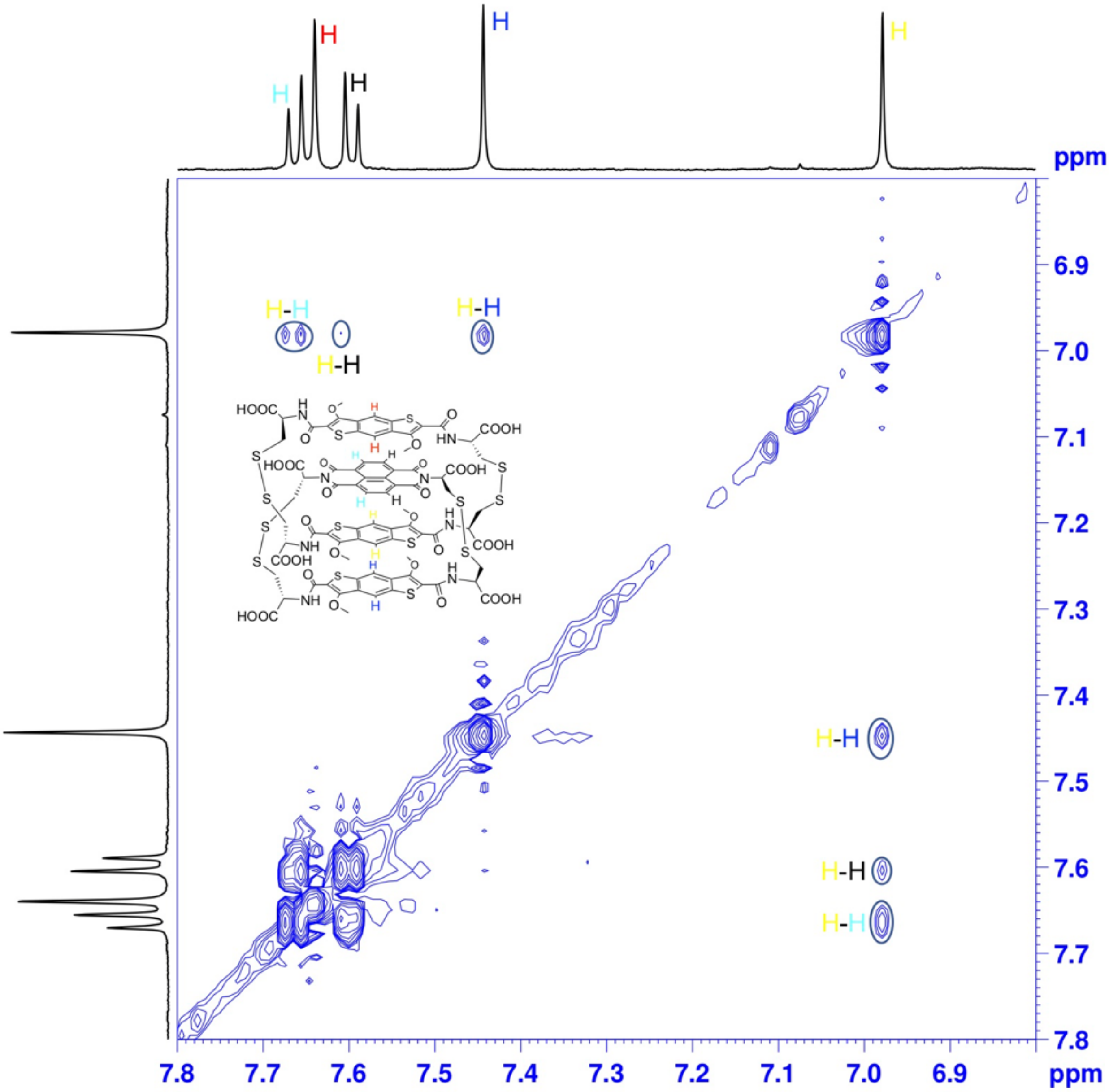

Supplementary Figure 44. 2D NOESY (blue) spectra superimposed of **Cat II RSRR**. The solvent (H_2O) was referenced at 4.79 ppm.

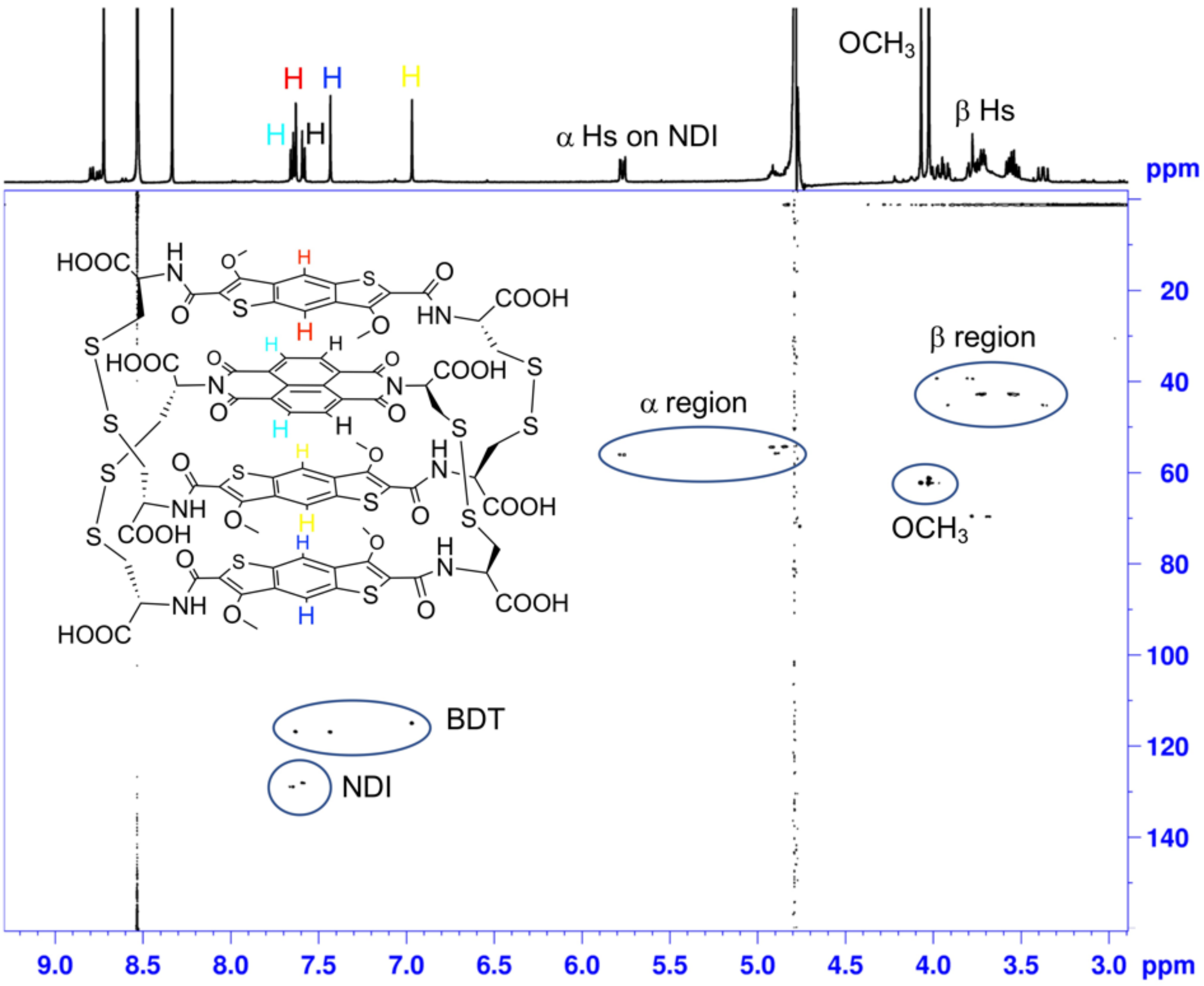

Supplementary Figure 45. HSQC spectrum (500 MHz, 298 K) of **Cat II RSRR**. The solvent (H_2O) was referenced at 4.79 ppm.

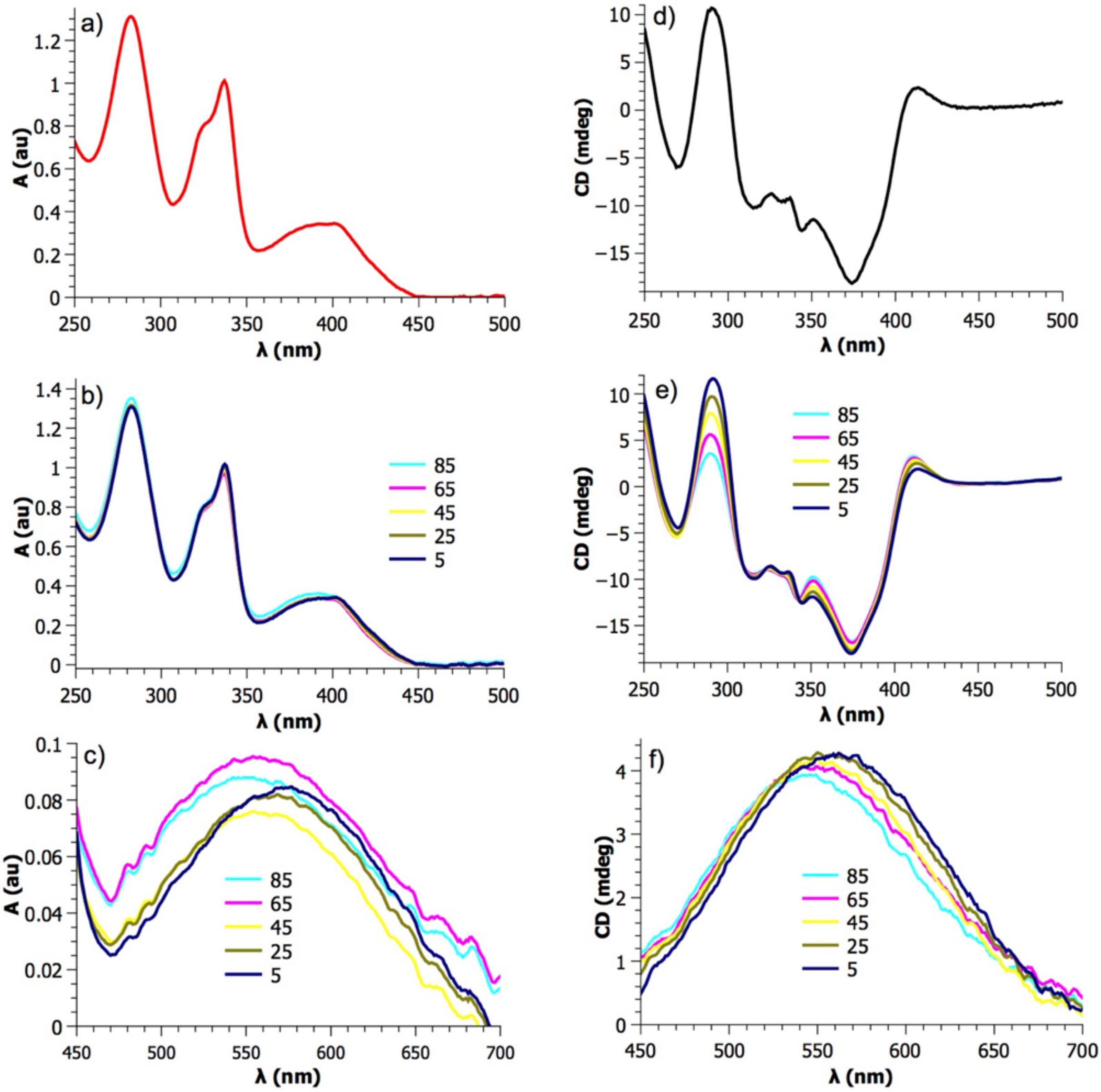

Supplementary Figure 46. a) UV-Vis spectrum of **Cat II RSRR** at 23 °C, b) VT UV-Vis spectra of **Cat II RSRR**, c) VT UV-Vis spectra of **Cat II RSRR** in the CT region between 5 – 85 °C at specified the temperatures, d) CD spectrum of **Cat II RSRR** at 23 °C; e) VT CD spectra of **Cat II RSRR**, f) VT CD spectra of **Cat II RSRR** in the CT region between 5 – 85 °C at the specified temperatures.

Cat II RSRR decomposes during the variable temperature UV / CD experiment. We suspect that the decomposition is caused by a combination of two factors: light and temperature (photo- and thermal degradation) as in the VT-NMR experiment there is no decomposition seen.

Analysis of DCLs containing *R,R*-1 and *R,R*-2

i)

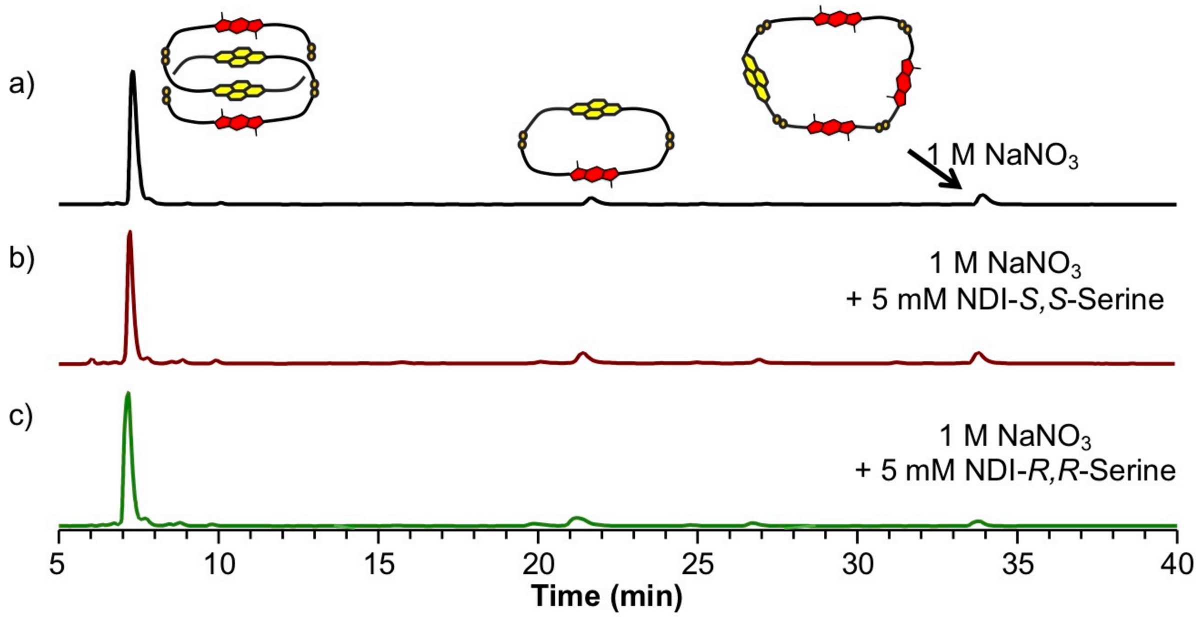

ii)

	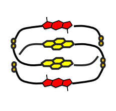	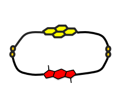	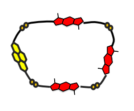
a)	75.83 (0.25)	16.38 (0.05)	7.79 (0.26)
b)	62.71 (0.58)	26.25 (0.90)	11.04 (0.50)
c)	76.42 (0.24)	19.62 (0.22)	3.95 (0.02

Supplementary Figure 47. i): Reverse-phase HPLC analysis of *R,R*-**1**:*R,R*-**2** (1:1 molar ratio, 5 mM total concentration) library a) in the presence of 1 M $NaNO_3$, b) 1 M $NaNO_3$ + 5 mM NDI-*S,S*-serine and c) 1 M $NaNO_3$ + 5 mM NDI-*R,R*-serine. Absorbance recorded at 389 nm. ii): The table shows the percentage of each species identified in the chromatograms above (i). The integrations were done in triplicate and the RMSD is reported in parenthesis along with the value. Each row of the table corresponds to the chromatogram bearing the same identification in the figure above. The unlabelled peaks did not ionise and could not be identified.

From these experiments, we can conclude that $K_{a2}>K_{a3}$ (from **Supplementary Figure 25**).

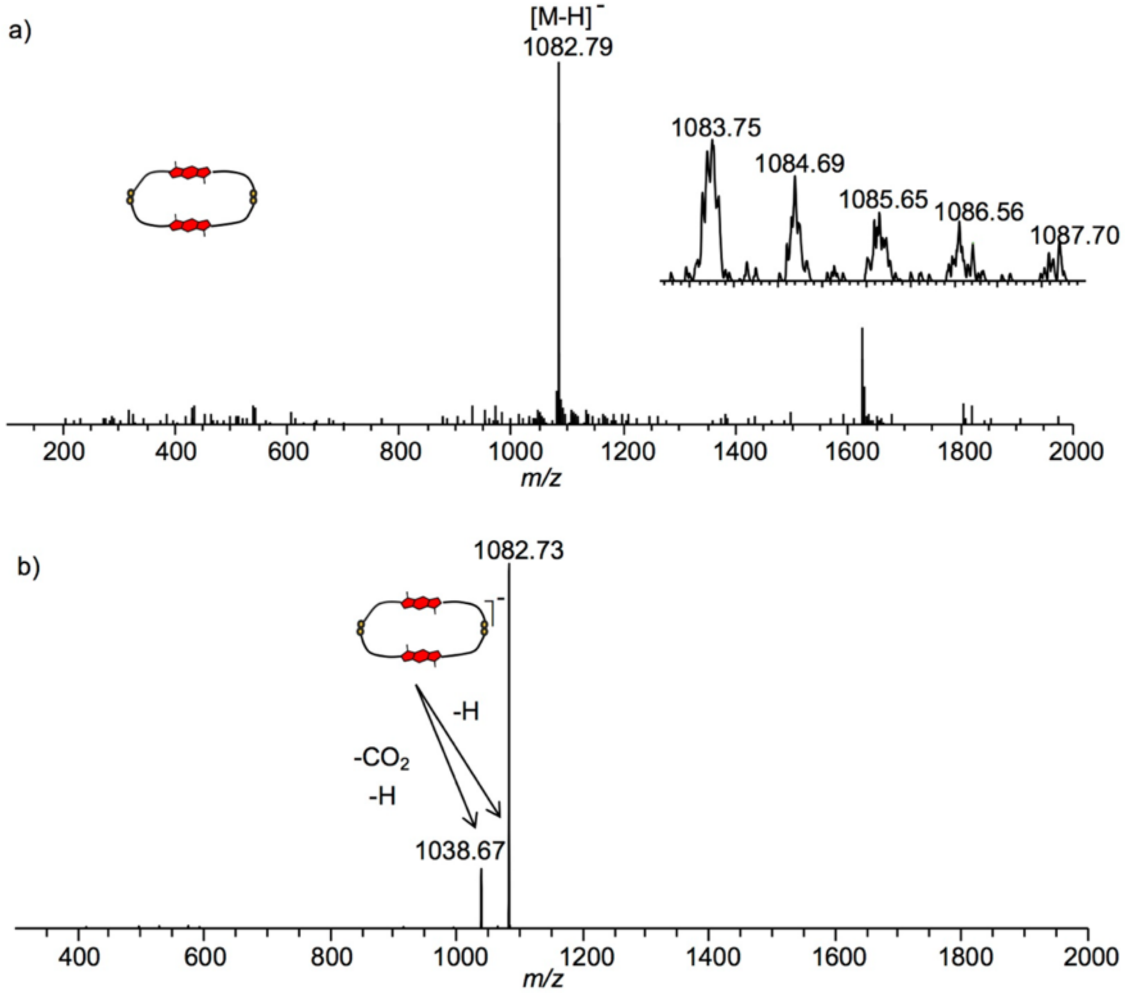

Supplementary Figure 48. a) MS (-*ve*) of **Y**; zoom of molecular ion is shown as inset. b) MS/MS (-*ve*) of **Y**.

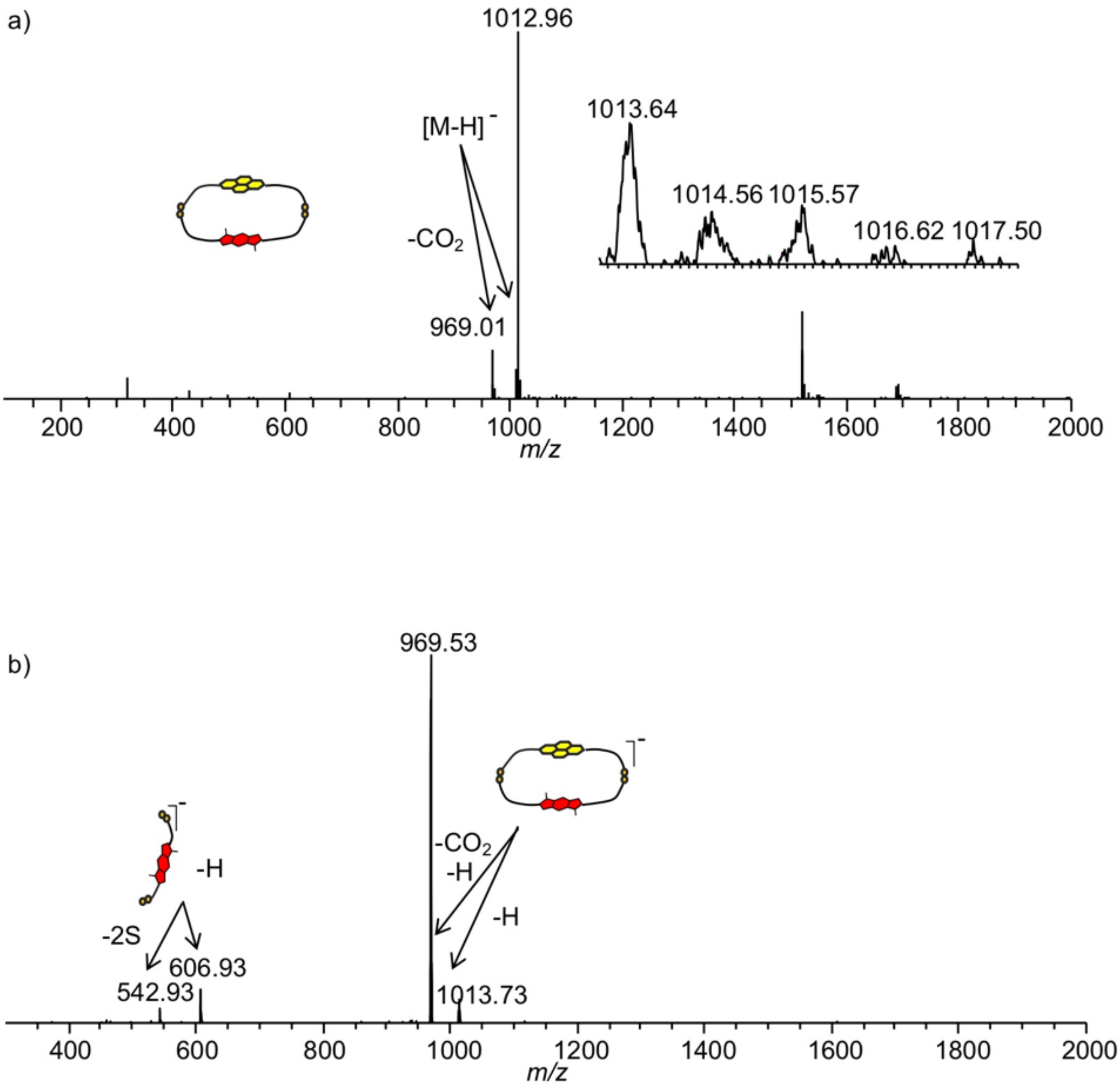

Supplementary Figure 49. a) MS (-*ve*) of **X**; zoom of molecular ion is shown as inset. b) MS/MS (-*ve*) of **X**.

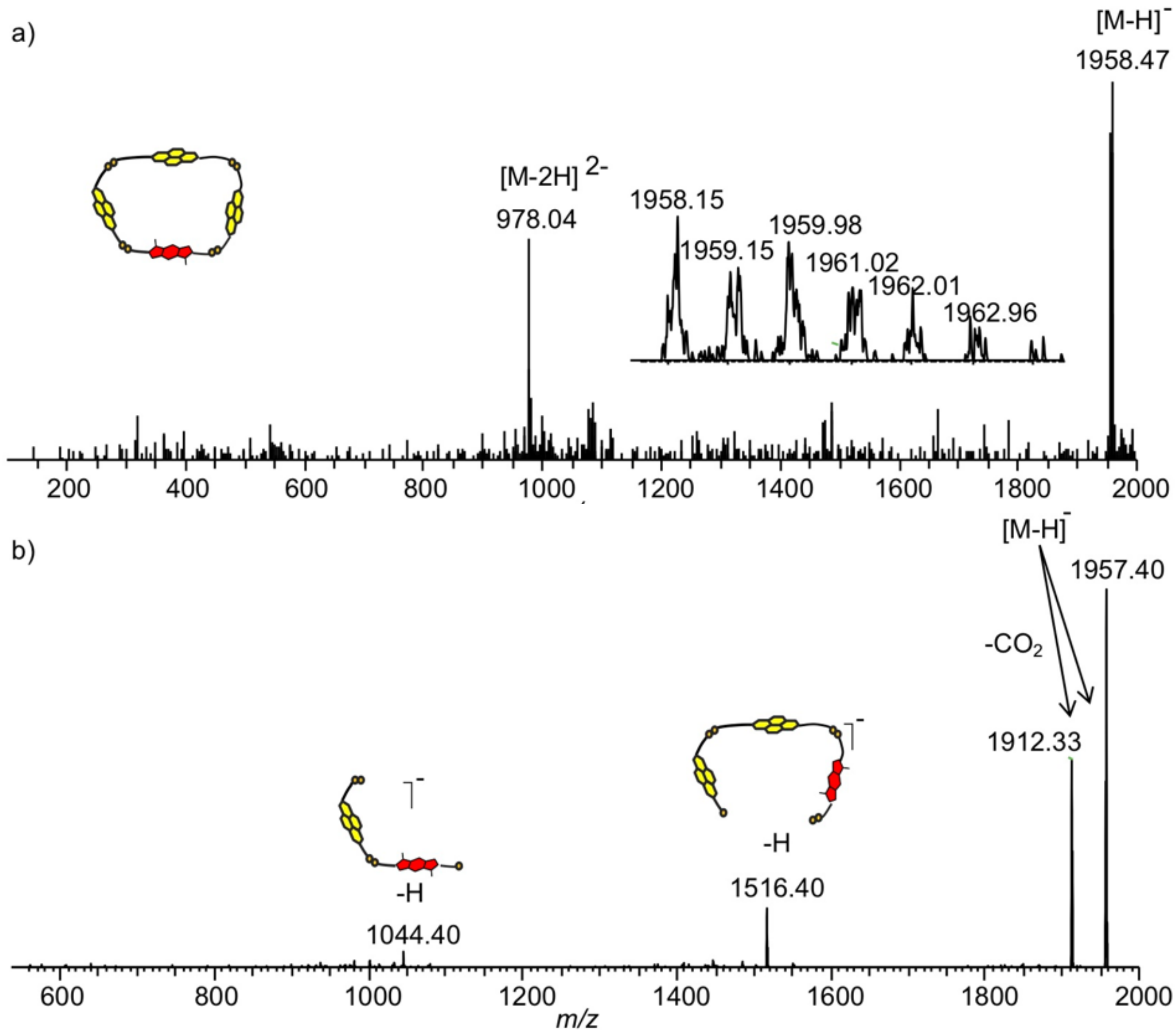

Supplementary Figure 50. a) MS (-*ve*) of tetramer based on three *R,R*-**1** and one *R,R*-**2**; zoom of molecular ion is shown as inset. b) MS/MS (-*ve*) of tetramer.

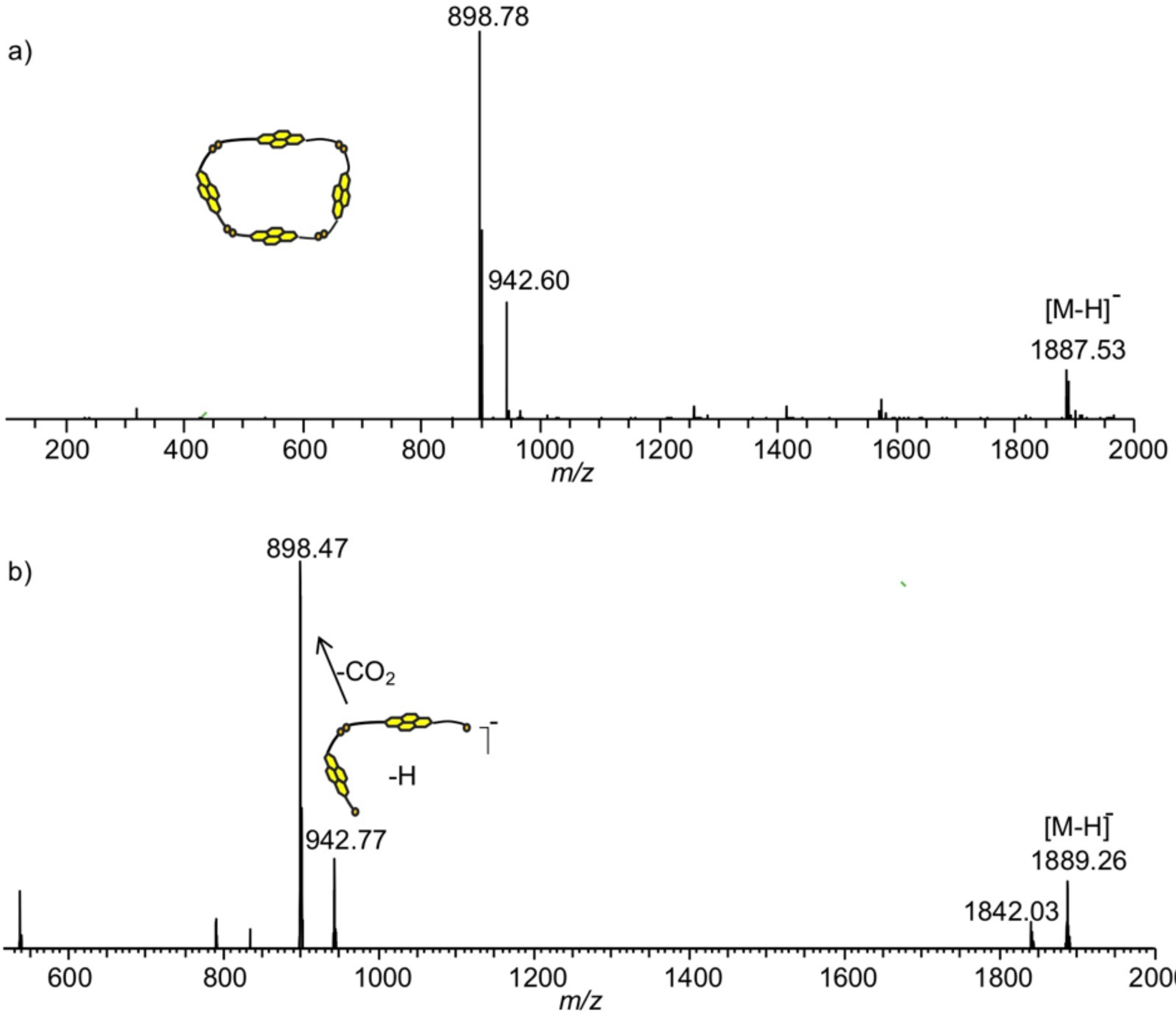

Supplementary Figure 51. a) MS (-*ve*) of *R,R*-**1** tetramer; zoom of molecular ion is shown as inset. b) MS/MS (-*ve*) of tetramer.

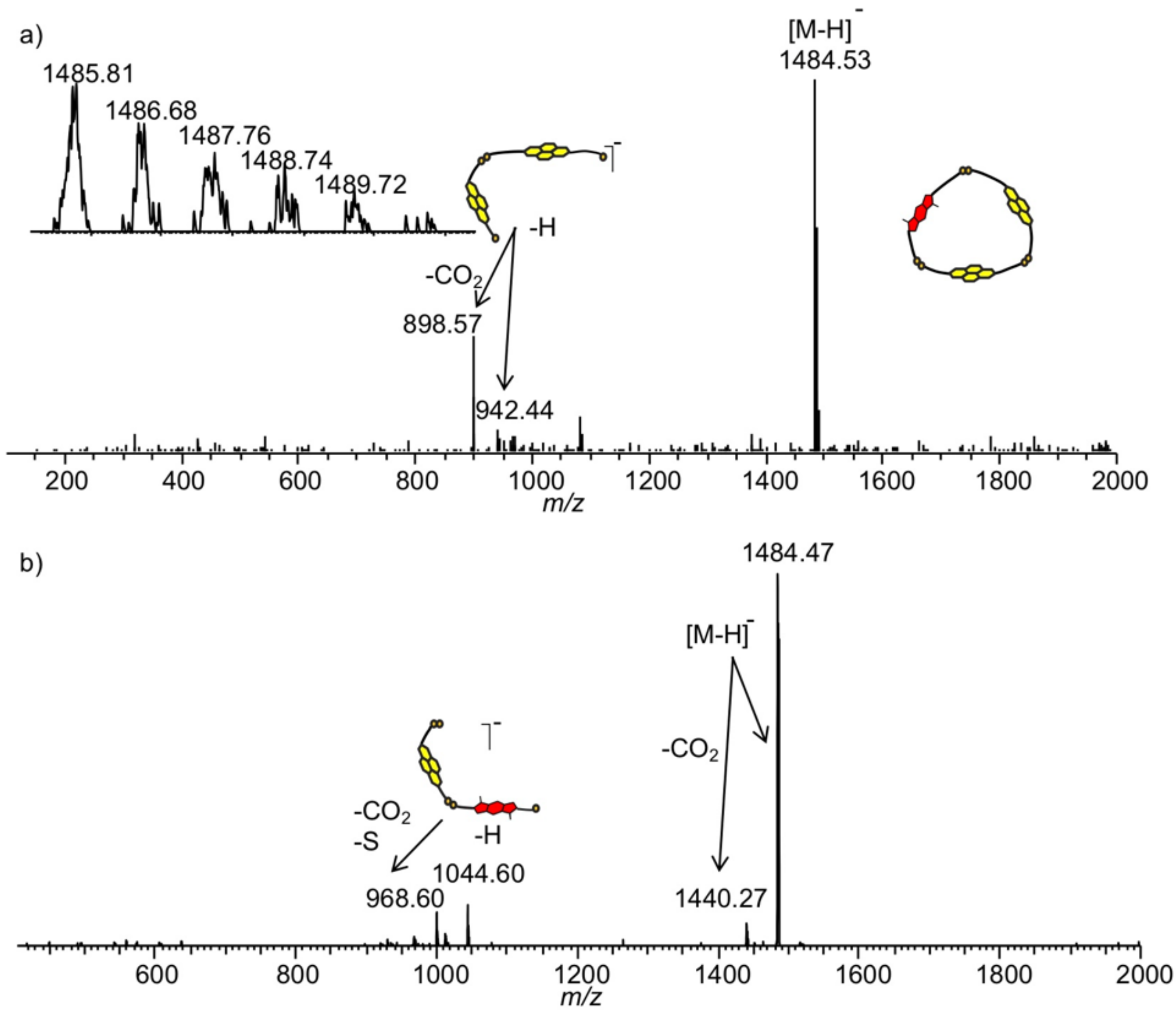

Supplementary Figure 52. a) MS (-*ve*) of heterotrimer based on two *R,R*-**1** units and one *R,R*-**2** unit; zoom of molecular ion is shown as inset. b) MS/MS (-*ve*) of heterotrimer.

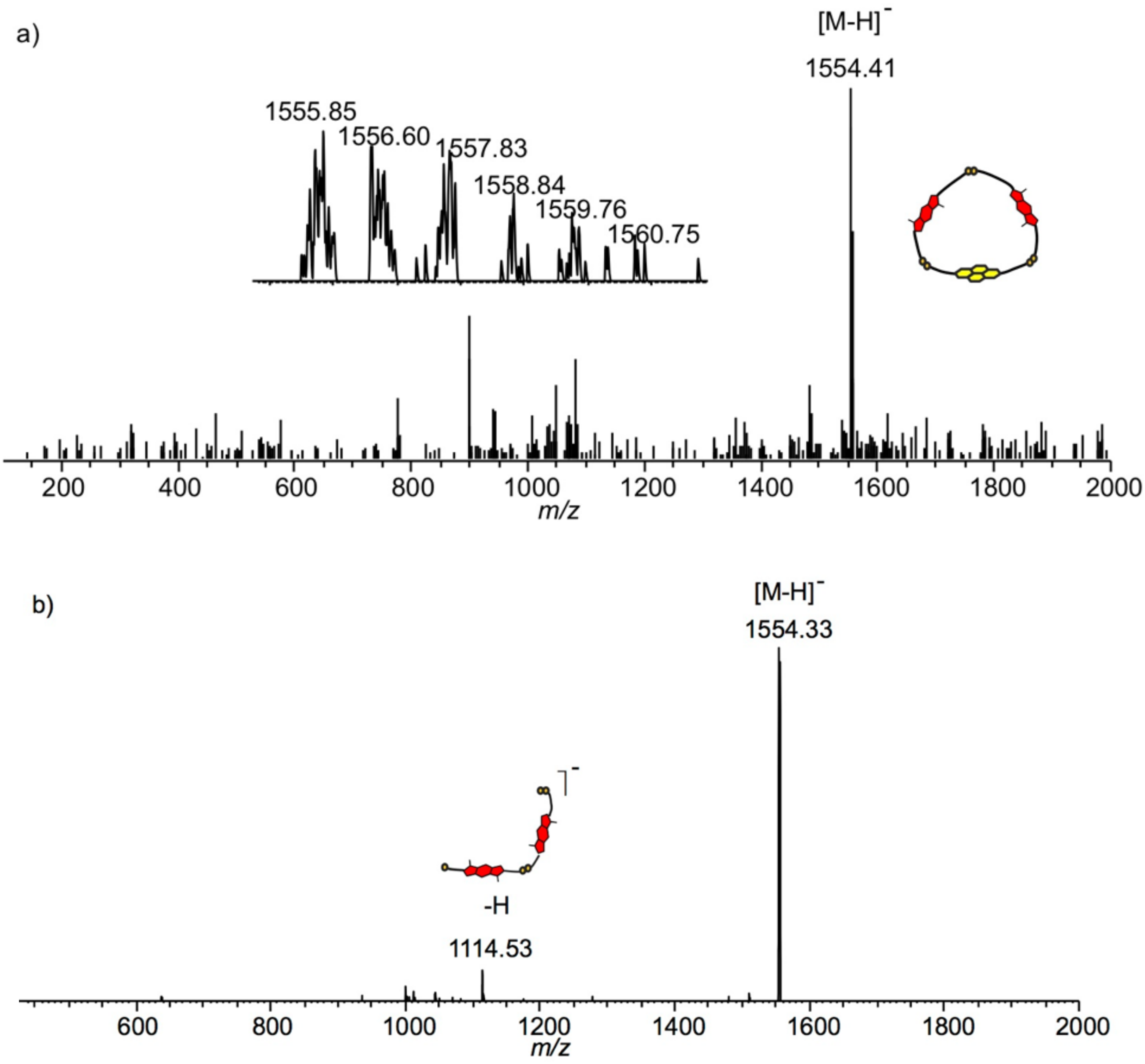

Supplementary Figure 53. a) MS (-*ve*) of heterotrimer based on one *R,R*-**1** unit and two *R,R*-**2** unit; zoom of molecular ion is shown as inset. b) MS/MS (-*ve*) of heterotrimer.

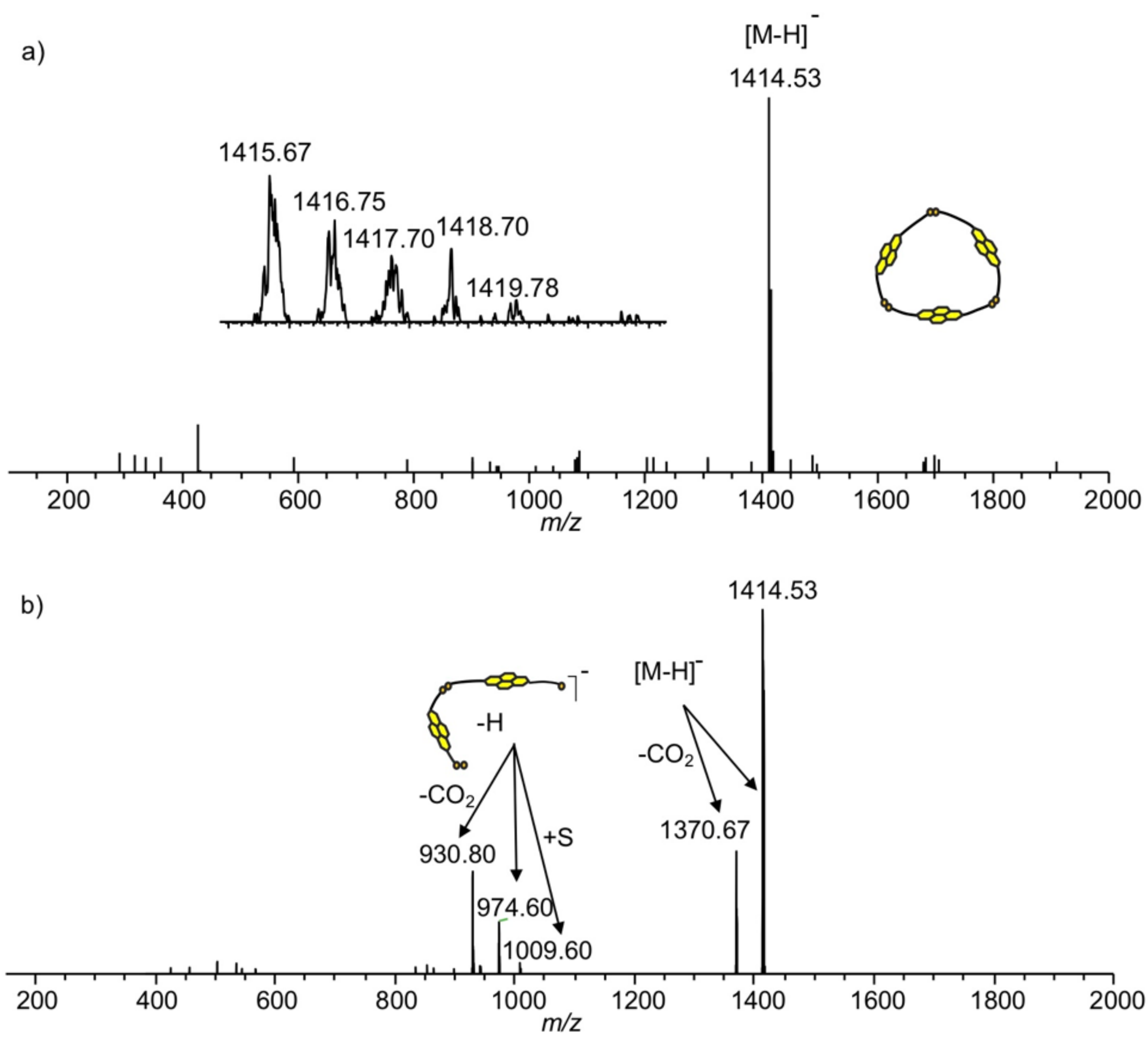

Supplementary Figure 54. a) MS (-*ve*) of *R,R*-**1** homotrimer; zoom of molecular ion is shown as inset. b) MS/MS (-*ve*) of homotrimer.

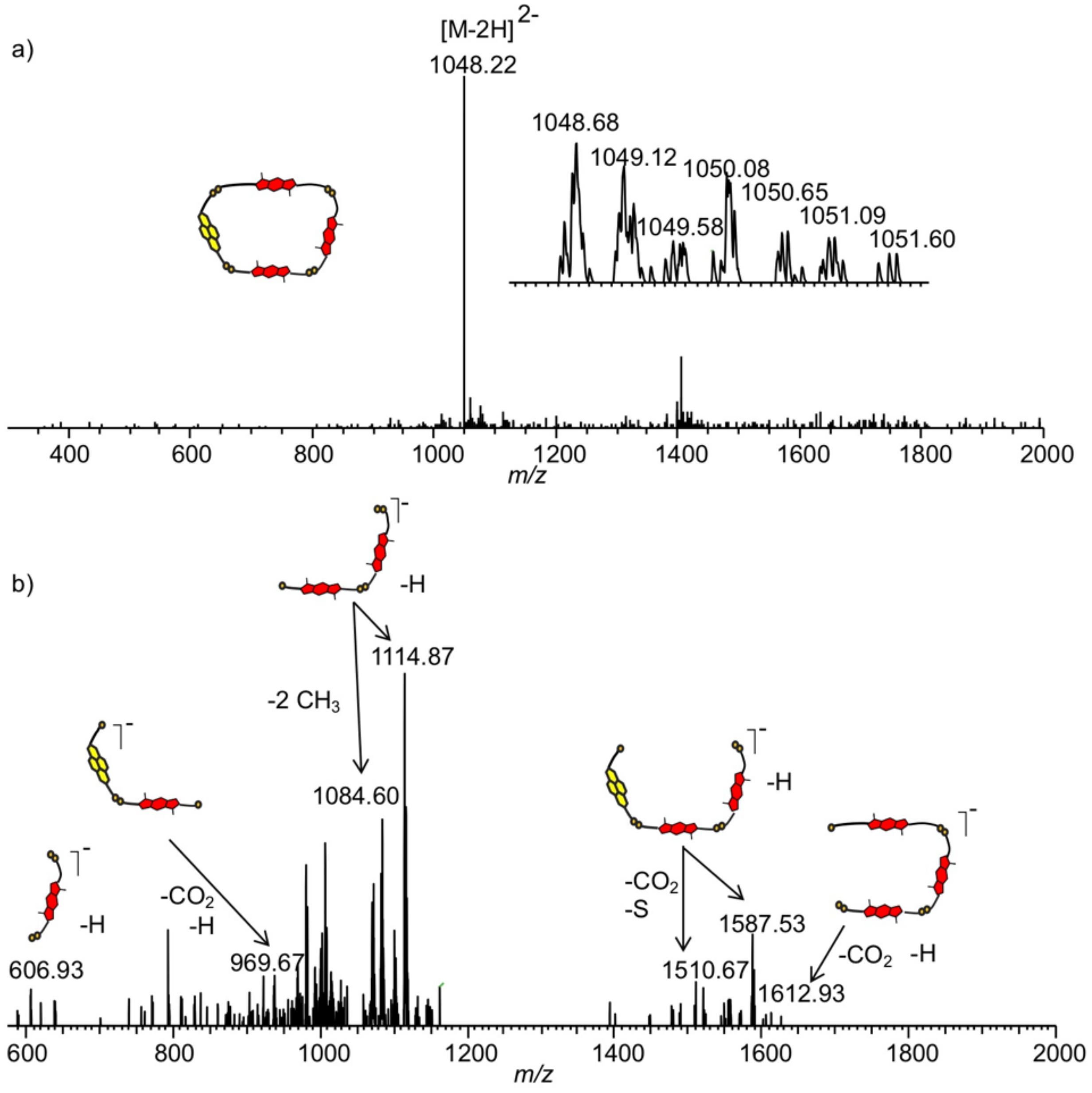

Supplementary Figure 55. a) MS (-*ve*) of heterotetramer based on one *R,R*-**1** unit and three *R,R*-**2** unit; zoom of molecular ion is shown as inset. b) MS/MS (-*ve*) of heterotetramer.

i)

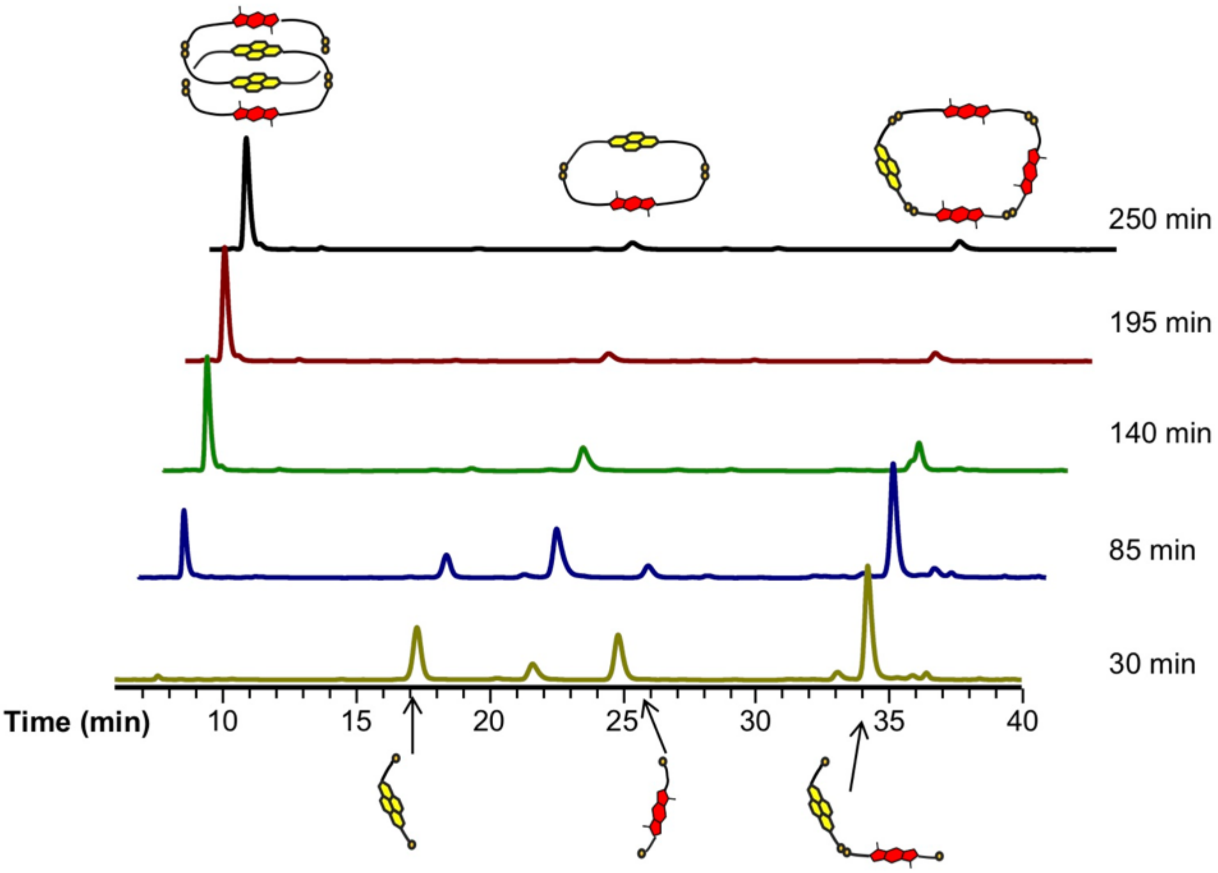

ii)

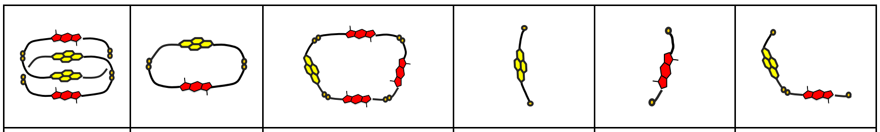

**Cat I RRRR**	**X**	*DDDA* (RRRR) Macrocycle	*R,R*-**1**	*R,R*-**2**	Linear **X**

Supplementary Figure 56. i) Reverse-phase HPLC analysis of *R,R*-**1**:*R,R*-**2** (1:1 molar ratio, 5 mM total concentration) library over time in the presence of 1 M $NaNO_3$. Absorbances recorded at 389 nm at different intervals: 305 min, 250 min, 195 min, 140 min, 85 min, 30 min as shown in figure. ii) The table describes the annotation corresponding to each species.

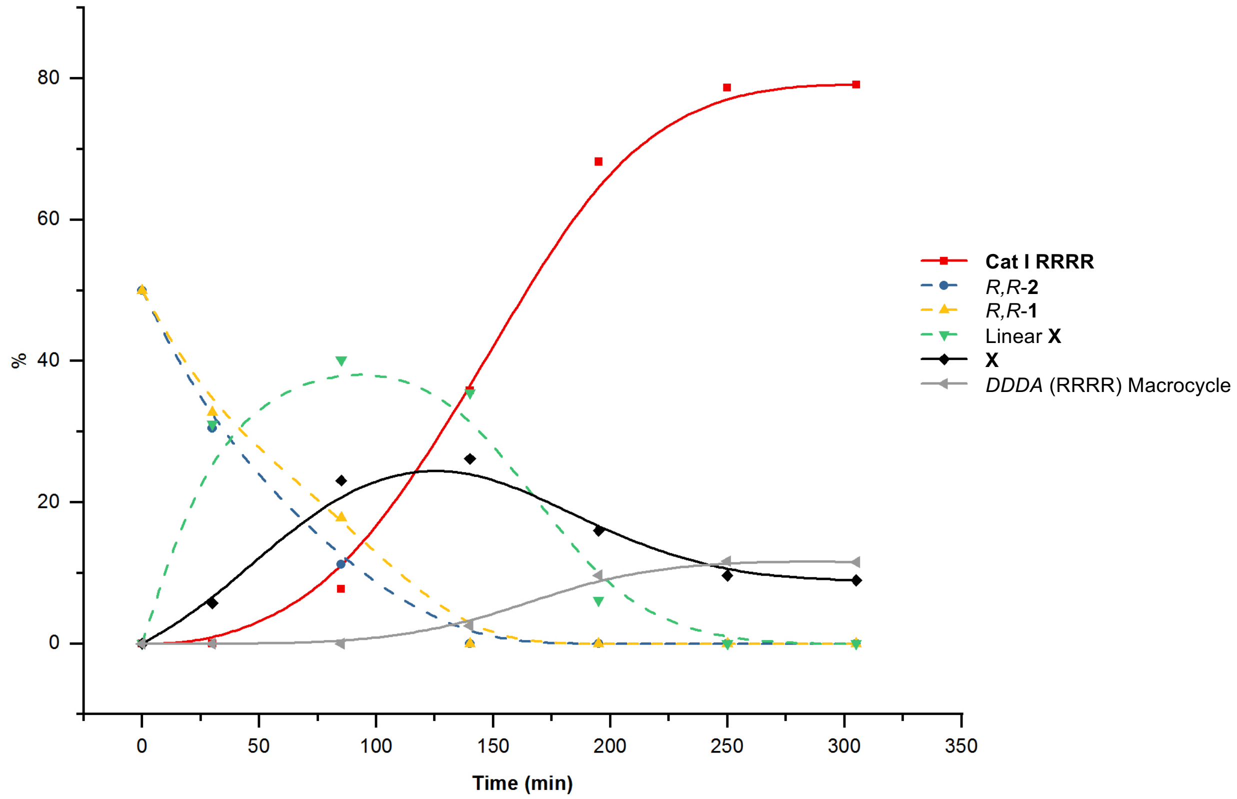

Supplementary Figure 57. Kinetic profile of *R,R*-**1**:*R,R*-**2** (1:1, 5 mM total concentration, absorption monitored at 389 nm) showing the consumption of the building blocks (dashed grey and orange lines, respectively) and the formation of the **Cat I RRRR** (red line). The formation and disappearance of other species can be also tracked: linear **X** (dashed green), **X** (black) and *DDDA* (RRRR) Macrocycle (grey). The dashed lines represent the intermediate species, while the solid lines the final products. *D* stands for donor (BDT) and *A* for acceptor (NDI). For clarity, spline curves were added; these do not represent mathematical models for kinetic data.

To a solution of **Y** (2.5 mM), *R,R*-**1** was added (as solid) to make the total DCL concentration 5 mM. After the pH was adjusted to 8 – 8.5, an HPLC chromatogram was recorded every 55 min. The experiment shows that the DCL is under thermodynamic control with the formation of **Cat I RRRR** as the major species. This library resembles with the one formed from *R,R*-**1** and *R,R*-**2** in 1:1 ratio.

i)

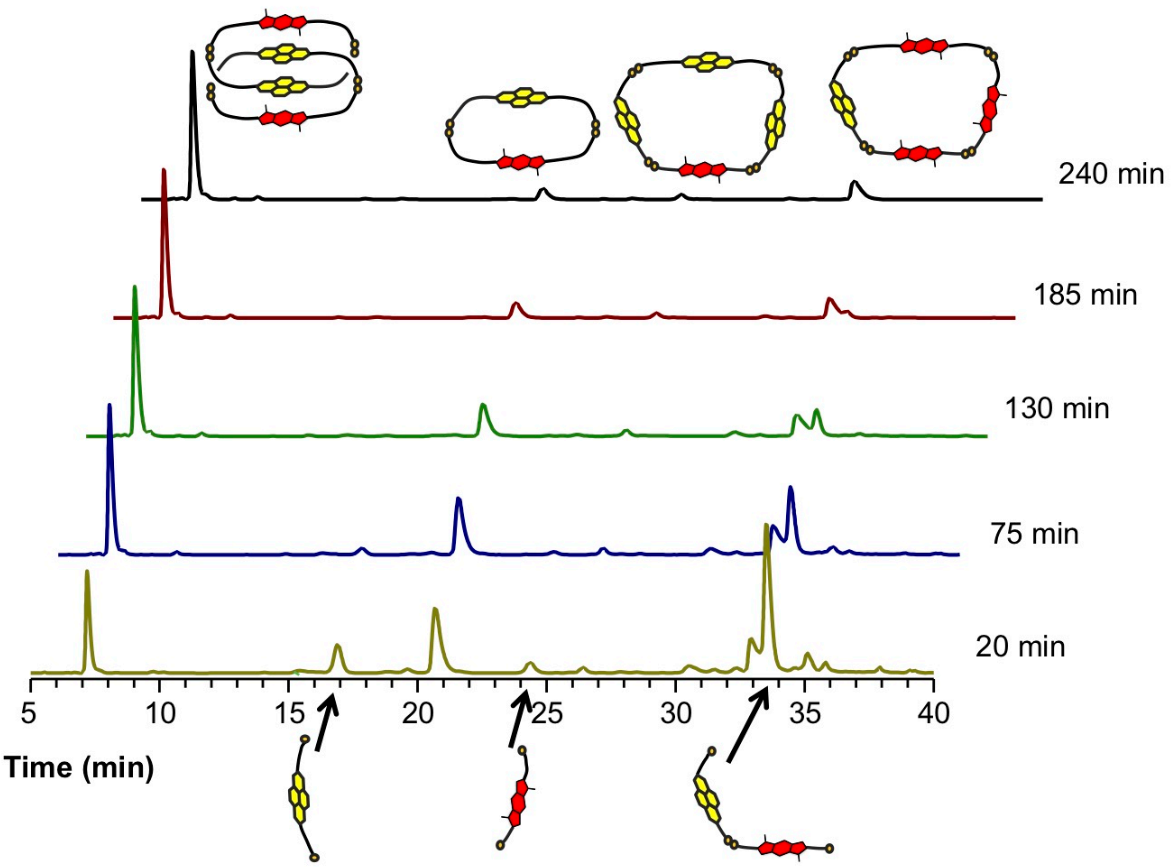

ii)

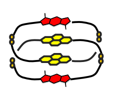	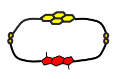	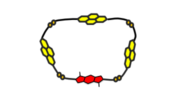	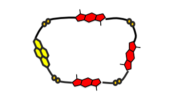	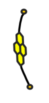	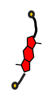	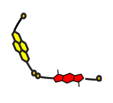
**Cat I RRRR**	**X**	*DAAA* (RRRR) Macrocycle	*DDDA* (RRRR) Macrocycle	*R,R*-**1**	*R,R*-**2**	Linear **X**

Supplementary Figure 58. i): Kinetic profile of *R,R*-**1**:**Y** over time in the presence of 1 M $NaNO_3$. Absorbances recorded at 389 nm at different intervals: 240 min, 185 min, 130 min, 75 min, 20 min as shown in figure. ii): The table describes the annotation corresponding to each species.

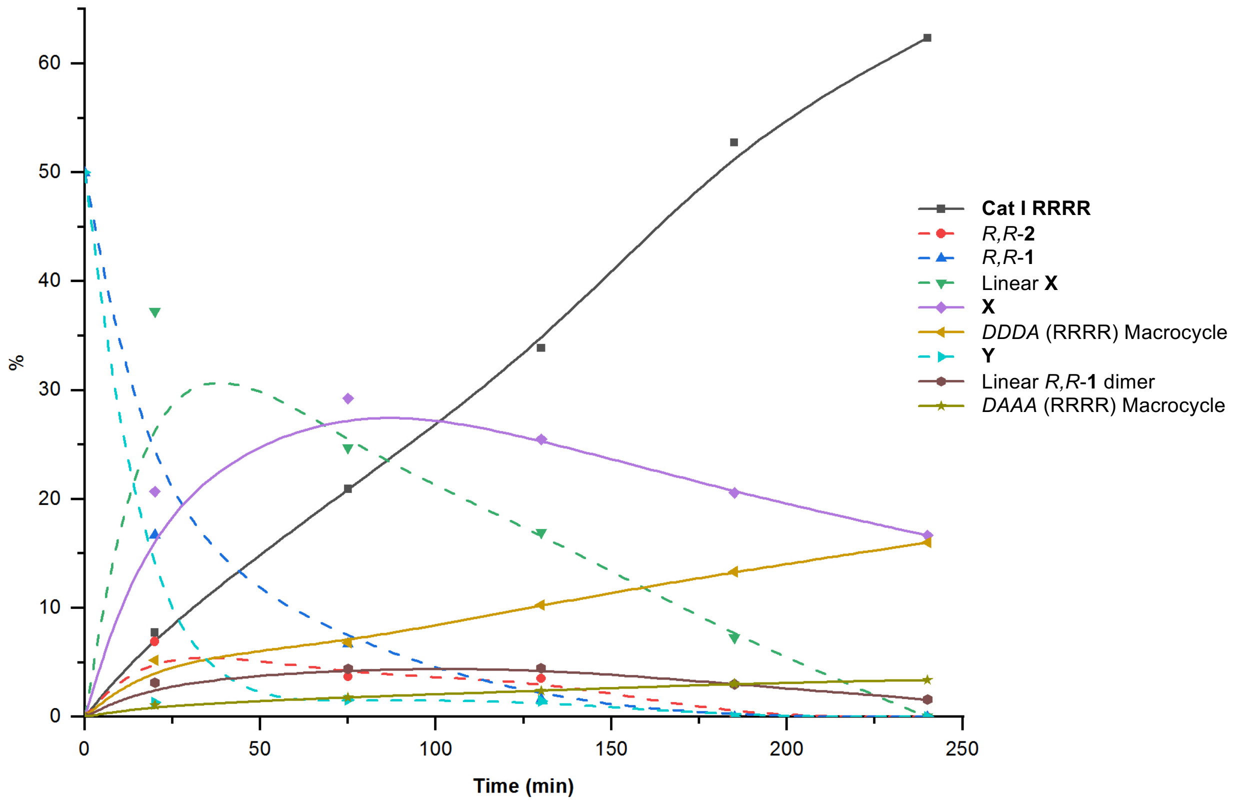

Supplementary Figure 59. Kinetic profile of *R,R*-**1**:**Y** (absorption monitored at 389 nm) showing the formation of the **Cat I RRRR** (black). The description of each species can be seen in legend (*D* stands for donor (BDT) and *A* stands for acceptor (NDI)). For clarity, spline curves were added; these do not represent mathematical models for kinetic data.

Analysis of DCLs containing *S,S*-1 and *R,R*-2

i)

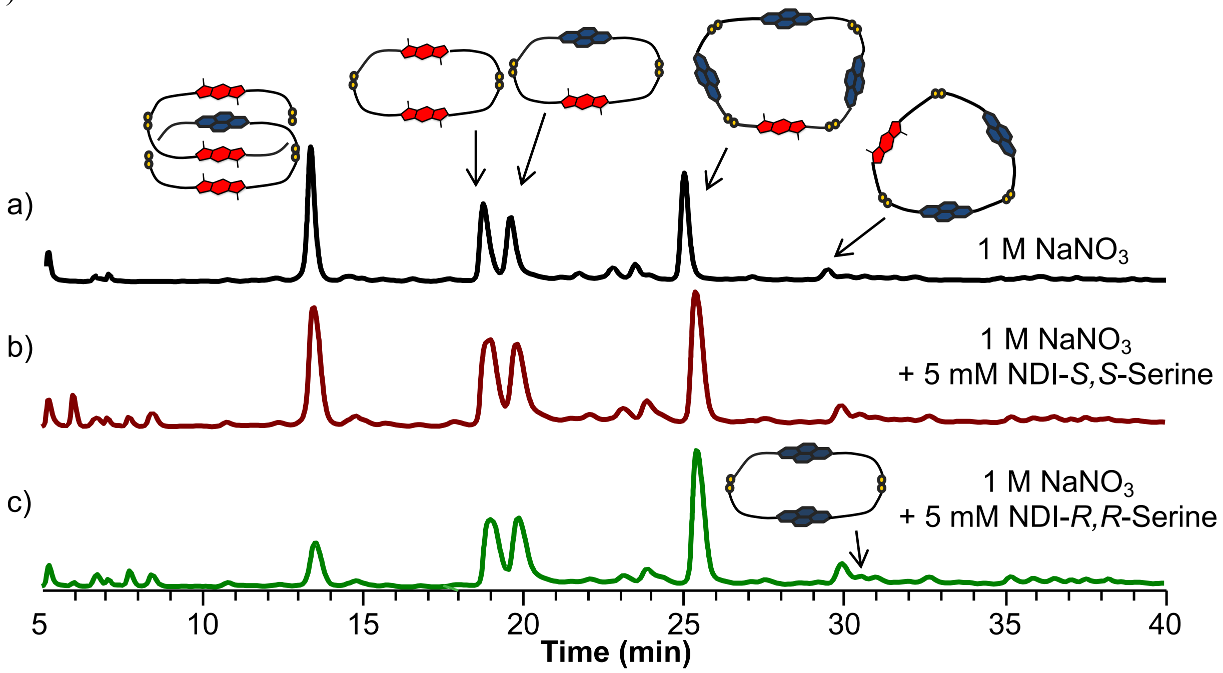

ii)

	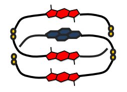	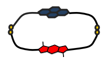	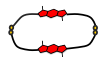	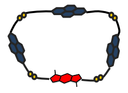	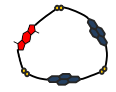	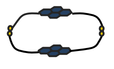
a)	21.26 (0.09)	29.41 (0.27)	26.11 (0.19)	18.17 (0.08)	2.89 (0.06)	2.15 (0.13)
b)	15.91 (0.05)	30.80 (0.11)	27.76 (0.09)	18.67 (0.19)	3.87 (0.13)	2.97 (0.36)
c)	7.76 (0.12)	30.28 (0.04)	29.24 (0.49)	24.76 (0.19)	5.35 (0.19)	2.61 (0.02)

Supplementary Figure 60. i) Reverse-phase HPLC analysis of *S,S*-**1**:*R,R*-**2** (1:1 molar ratio, 5 mM total concentration) library a) in the presence of 1 M $NaNO_3$, b) 1 M $NaNO_3$ + 5 mM **NDI-*S,S*-serine** and c) 1 M $NaNO_3$ + 5 mM **NDI-*R,R*-serine**. Absorbances recorded at 389 nm. ii) The values in the table represent the percentage of each species identified in the chromatograms above (i). The integrations were done in triplicate and the RMSD is reported in parenthesis along with the value. Each row of the table corresponds to the chromatogram bearing the same identification in the figure above. The unlabelled peaks did not ionise and could not be identified.

From these experiments, we can conclude that $K_{a6}>K_{a5}$ (from **Supplementary Figure 25**).

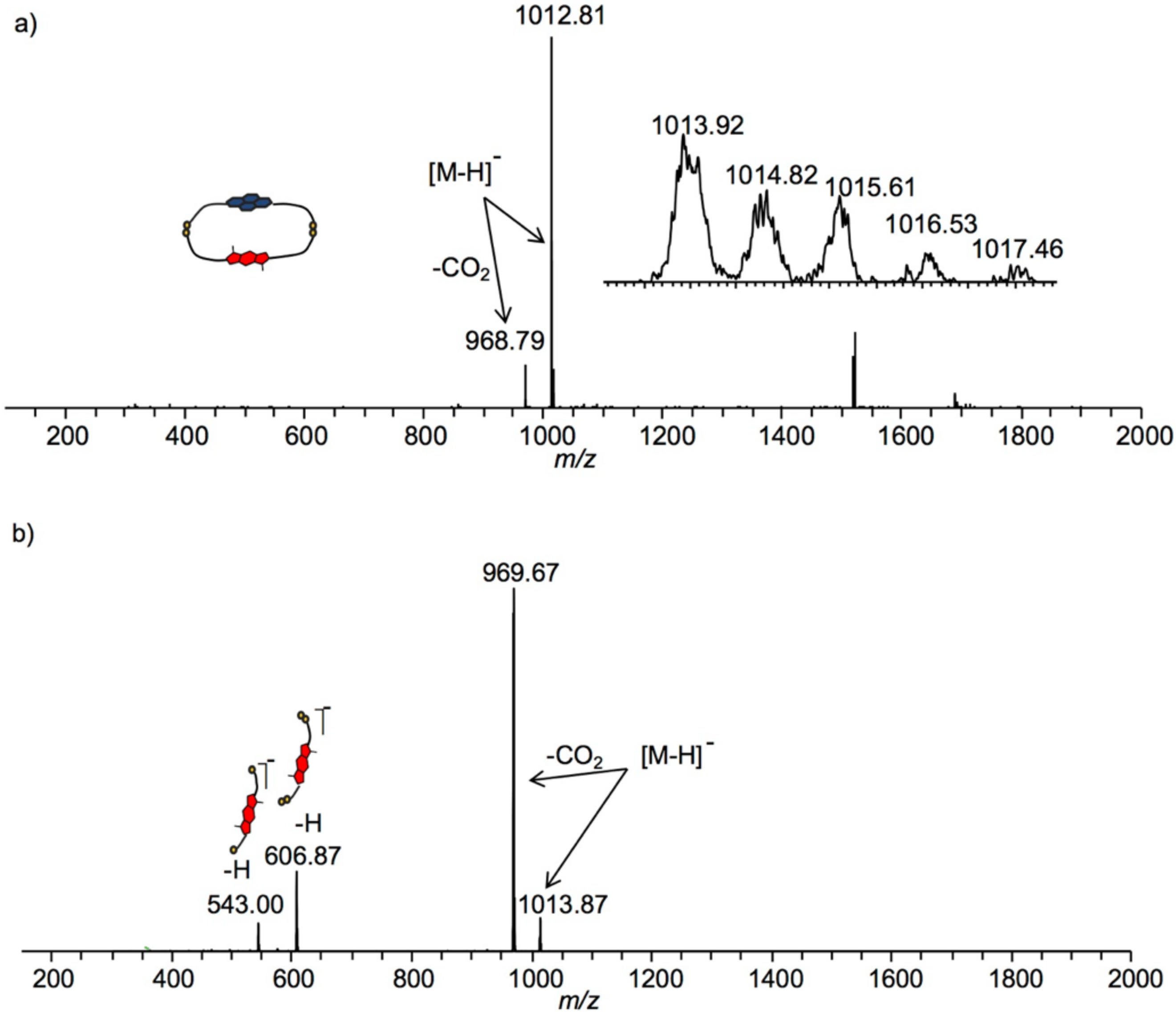

Supplementary Figure 61. a) MS (-*ve*) of **Z**; zoom of molecular ion is shown as inset. b) MS/MS (-*ve*) of **Z**.

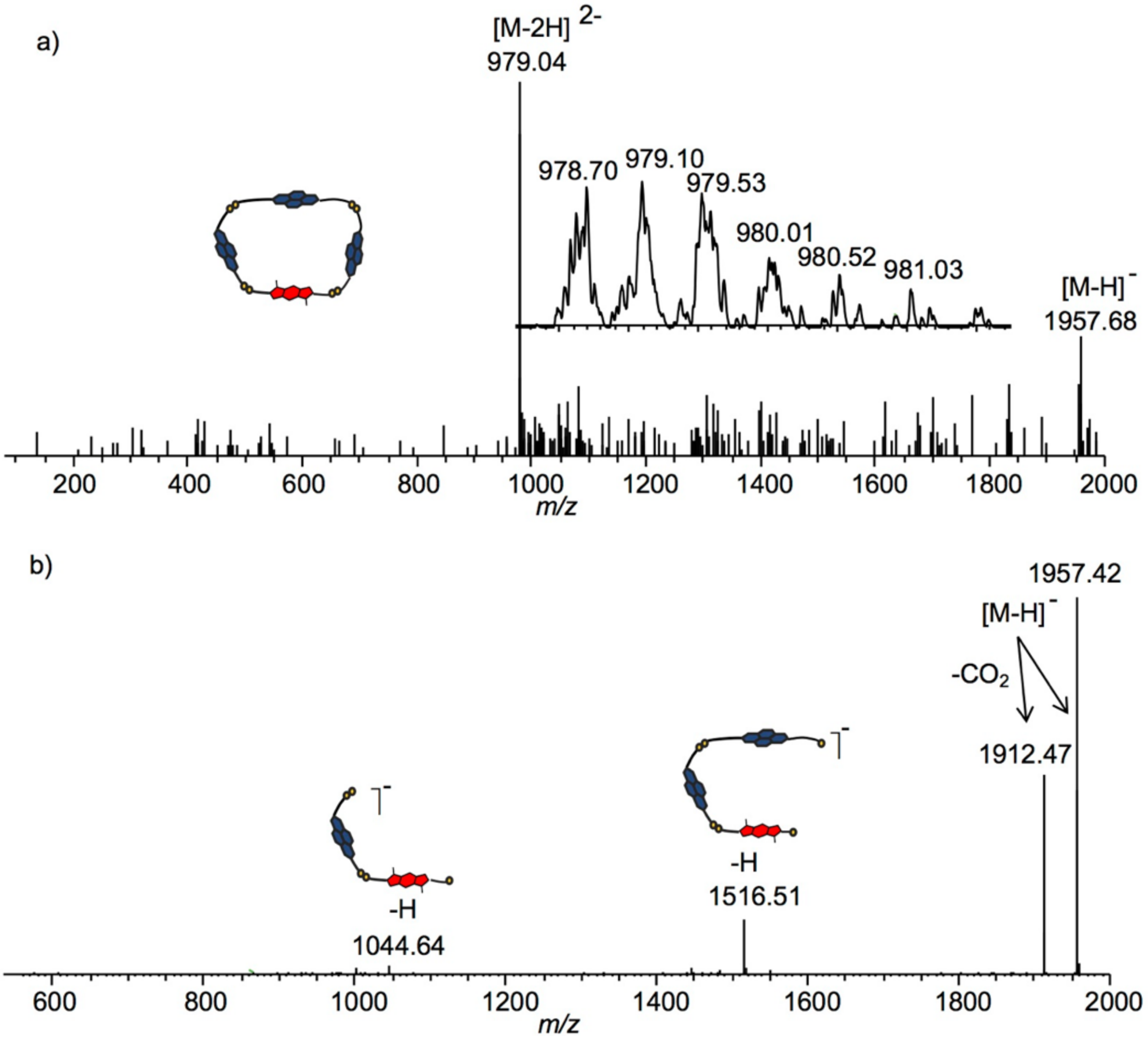

Supplementary Figure 62. a) MS (-*ve*) of heterotetramer based on three *S,S*-**1** units and one *R,R*-**2** unit; zoom of molecular ion is shown as inset. b) MS/MS (-*ve*) of heterotetramer.

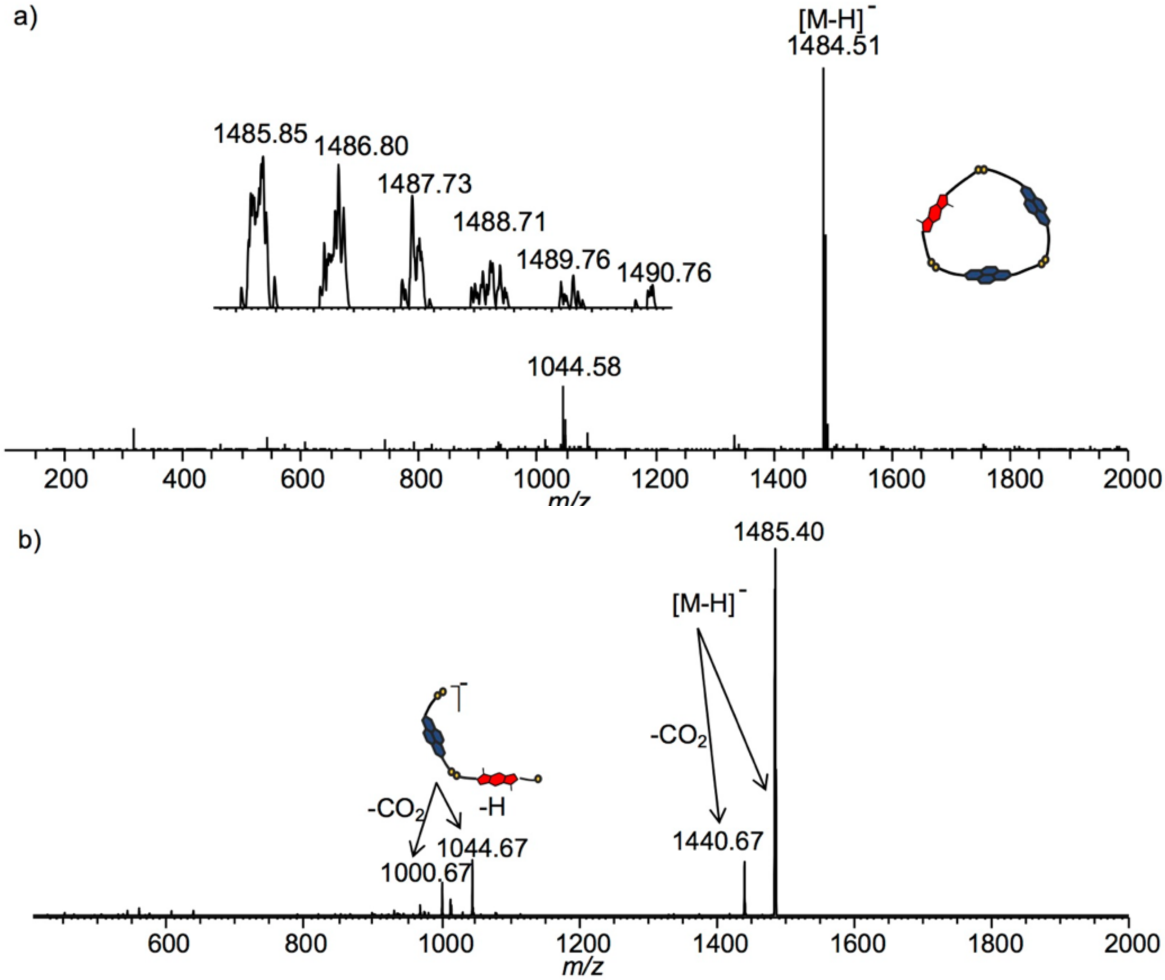

Supplementary Figure 63. a) MS (-*ve*) of heterotrimer based on two *S,S*-**1** units and one *R,R*-**2** unit; zoom of molecular ion is shown as inset. b) MS/MS (-*ve*) of heterotrimer.

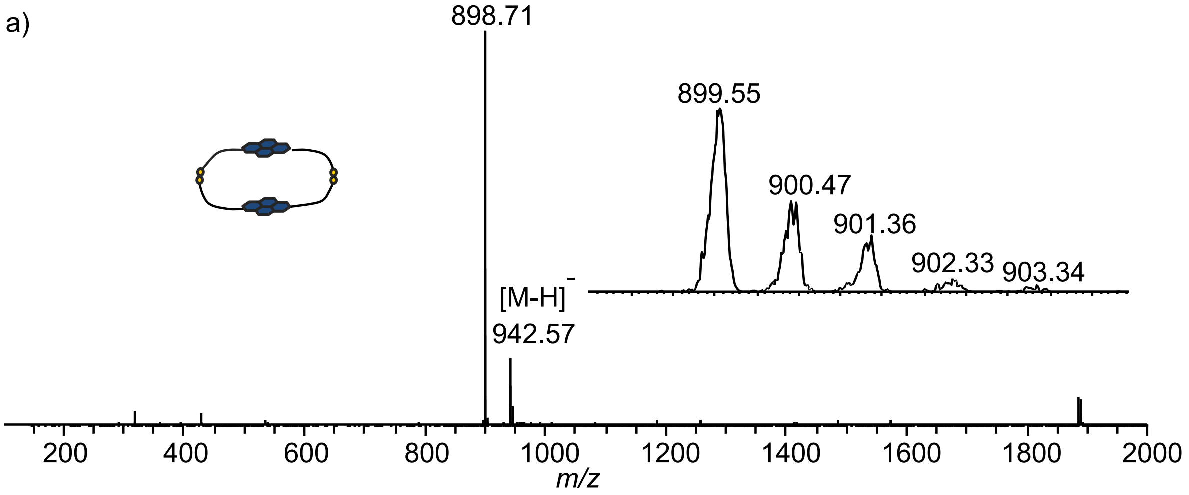

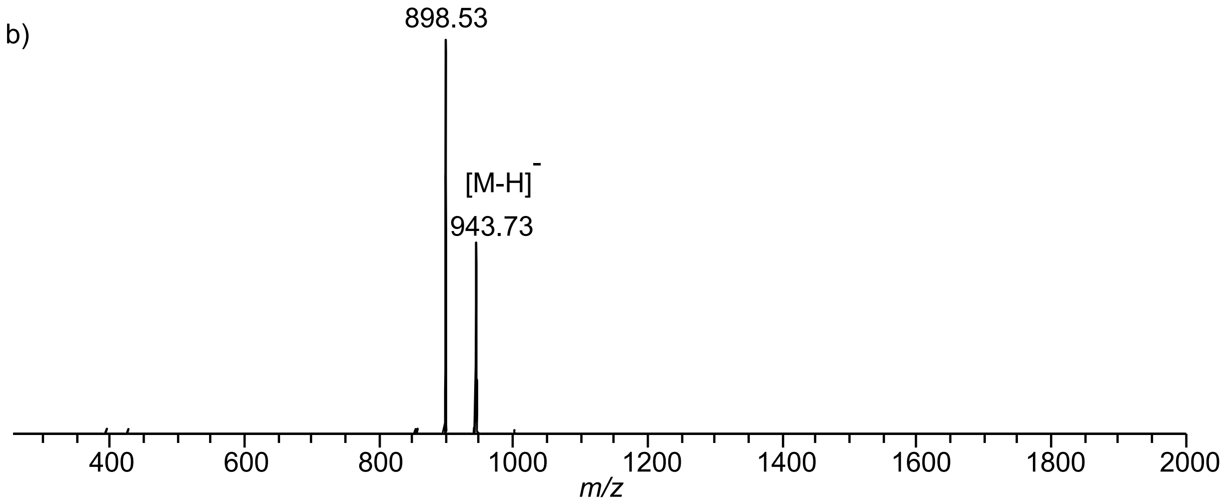

Supplementary Figure 64. a) MS (-*ve*) of *S,S*-**1** homodimer; zoom of molecular ion is shown as inset. b) MS/MS (-*ve*) of homodimer.

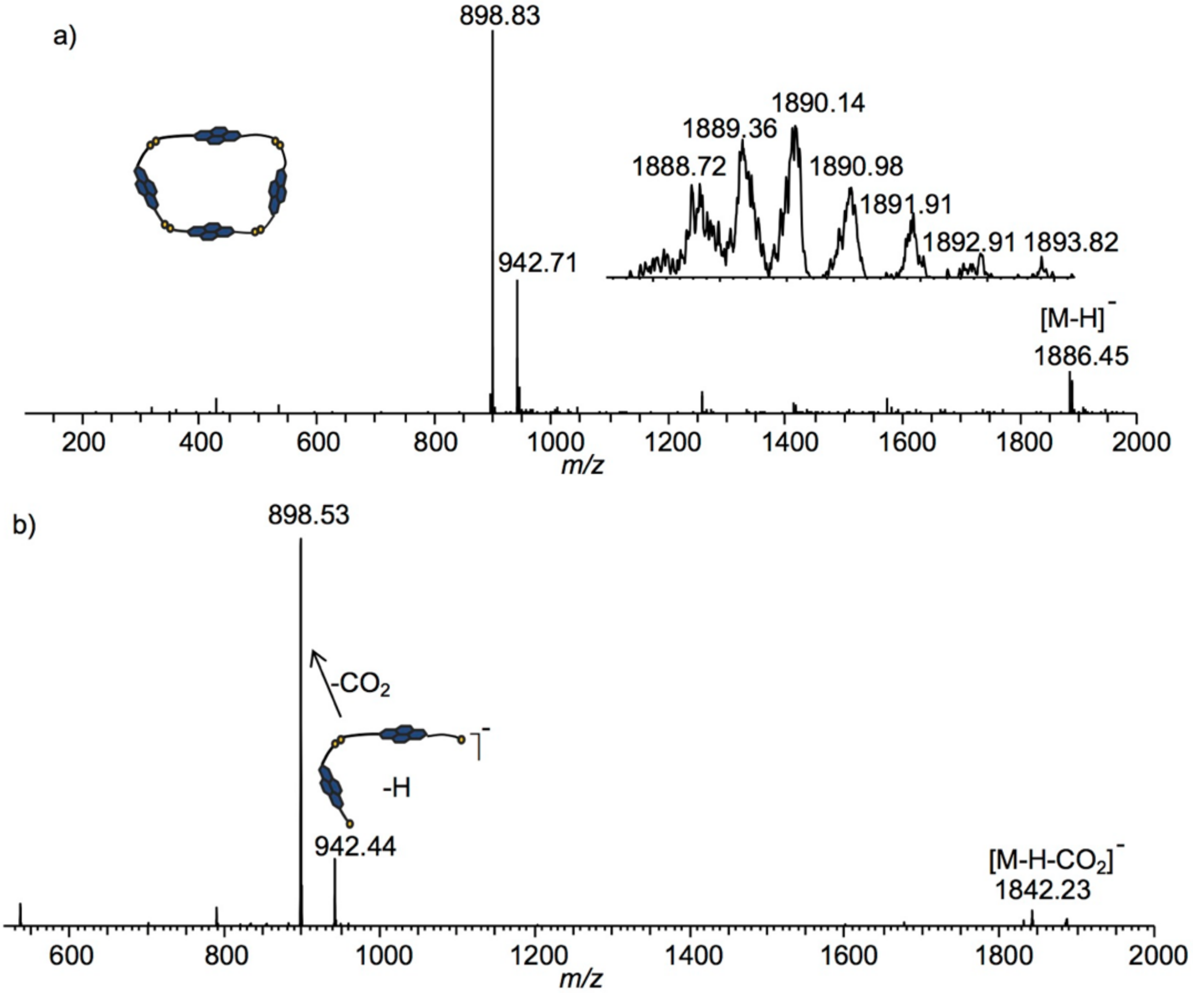

Supplementary Figure 65. a) MS (-*ve*) of *S,S*-**1** homotetramer; zoom of molecular ion is shown as inset. b) MS/MS (-*ve*) of homotetramer.

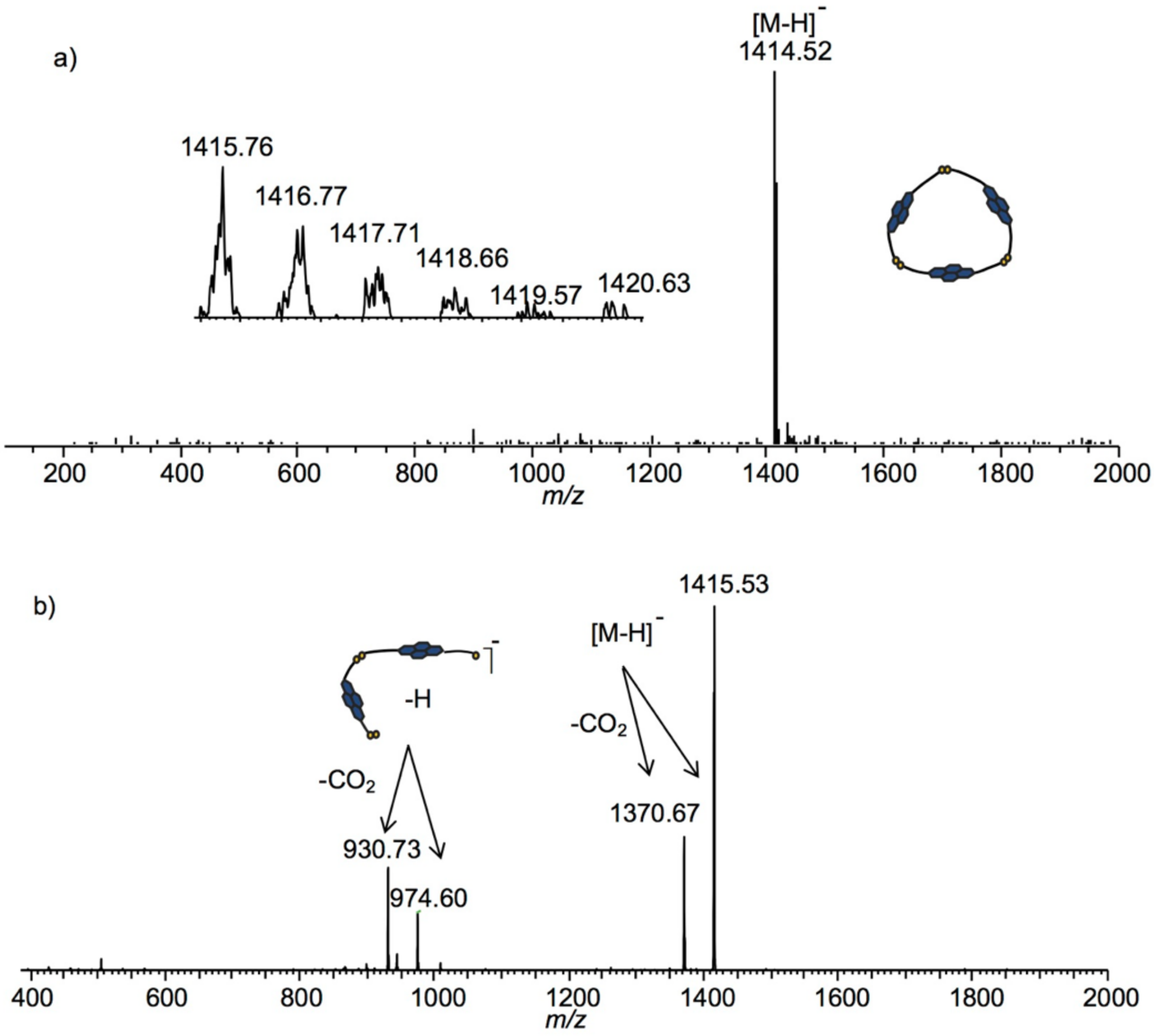

Supplementary Figure 66. a) MS (-*ve*) of *S,S*-**1** homotrimer; zoom of molecular ion is shown as inset. b) MS/MS (-*ve*) of homotrimer.

i)

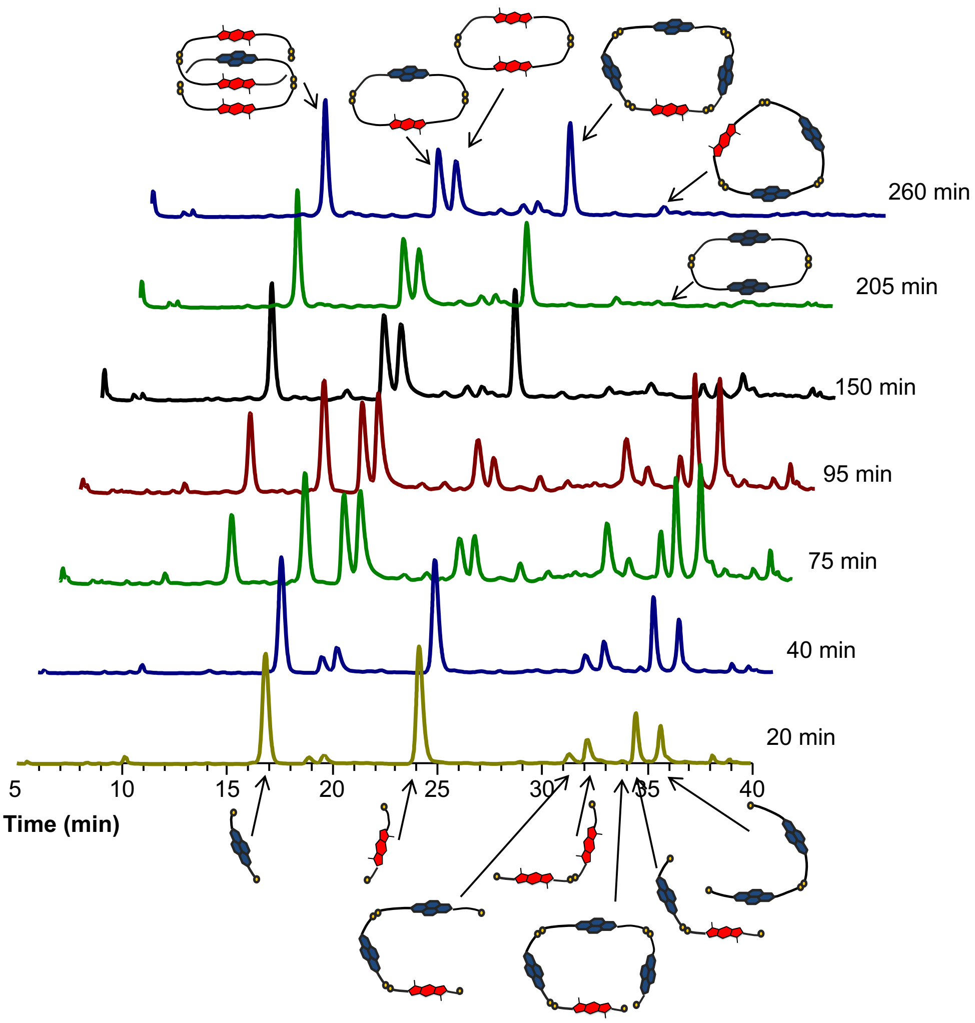

ii)

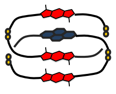	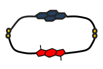	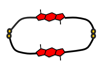	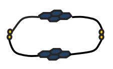	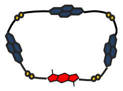	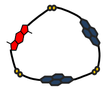
**Cat II RSRR**	**Z**	**Y**	*S,S*-**1** Dimer	*DAAA* (RSSS) Macrocycle	*DAA* (RSS) Macrocycle

iii)

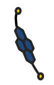	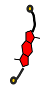	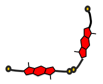	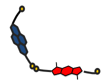	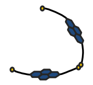	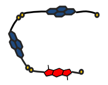	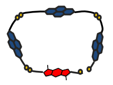
S,S-**1**	*R,R*-**2**	Linear **Y**	Linear **Z**	Linear *S,S*-**1** Dimer	Linear *DAA* (RSS)	Linear *DAAA* (RSSS)

Supplementary Figure 67. i) Reverse-phase HPLC analysis of *S,S*-**1**:*R,R*-**2** (1:1 molar ratio, 5 mM total concentration) library over time in the presence of 1 M $NaNO_3$. Absorbances recorded at 389 nm at different intervals: 260 min, 205 min, 150 min, 95 min, 75 min, 40min, 20 min as shown in figure. ii) and iii) The tables describe the annotation corresponding to each species.

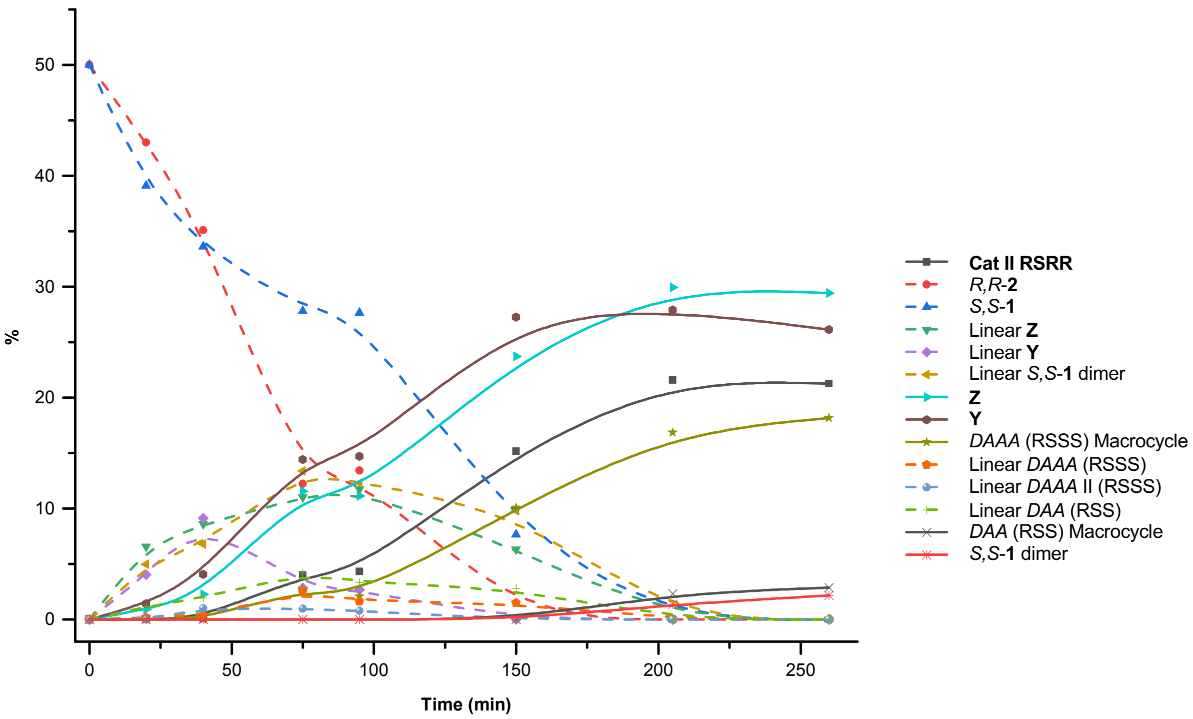

Supplementary Figure 68. Kinetic profile of *S,S*-**1**:*R,R*-**2** (1:1, 5 mM total concentration, absorption monitored at 389 nm) showing the formation of the **Cat II RSRR** (red). The description of each species can be seen in Legend (*D* stands for donor (BDT) and *A* stands for acceptor (NDI)). For clarity, spline curves were added; these do not represent mathematical models for kinetic data.

Based on pure statistical distribution, the ratio between Linear S,S-**1** dimer : Linear **Z** : Linear **Y** should be 1:2:1. This is not the case here, therefore linear **Y** is more thermodynamically stable than the other two linear species. The same is true for the corresponding cyclic dimers.

To a solution of **Y** (2.5 mM), *S,S*-**1** was added (as solid) in order to make a 5 mM total DCL concentration. After the pH was adjusted to 8-8.5, an HPLC chromatogram was recorded every 55 min. The experiment shows that the DCL is under thermodynamic control with the formation of **Cat II RSRR** as major species. This library resembles with the one formed from *S,S*-**1** and *R,R*-**2** in 1:1 ratio.

i)

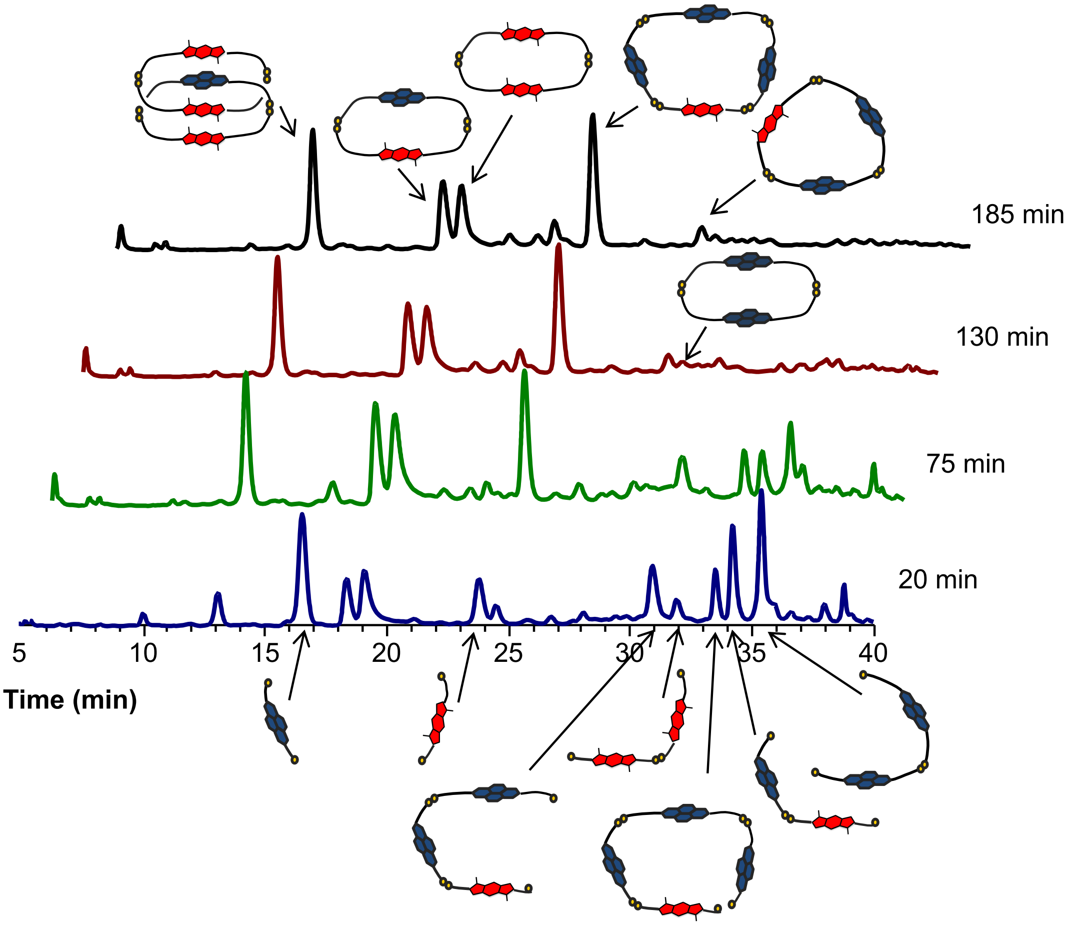

ii)

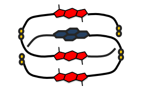	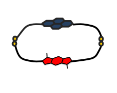	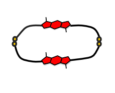	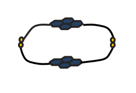	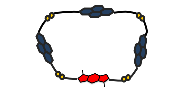	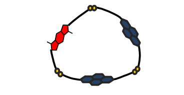
**Cat II RSRR**	**Z**	**Y**	*S,S*-**1** Dimer	*DAAA* (RSSS) Macrocycle	*DAA* (RSS) Macrocycle

iii)

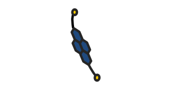	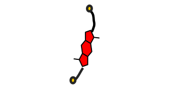	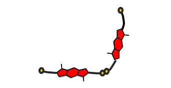	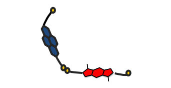	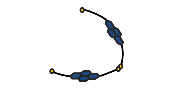	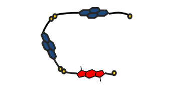	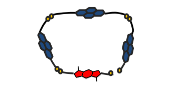
S,S-**1**	*R,R*-**2**	Linear **Y**	Linear **Z**	Linear *S,S*-**1** Dimer	Linear *DAA* (RSS)	Linear *DAAA* (RSSS)

Supplementary Figure 69. i) Kinetic profile of *S,S*-**1**:**Y** over time in the presence of 1 M $NaNO_3$. Absorbances recorded at 389 nm at different intervals: 185 min, 130 min, 75 min, 20 min as shown in figure. ii) and iii) The tables describe the annotation corresponding to each species.

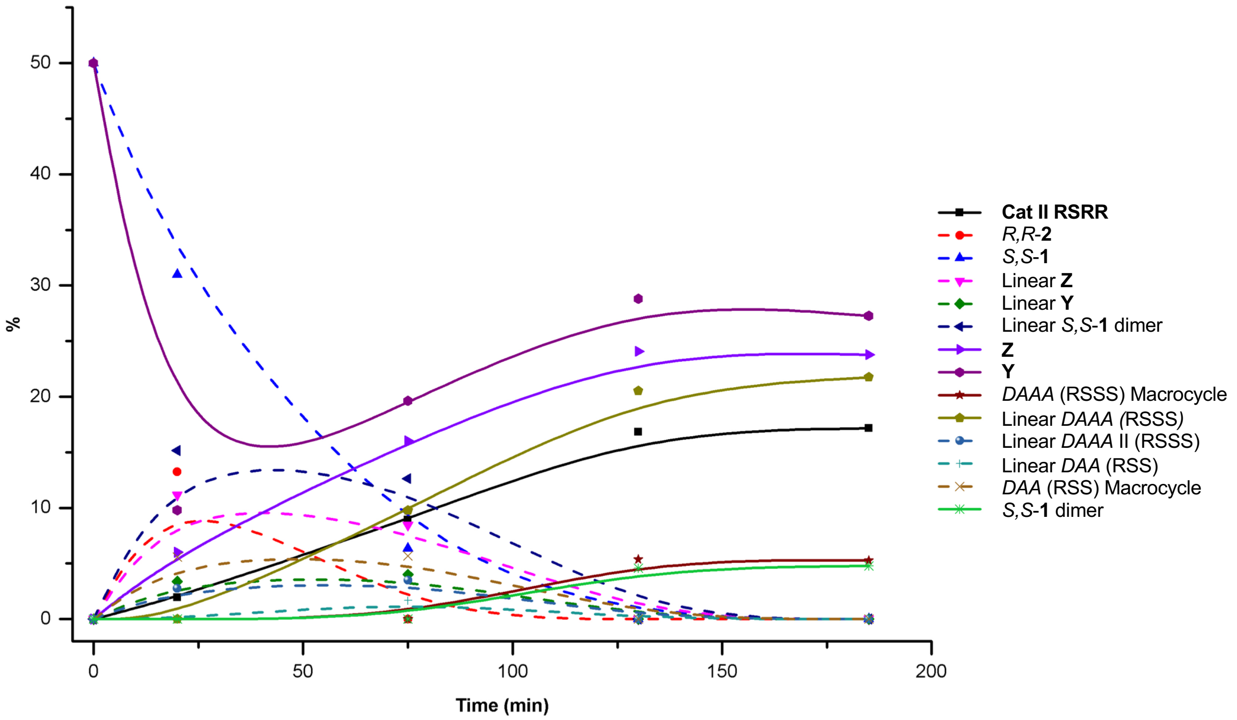

Supplementary Figure 70. Kinetic profile of *S,S*-**1**:**Y** (absorption monitored at 389 nm) showing the formation of the **Cat II RSRR** (black). The description of each species can be seen in legend (*D* stands for donor (BDT) and *A* stands for acceptor (NDI)). For clarity, spline curves were added; these do not represent mathematical models for kinetic data.

Analysis of DCLs containing *R,R*-1, *S,S*-1 and *R,R*-2

i)

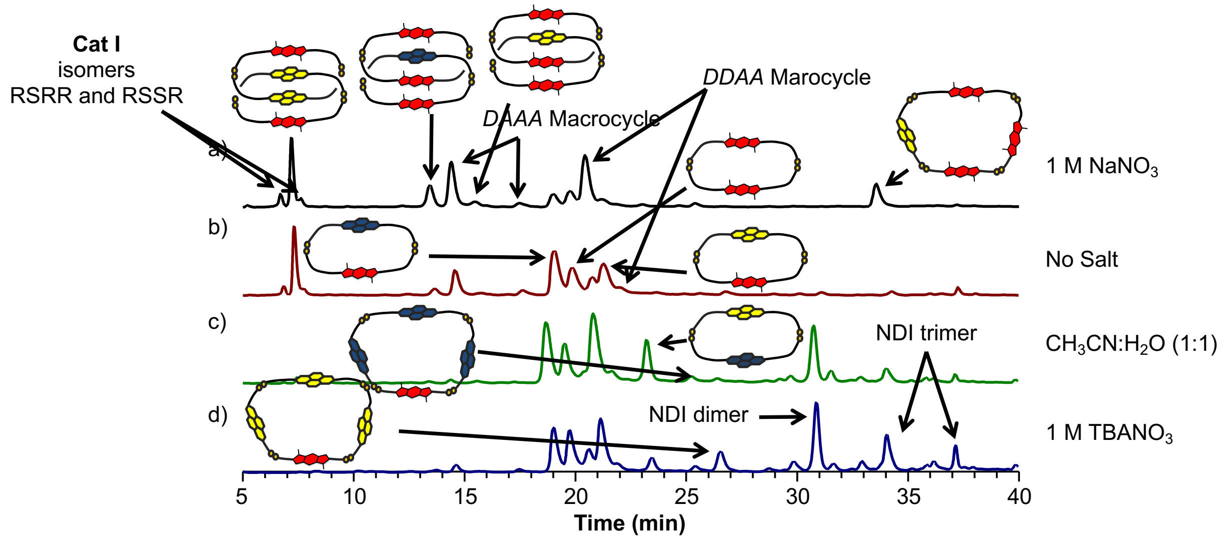

ii)

	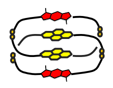	Cat I isomer I	Cat I isomer II	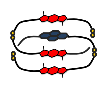	*DAAA* I Macrocycle	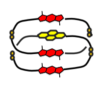	*DAAA* II Macrocycle	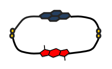	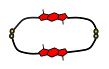	*DDAA* Macrocycle	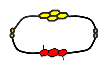
a)	13.67 (0.03)	2.52 (0.03)	2.16 (0.07)	7.68 (0.09)	15.92 (0.14)	2.33 (0.05)	1.77 (0.01)	5.63 (0.14)	8.53 (0.13)	18.46 (0.17)	7.54 (0.18)
b)	6.53 (0.08)	1.23 (0.01)	1.00 (0.02	1.31 (0.01)	6.10 (0.02)	-	1.58 (0.02)	23.46 (0.06)	14.24 (0.09)	3.37 (0.14)	31.41 (0.14)
c)	0.83 (0.00)	0.12 (0.00)	0.13 (0.00)	-	1.80 (0.01)	-	0.81 (0.01)	12.15 (0.05)	6.42 (0.13)	-	40.76 (0.04)
d)	-	-	-	-	1.06 (0.05)	-	0.34 (0.02)	10.52 (0.12)	10.81 (0.12)	4.05 (0.20)	4.05 (0.20)

iii)

	DDAA II Macrocycle	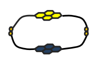	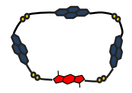	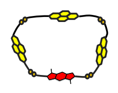	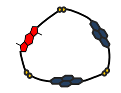	NDI Dimer	NDI Tetramer	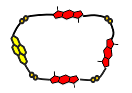	NDI I Trimer	NDI II Trimer
a)	-	2.82 (0.07)	1.61 (0.03)	-	-	-	-	9.35 (0.04)	-	-
b)	4.08 (0.09)	-	-	2.27 (0.11)	-	-	-	5.41 (0.03)	-	-
c)	2.40 (0.05)	8.38 (0.04)	1.48 (0.01)	0.70 (0.02)	1.53 (0.02)	16.92 (0.07)	2.18 (0.08)	3.38 (0.08)	-	-
d)	1.34 (0.23)	4.77 (0.05)	0.89 (0.08)	3.93 (0.05)	2.46 (0.05)	21.13 (0.04)	1.21 (0.01)	9.04 (0.18)	3.22 (0.08)	3.47 (0.26)

Supplementary Figure 71. i) Reverse-phase HPLC analysis of *R,R*-**2**:*R,R*-**1**:*S,S*-**1** (2:1:1 molar ratio, 5 mM total concentration) library a) in the presence of 1 M $NaNO_3$, b) no salt, c) in a mixture of $CH_3CN:H_2O$ (1:1) and d) in the presence of 1 M $TBANO_3$. Absorbances recorded at 389 nm. ii) and iii) The values in the tables represent the percentage of each species identified in the chromatograms above (i). The integrations were done in triplicate and the RMSD is reported in parenthesis along with the value. Each row of the table corresponds to the chromatogram bearing the same identification in the figure above. The unlabelled peaks did not ionise and could not be identified.

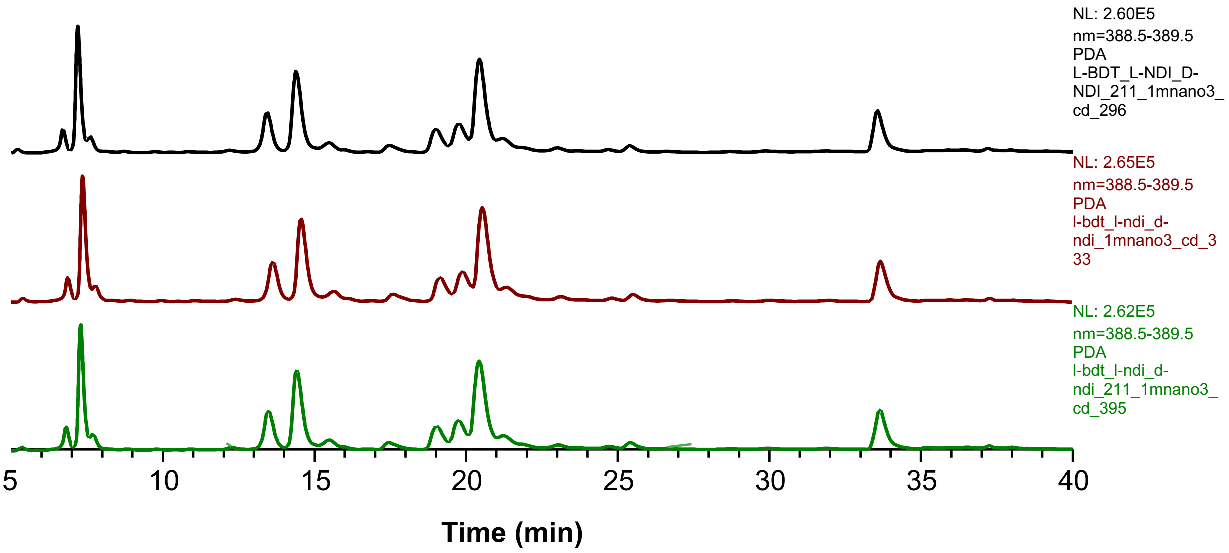

Supplementary Figure 72. Reverse-phase HPLC analysis of *R,R*-**1**:*S,S*-**1**:*R,R*-**2** (1:1:2) molar ratio, 5 mM total concentration) library in the presence of 1 M $NaNO_3$ of the same library analysed at different times.

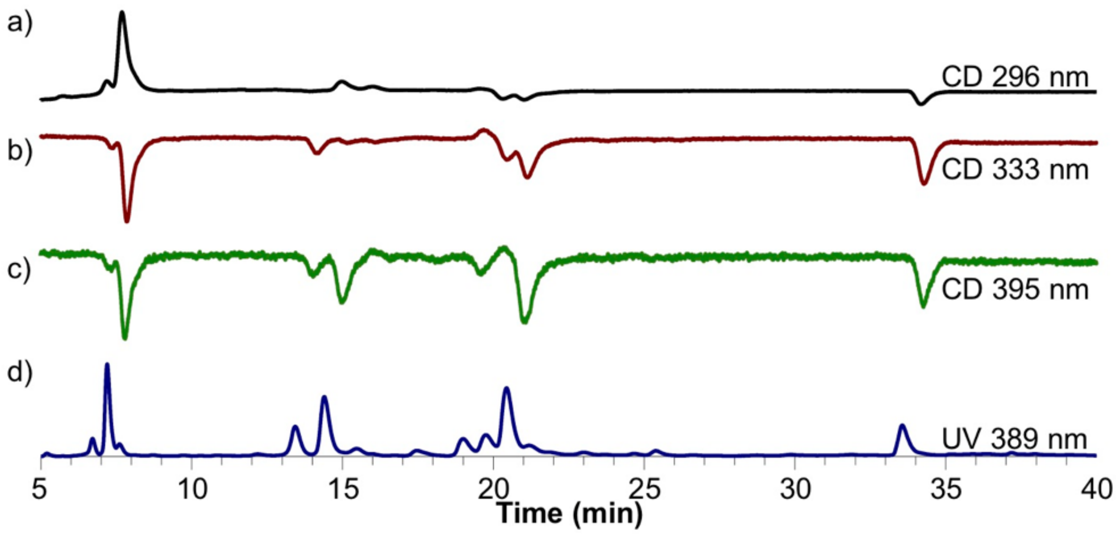

Supplementary Figure 73. Reverse-phase HPLC analysis of *R,R*-**2**:*R,R*-**1**:*S,S*-**1** (2:1:1 molar ratio, 5 mM total concentration) library in the presence of 1 M $NaNO_3$, showing a) CD at 296 nm, b) CD at 333 nm, c) CD at 395 nm and d) absorbance at 389 nm.

i)

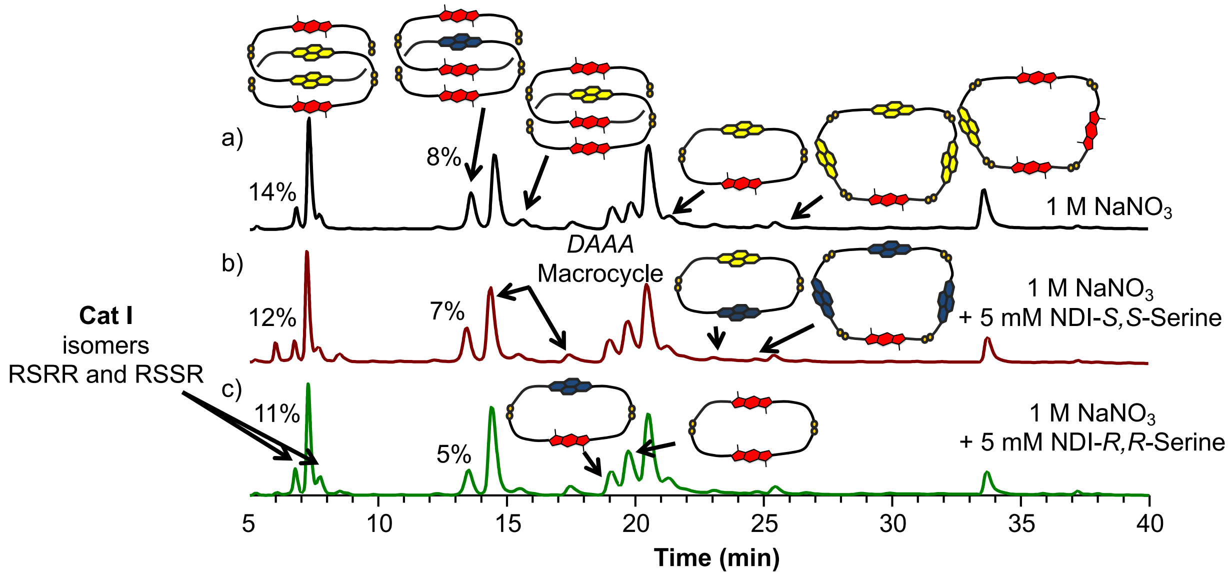

ii)

	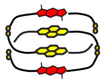	Cat I isomer I	Cat I isomer II	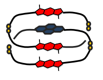	*DAAA* I Macrocycle	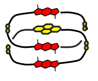	*DAAA* II Macrocycle	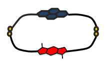
a)	13.67 (0.03)	2.52 (0.03)	2.16 (0.07)	7.68 (0.09)	15.92 (0.14)	2.33 (0.05)	1.77 (0.01)	5.63 (0.14)
b)	12.05 (0.12)	2.50 (0.02)	2.53 (0.02)	7.19 (0.03)	15.50 (0.21)	2.03 (0.01)	2.14 (0.01)	5.83 (0.01)
c)	11.49 (0.06)	2.88 (0.02)	2.69 (0.01)	4.90 (0.04)	16.78 (0.07)	1.67 (0.04)	2.17 (0.04)	6.15 (0.11)

iii)

	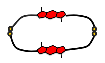	*DDAA* Macrocycle	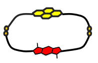	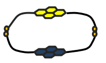	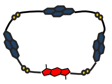	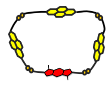	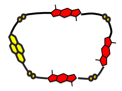
a)	8.53 (0.13)	18.46 (0.07)	7.54 (0.18)	2.82 (0.13)	1.61 (0.03)	-	9.35 (0.04)
b)	15.86 (0.04)	16.28 (0.04)	9.12 (0.04)	3.05 (0.17)	-	0.88 (0.05)	5.04 (0.17)
c)	16.60 (0.10)	15.98 (0.03)	9.08 (0.06)	2.75 (0.14)	0.82 (0.03)	0.91 (0.05)	5.08 (0.01)

Supplementary Figure 74. i) Reverse-phase HPLC analysis of *R,R*-**2**:*R,R*-**1**:*S,S*-**1** (2:1:1 molar ratio, 5 mM total concentration) library a) in the presence of 1 M $NaNO_3$, b) 1 M $NaNO_3$ + 5 mM **NDI-*S,S*-serine** and c) 1 M $NaNO_3$ + 5 mM **NDI-*R,R*-serine**. Absorbances recorded at 389 nm. ii) and iii) The values in the tables represent the percentage of each species identified in the chromatograms above (i). The integrations were done in triplicate and the RMSD is reported in parenthesis along with the value. Each row of the table corresponds to the chromatogram bearing the same identification in the figure above. The unlabelled peaks did not ionise and could not be identified.

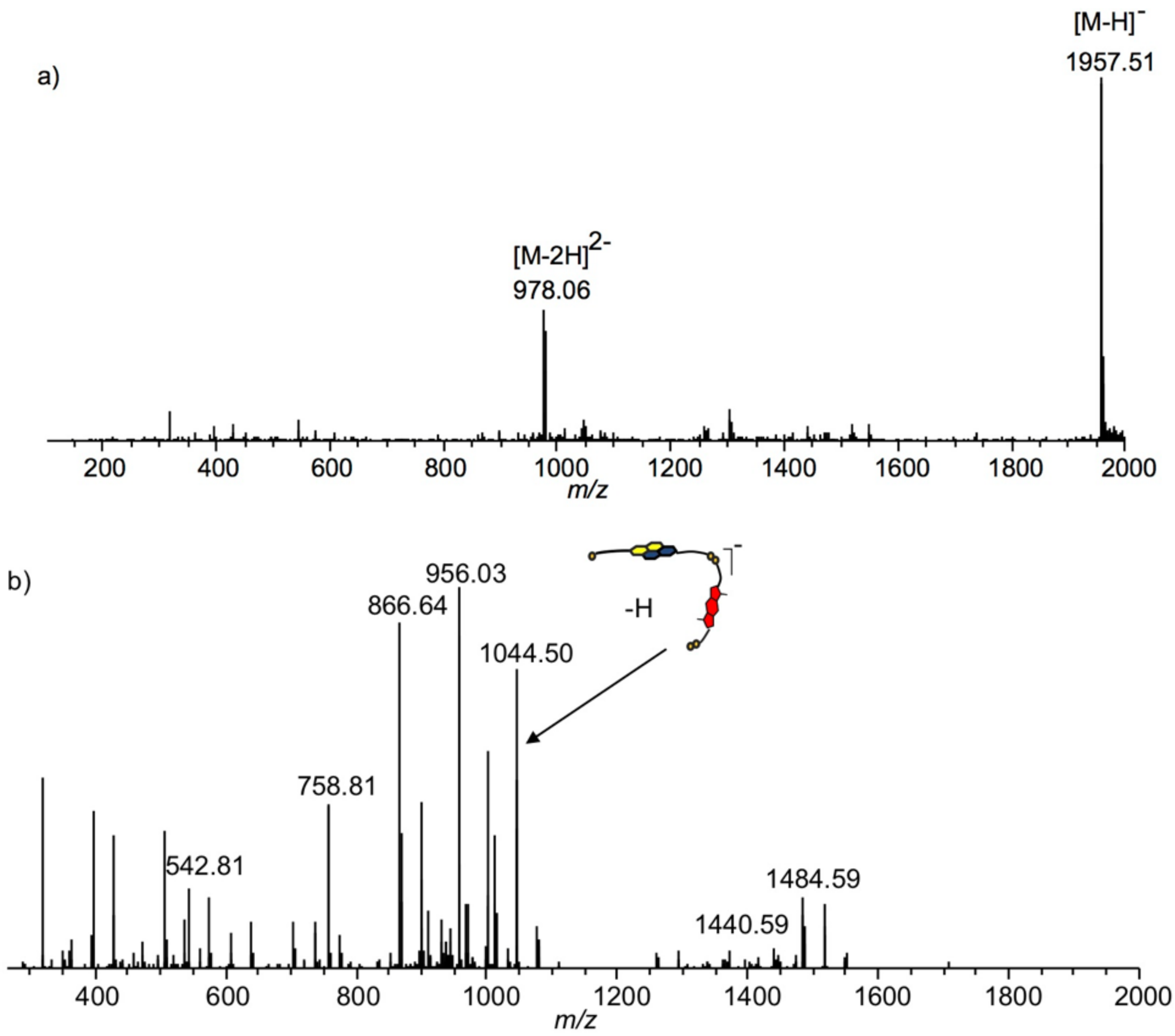

Supplementary Figure 75. a) MS (-*ve*) of heterotetramer based on three *R,R*-**1**/*S,S*-**1** units and one *R,R*-**2** unit, b) MS/MS (-*ve*) of heterotetramer.

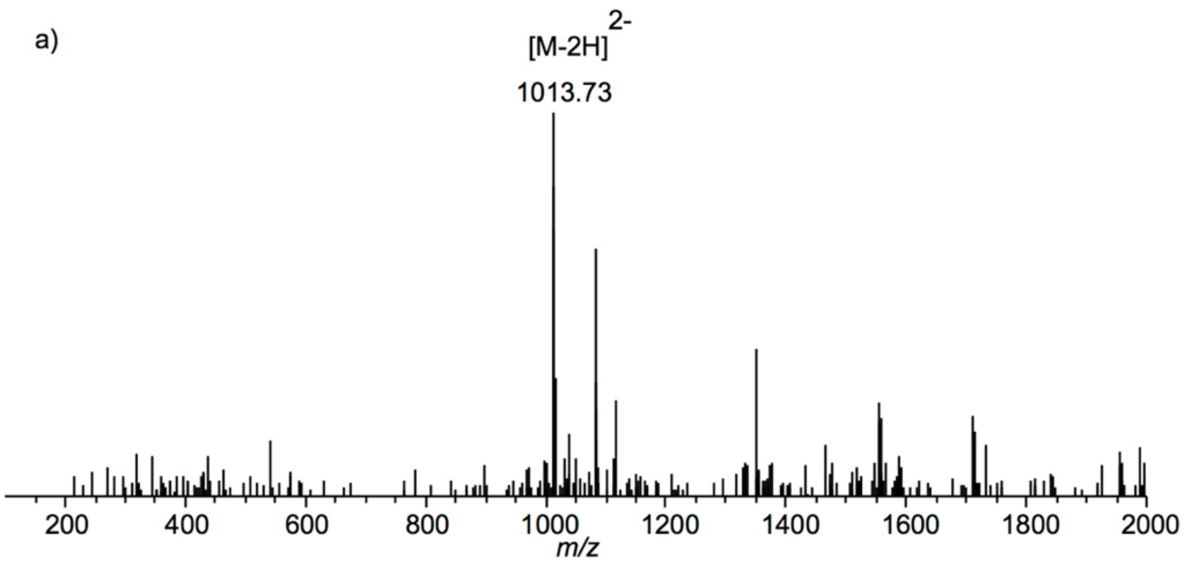

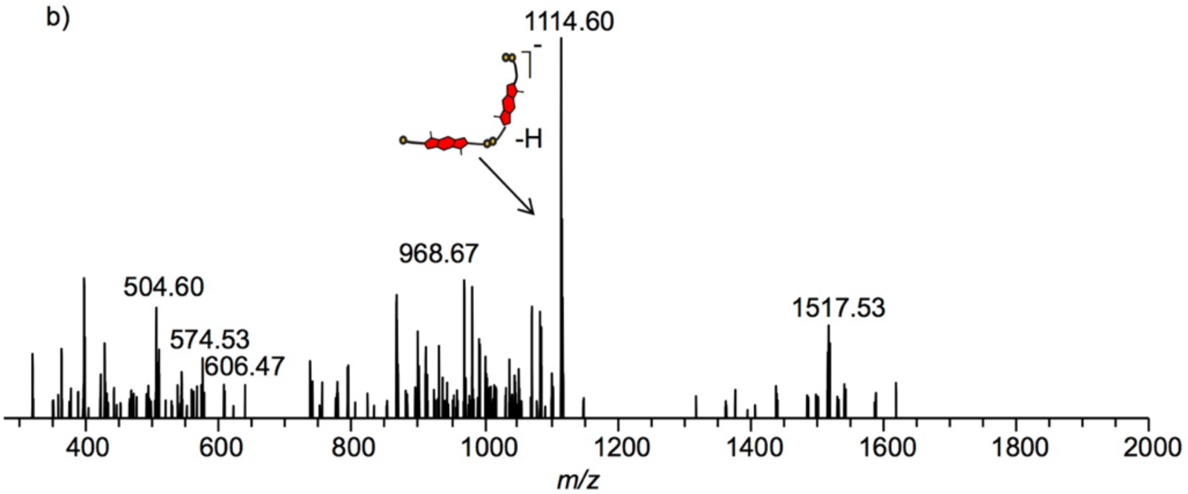

Supplementary Figure 76. a) MS (-*ve*) of heterotetramer based on two *R,R*-**1**/*S,S*-**1** units and two *R,R*-**2** units, b) MS/MS (-*ve*) of heterotetramer.

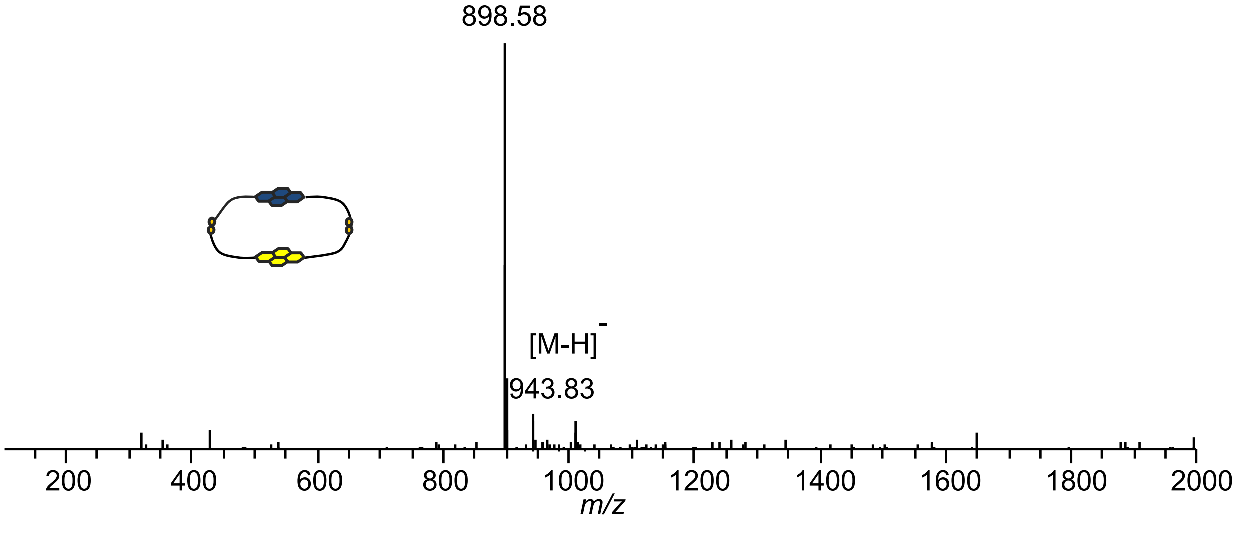

Supplementary Figure 77. MS (-*ve*) of heterochiral NDI dimer.

i)

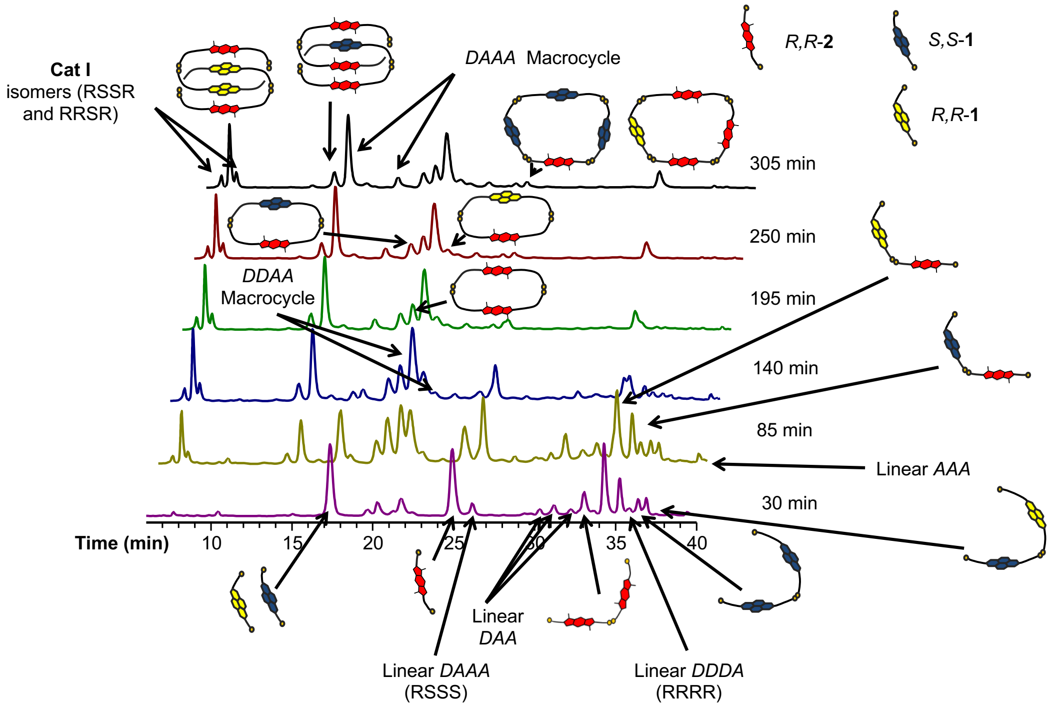

ii)

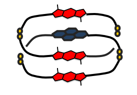	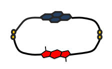	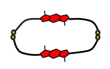	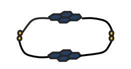	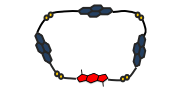	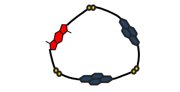
**Cat II RSRR**	**Z**	**Y**	*S,S*-**1** Dimer	*DAAA* (RSSS) Macrocycle	*DAA* (RSS) Macrocycle

iii)

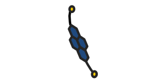	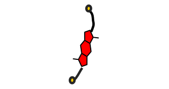	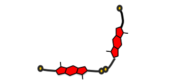	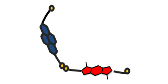	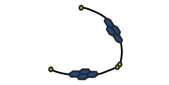	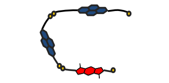	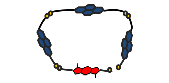
S,S-**1**	*R,R*-**2**	Linear **Y**	Linear **Z**	Linear *S,S*-**1** Dimer	Linear *DAA* (RSS)	Linear *DAAA* (RSSS)

iv)

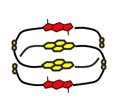	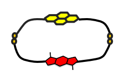	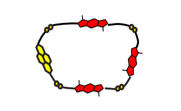	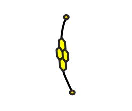	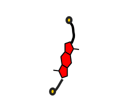	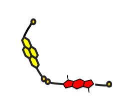	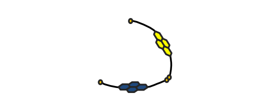
**Cat I RRRR**	**X**	*DDDA* (RRRR) Macrocycle	*R,R*-**1**	*R,R*-**2**	Linear **X**	Linear NDI Dimer (mixed chiralities)

Supplementary Figure 78. i) Reverse-phase HPLC analysis of *R,R*-**2**:*R,R*-**1**:*S,S*-**1** (2:1:1 molar ratio, 5 mM total concentration) library over time in the presence of 1 M $NaNO_3$. Absorbances recorded at 389 nm at different intervals: 305 min, 250 min, 195 min, 140 min, 85 min, 30 min, as shown in figure. ii), iii) and iv) The tables describe the annotation corresponding to each species.

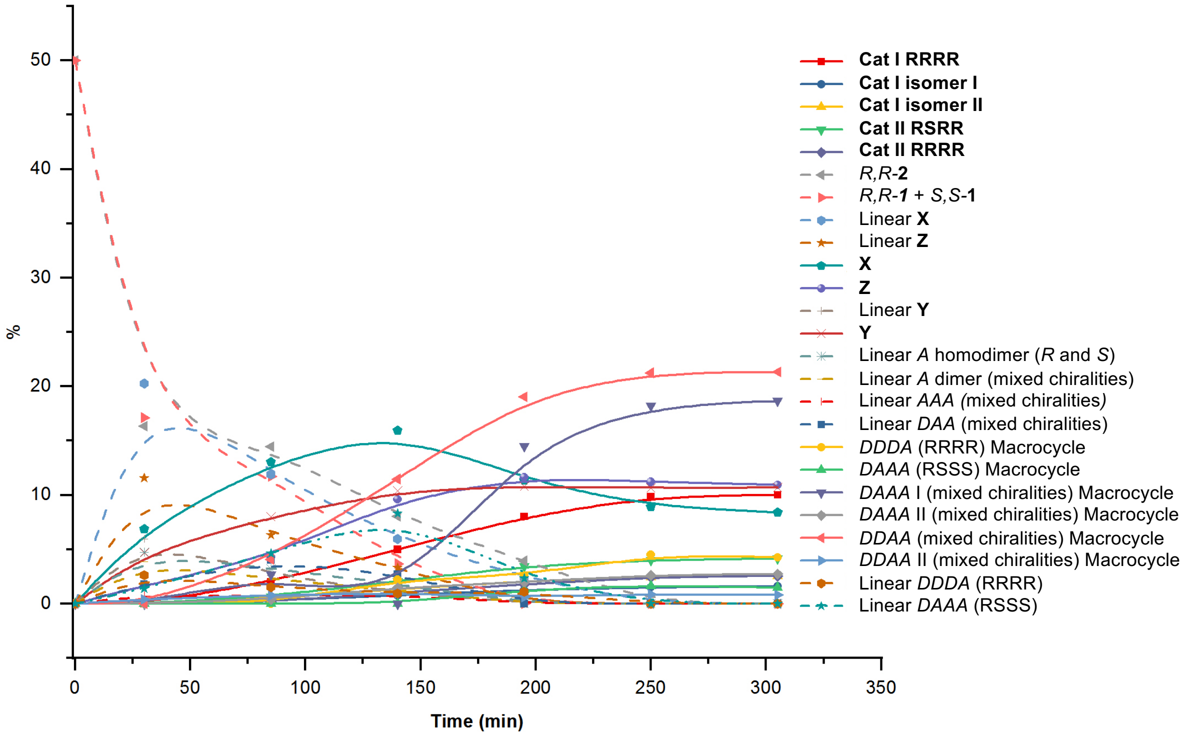

Supplementary Figure 79. Kinetic profile of *S,S*-**1**:*R,R*-**2** (1:1, 5 mM total concentration, absorption monitored at 389 nm) showing the formation of **Cat I RRRR** and **Cat II RSRR**. The description of each species can be seen in legend (*D* stands for donor (BDT) and *A* stands for acceptor (NDI)). For clarity, spline curves were added; these do not represent mathematical models for kinetic data.

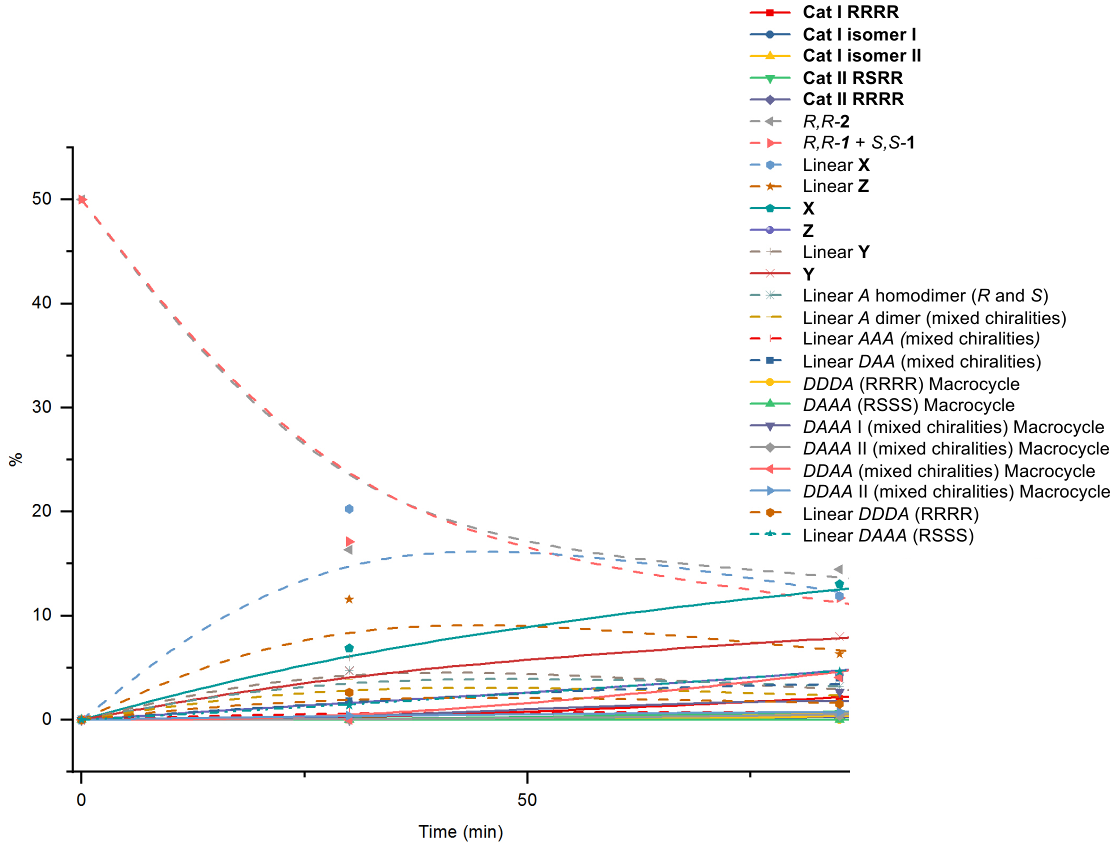

Supplementary Figure 80. Zoom region between 0 and 85 min for the **Supplementary Figure 79.**

i)

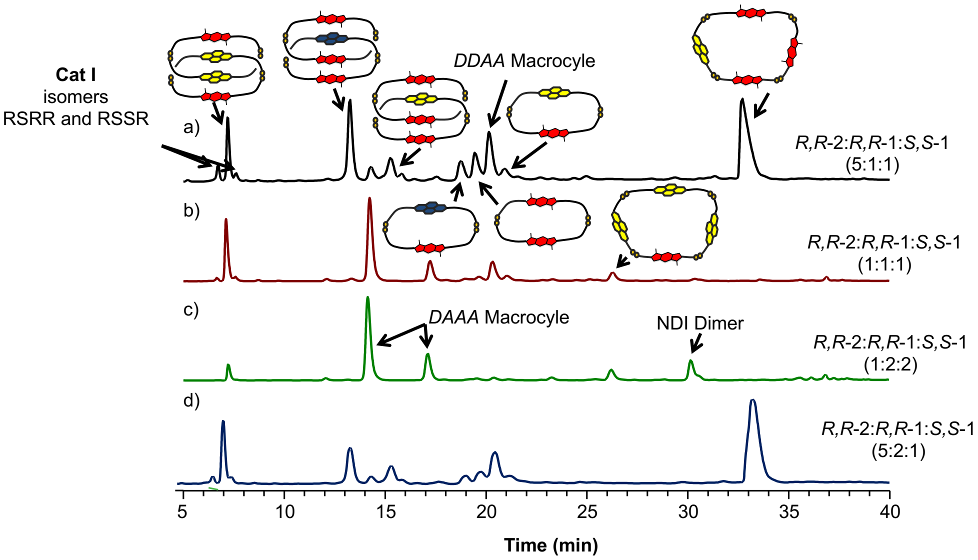

ii)

	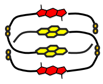	Cat I isomer I	Cat I isomer II	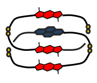	*DAAA* I Macrocycle	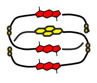	*DAAA* II Macrocycle
a)	6.59 (0.01)	1.86 (0.02)	0.99 (0.00)	14.50 (0.06)	3.11 (0.08)	5.99 (0.09)	
b)	17.57 (0.02)	0.94 (0.00)	1.51 (0.01)	1.54 (0.01)	38.43 (0.05)	-	10.27 (0.03)
c)	5.78 (0.10)	-	-	-	47.49 (0.42)	-	16.22 (0.19)
d)	10.09 (0.14)	0.88 (0.26)	0.88 (0.02)	9.68 (0.21)	1.46 (0.10)	5.35 (0.17)	-

iii)

	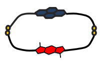	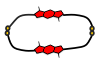	*DDAA* Macrocycle	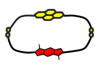	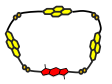	NDI Dimer	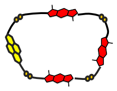
a)	9.40 (0.10)	12.44 (0.03)	10.44 (0.05)	7.15 (0.04)	-	-	27.53 (0.07)
b)	2.75 (0.01)	4.55 (0.01)	9.77 (0.01)	7.30 (0.02)	5.38 (0.05)	-	-
c)	-	-	-	-	7.41 (0.24)	23.11 (0.58)	-
d)	5.20 (0.58)	8.51 (0.52)	11.88 (0.33)	5.54 (0.05)		-	41.88 (0.77)

Supplementary Figure 81. i) Reverse-phase HPLC analysis of a) *R,R*-**2**:*R,R*-**1**:*S,S*-**1** (5:1:1 molar ratio, 5 mM total concentration) library in the presence of 1 M $NaNO_3$, b) *R,R*-**2**:*R,R*-**1**:*S,S*-**1** (1:1:1 molar ratio, 5 mM total concentration) library in the presence of 1 M $NaNO_3$, c) *R,R*-**2**:*R,R*-**1**:*S,S*-**1** (1:2:2 molar ratio, 5 mM total concentration) library

in the presence of 1 M $NaNO_3$, d) *R,R*-**2**:*R,R*-**1**:*S,S*-**1** (5:2:1 molar ratio, 5 mM total concentration) library in the presence of 1 M $NaNO_3$. Absorbances recorded at 389 nm. ii) and iii) The values in the tables represent the percentage of each species identified in the chromatograms above. The integrations were done in triplicate and the RMSD is reported in parenthesis along with the value. Each row of the table corresponds to the chromatogram bearing the same identification in the figure above. The unlabelled peaks did not ionise and could not be identified.

It is important to mention that the DCL in Fig. S81 (d) is not representative for an SDRRM as ratio *R,R*-**1**:*S,S*-**1** is 2:1. This library was setup in an effort to maximise the proportion of **Cat I RRRR** and **Cat II RSRR** in the DCL.

UV-Vis / CD spectra of building blocks

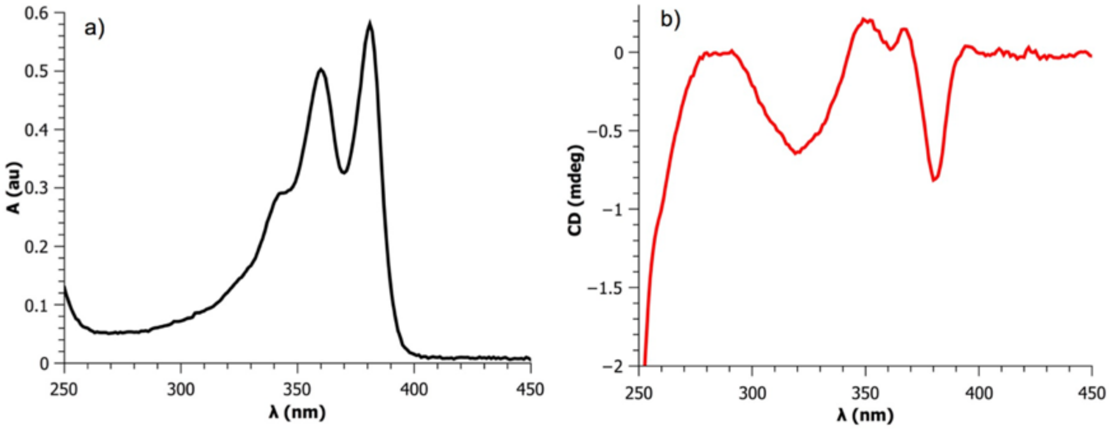

Supplementary Figure 82. a) UV-Vis spectrum, b) CD spectrum of *R,R*-**1** at 23 °C in CH_3CN / H_2O mixture (66.66:33.34).

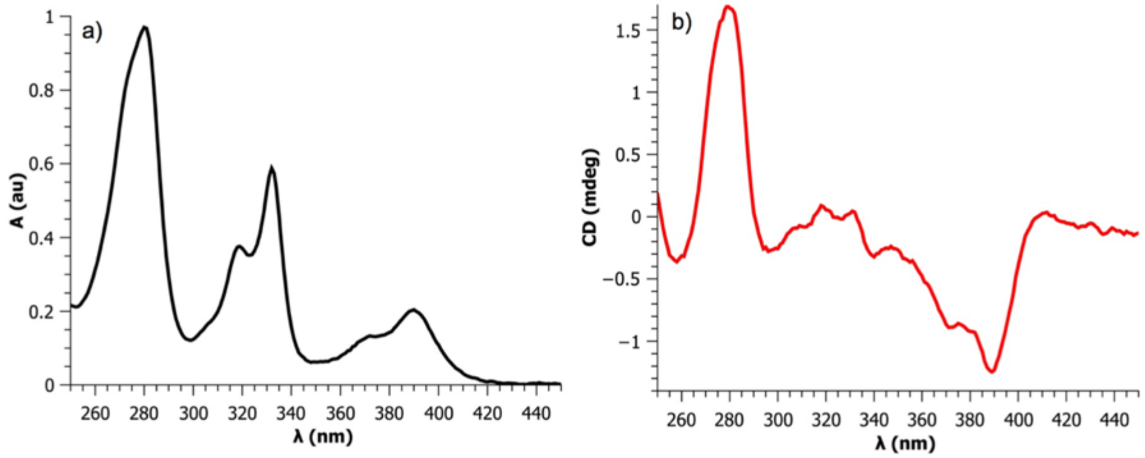

Supplementary Figure 83. a) UV-Vis spectrum, b) CD spectrum of *R,R*-**2** at 23 °C. CH_3CN / H_2O mixture (66.66:33.34).

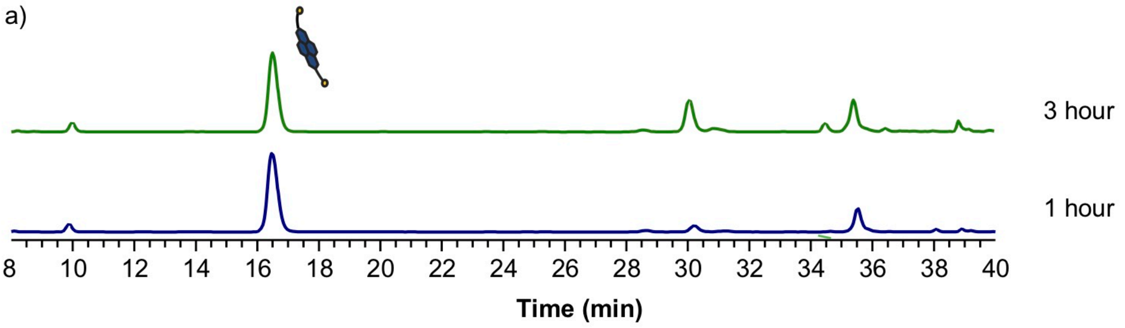

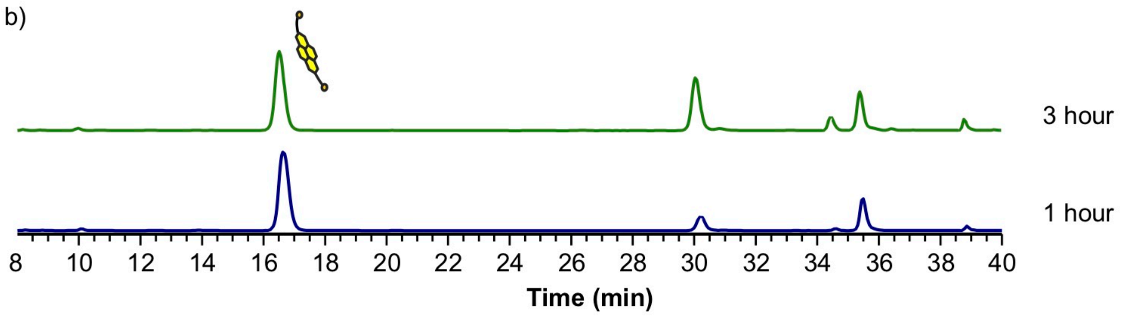

Supplementary Figure 84. Reverse-phase HPLC analysis of a) *S,S*-**1** (2.5 mM total concentration) after 1 and 3 hours, b) *R,R*-**1** (2.5 mM total concentration) after 1 and 3 hours.

The experiment was done to prove the similar behaviour of the NDI enantiomers *R,R*-**1** and *S,S*-**1**.

UV-Vis titration of NDI-*R,R*/*S,S*-serine into Y

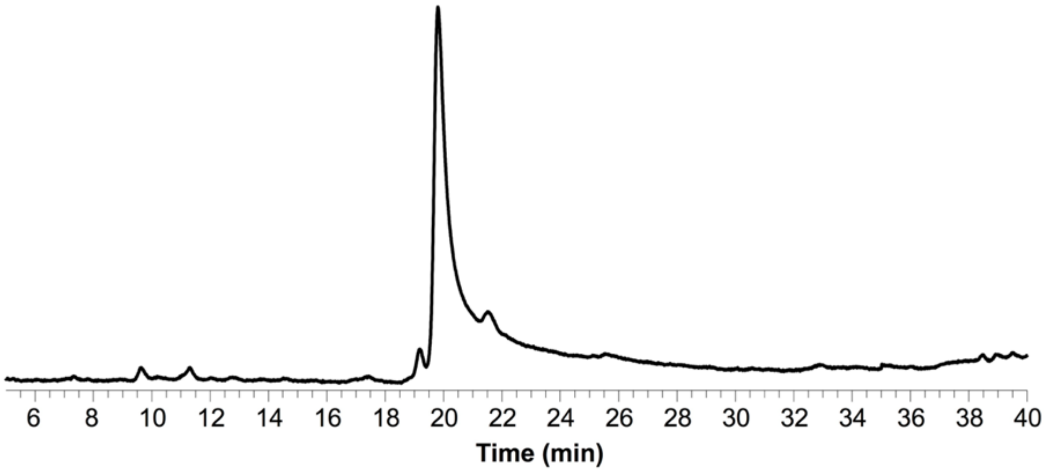

Supplementary Figure 85. Reverse-phase HPLC analysis of *R,R*-**2** (5 mM total concentration) library. Absorbance was recorded at 389 nm.

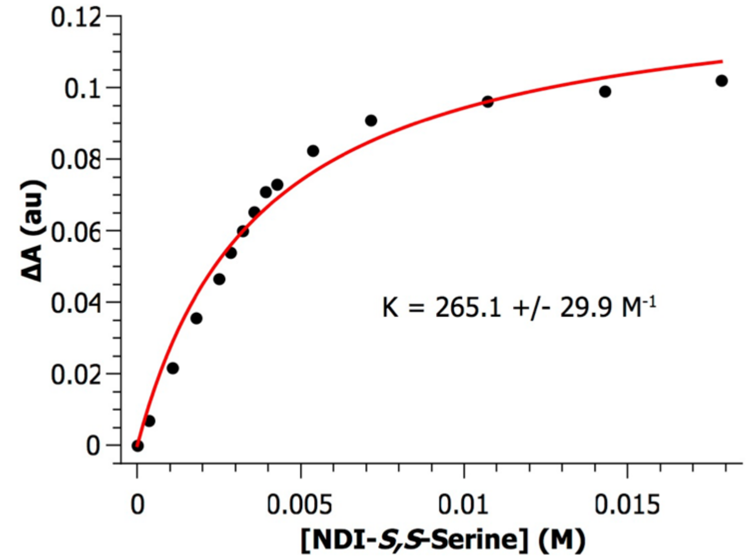

Supplementary Figure 86. Fitting of UV-Vis titration data points of the binding event between **Y** and **NDI-*S,S*-serine**.

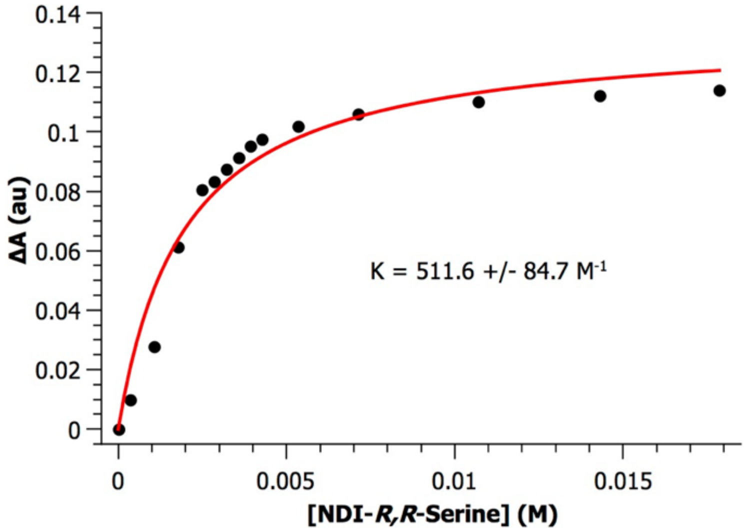

Supplementary Figure 87. Fitting of UV-Vis titration data points of the binding event between **Y** and **NDI-*R,R*-serine**.

The results of the experiments indicate that NDI-*R,R*-serine binds better in the cavity of **Y** with an almost double K_a in comparison with NDI-*S,S*-serine. The results are in good agreement with the templated libraries with NDI-*R,R*-serine and NDI-*S,S*-serine. In the case of **Cat I RRRR**, the yield is lowered when NDI-*S,S*-serine is used as a template, whereas in the case of **Cat II RSRR**, the yield is lowered by addition of NDI-*R,R*-serine. In each case the NDI-serine competes with the threading of the building block / linear heterodimer into the respective closed heterodimer. From these experiments we can conclude that K_{a6}>K_{d5} (from **Supplementary Figure 25**).

Testing reversibility with DTT

As can be seen below, both libraries were reformed during the experiments, proving their reversible character. **Cat II RSRR** has reformed in a lower yield, because the concentration was reduced compared to **Cat I RRRR**.

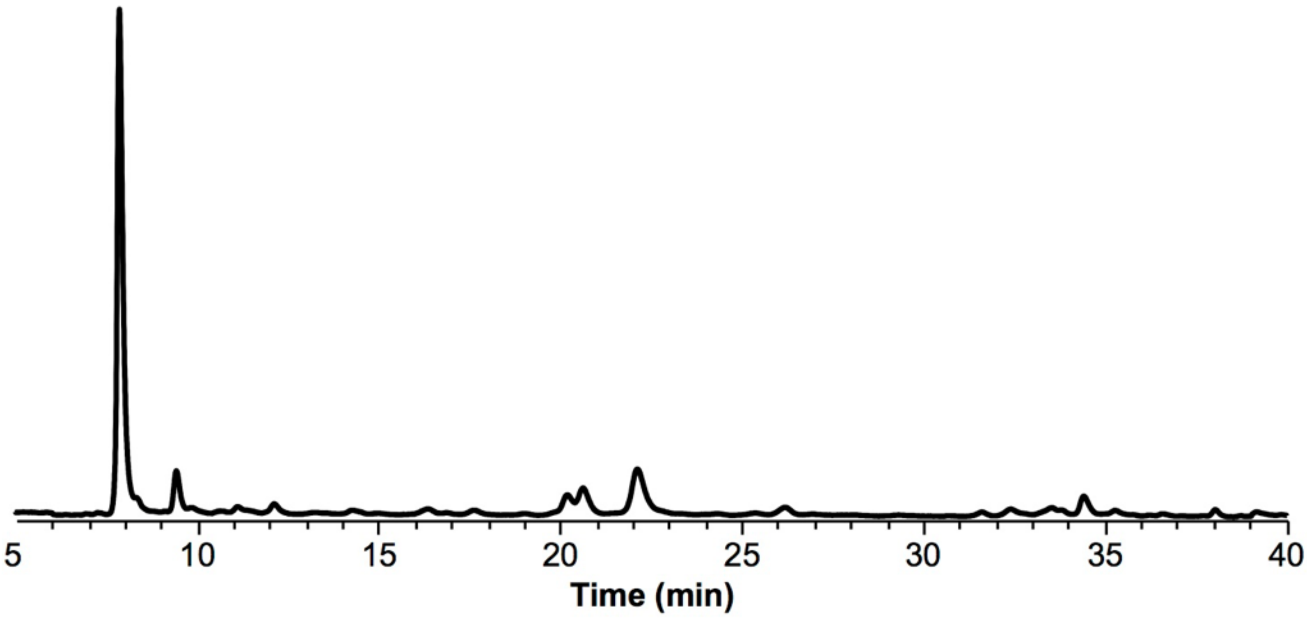

Supplementary Figure 88. Reverse-phase HPLC analysis of a 24 hours old DCL having pure **Cat I RRRR** and DTT as starting building blocks.

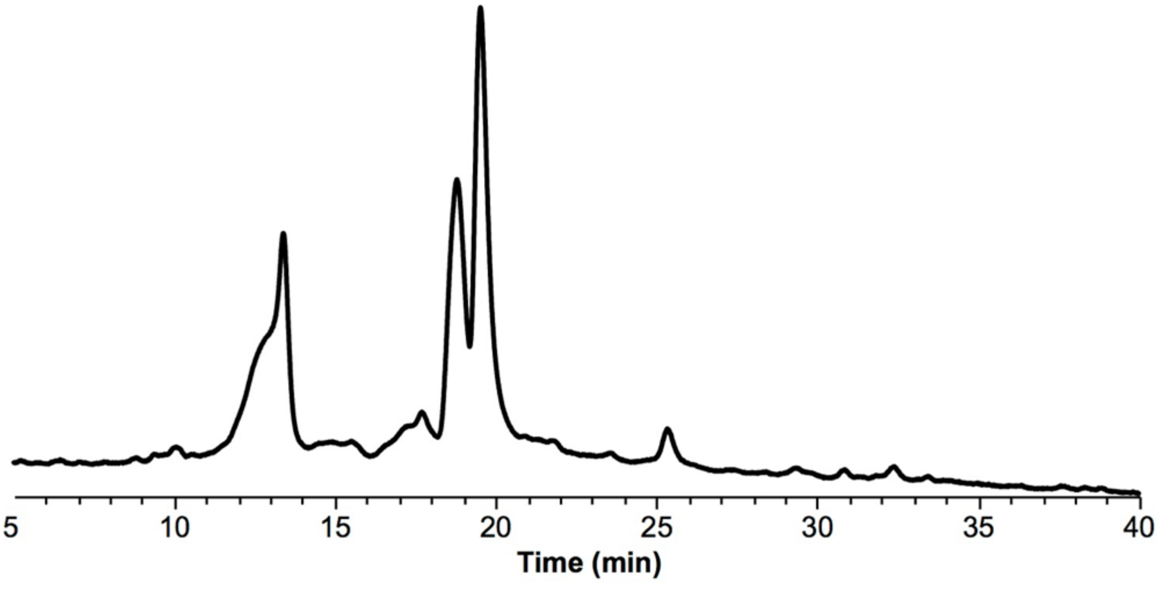

Supplementary Figure 89. Reverse-phase HPLC analysis of a 24 hours old DCL having pure **Cat II RSRR** and DTT as starting building blocks.

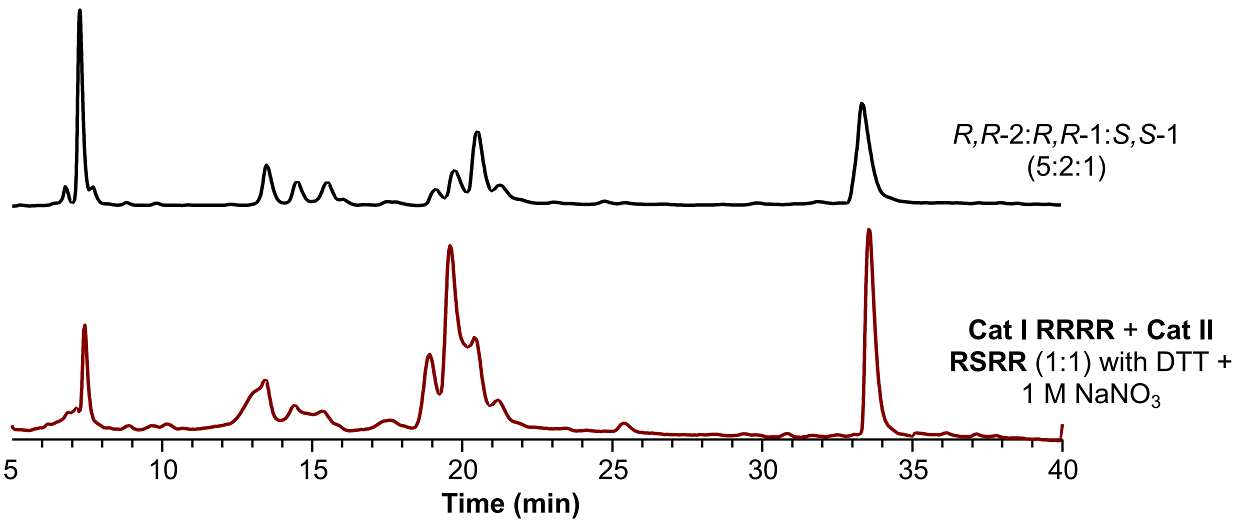

Supplementary Figure 90. Reverse-phase HPLC analysis *R,R*-2:*R,R*-1:*S,S*-1 (5:2:1 molar ratio, 5 mM total concentration) showed in comparison to **Cat I RRRR** and **Cat II RSRR** mixture (1:1 molar concentration) with DTT and 1 M $NaNO_3$.

Computational studies

Geometry optimisation was performed using Avogadro[3] (Force field: UFF, Algorithm: Conjugate gradients). This was followed by MOPAC 2016[4] (Version 18.117M) optimisation PM7 semi-empirical optimisation using the COSMO water model with a 0.1 convergence factor. (MOPAC header: PM7 XYZ EPS=78.39 RSOLV=1.3 CHARGE=0 Singlet BONDS AUX gnorm=0.1).

Supplementary Table 6. Energies of different types of **Cat I RRRR** and **Cat II RSRR**.

Catenane	Energy (kJ mol^{-1})
DAAD **Cat I RRRR**	-8530.72
DAAD **Cat I RSSR**	-8494.23
DAAD **Cat I RSRR**	-8480.76
DADD **Cat II RRRR**	-8501.01
DADD **Cat II RSRR**	-8509.83

DCLSim

DCLs containing the building blocks with and without the NDI-serine competitors were simulated using DCLsim.[5] The results are presented below. For each simulated DCL, we present the building block (and competitor) concentration along with the Gibbs free energy of formation of each library member from its constituent building blocks (ΔG) as well the simulated and observed DCL distribution in percentages. In the DCLs containing competitors, the K_a column refers to the association constant for the competitor to the corresponding library member. We indicated that the competitor does not interact with a library member by adding a "0" in the K_a column. The equilibria responsible for the DCL distribution are presented under each table.

1. DCL of *R,R*-**1** and *R,R*-**2**:

Supplementary Table 7: Concentration of building blocks

Simulated Conditions	Conc (M)
R,R-**2**	0.0026
R,R-**1**	0.0024

Supplementary Table 8: Gibbs free energies of formation of library members, simulated *vs.* observed percentages of library members

Species	ΔG	Sim %	Obs %
**Cat I RRRR**	-33.66	76.71	75.83
**X**	-4.43	15.93	16.38
DDDA (RRRR*)* Macrocycle	-28.51	7.36	7.79

2 *R,R*-**1** + 2 *R,R*-**2** ⇌ **Cat I RRRR** $\Delta G = -RTln\frac{[\mathbf{Cat\ I\ RRRR}]}{[R,R-\mathbf{1}]^2[R,R-\mathbf{2}]^2}$ (1)

R,R-**1** + *R,R*-**2** ⇌ **X** $\Delta G = -RTln\frac{[\mathbf{X}]}{[R,R-\mathbf{1}]^1[R,R-\mathbf{2}]^1}$ (2)

R,R-**1** + 3 *R,R*-**2** ⇌ *DDDA* (RRRR) Macrocycle

$$\Delta G = -RTln\frac{[DDDA\ (\mathrm{RRRR})\mathrm{Macrocycle}]}{[R,R-\mathbf{1}]^1[R,R-\mathbf{2}]^3} \quad (3)$$

2. DCL of *R,R*-**1** and *R,R*-**2** with NDI-*R,R*-serine:

Supplementary Table 9: Concentration of building blocks and of competitor

Simulated Conditions	Conc (M)
R,R-**2**	0.00255
R,R-**1**	0.00245
**NDI-*R,R*-serine**	0.005

Supplementary Table 10: Gibbs free energies of formation of library members, simulated *vs.* observed percentages of library members, their association constant with the competitor and its identification from text

Species	ΔG	K_a (M^{-1})	K_a i.d. in Supplementary Fig. 25	Sim %	Obs %
**Cat I RRRR**	-33.66	0	-	74.80	76.42
**X**	-4.43	81.58	K_{a3}	21.62	19.63
DDDA (RRRR) Macrocycle	-28.51	0	-	3.58	3.95

2 *R,R*-**1** + 2 *R,R*-**2** ⇌ **Cat I RRRR** $\quad \Delta G = -RTln \frac{[\text{Cat I RRRR}]}{[R,R-\mathbf{1}]^2[R,R-\mathbf{2}]^2}$ (4)

R,R-**1** + *R,R*-**2** ⇌ **X** $\quad \Delta G = -RTln \frac{[\mathbf{X}]}{[R,R-\mathbf{1}]^1[R,R-\mathbf{2}]^1}$ (5)

R,R-**1** + 3 *R,R*-**2** ⇌ *DDDA* (RRRR) Macrocycle

$$\Delta G = -RTln \frac{[DDDA\ (\text{RRRR})\ \text{Macrocycle}]}{[R,R-\mathbf{1}]^1[R,R-\mathbf{2}]^3} \quad (6)$$

X + NDI-*R*,*R*-serine ⇌ [X· NDI-*R*,*R*-serine] $\quad K_{a3} = \frac{[\text{X}\cdot \text{NDI}-R,R-\text{serine}]]}{[\mathbf{X}]^1[\text{NDI}-R,R-\text{serine}]^1}$ (7)

3. DCL of *R,R*-**1** and *R,R*-**2** with NDI-*S,S*-serine:

Supplementary Table 11: Concentration of building blocks and of competitor

Simulated Conditions	Conc (M)
R,R-**2**	0.00263
R,R-**1**	0.00237
**NDI-*S,S*-serine**	0.005

Supplementary Table 12: Gibbs free energies of formation of library members, simulated *vs.* observed percentages of library members, their association constant with the competitor and its identification from text

Species	ΔG	K_a (M^{-1})	K_a i.d. in Supplmentary Fig. 25	Sim %	Obs %
**Cat I RRRR**	-33.66	0	-	62.21	62.71
**X**	-4.43	233.11	K_{a2}	28.88	26.25
DDDA (RRRR) Macrocycle	-28.51	0	-	8.91	11.04

2 *R,R*-**1** + 2 *R,R*-**2** ⇌ **Cat I RRRR** $\quad \Delta G = -RTln \frac{[\text{Cat I RRRR}]}{[R,R-\mathbf{1}]^2[R,R-\mathbf{2}]^2}$ (8)

R,R-**1** + *R,R*-**2** ⇌ **X** $\quad \Delta G = -RTln \frac{[\mathbf{X}]}{[R,R-\mathbf{1}]^1[R,R-\mathbf{2}]^1}$ (9)

R,R-**1** + 3 *R,R*-**2** ⇌ *DDDA* (RRRR) Macrocycle

$$\Delta G = -RTln \frac{[DDDA\ (\text{RRRR})\ \text{Macrocycle}]}{[R,R-\mathbf{1}]^1[R,R-\mathbf{2}]^3} \quad (10)$$

X + NDI-*S*,*S*-serine ⇌ [X· NDI-*S*,*S*-serine] $\quad K_{a2} = \frac{[\text{X}\cdot \text{NDI}-S,S-\text{serine}]]}{[\mathbf{X}]^1[\text{NDI}-S,S-\text{serine}]^1}$ (11)

4. DCL of *S,S*-**1** and *R,R*-**2**:

Supplementary Table 13: Concentration of building blocks

Simulated Conditions	Conc (M)
R,R-**2**	0.0025
S,S-**1**	0.0025

Supplementary Table 14: Gibbs free energies of formation of library members, simulated *vs.* observed percentages of library members

Species	ΔG	Sim %	Obs %
**Cat II RSRR**	-32.75	20.89	21.26
**Z**	-3.88	29.65	29.41
**Y**	-3.93	30.03	26.11
DAAA (RSSS) Macrocycle	-32.49	17.55	18.17
DAA (RSS) Macrocycle	0	0.01	2.89
S,S-**1** dimer	2.98	1.87	2.15

S,S-**1** + 3 *R,R*-**2** ⇌ **Cat II RSRR** $$\Delta G = -RTln\frac{[\mathbf{Cat\ II\ RSRR}]}{[S,S-\mathbf{1}]^1[R,R-\mathbf{2}]^3} \quad (12)$$

S,S-**1** + *R,R*-**2** ⇌ **Z** $$\Delta G = -RTln\frac{[\mathbf{Z}]}{[R,R-\mathbf{1}]^1[R,R-\mathbf{2}]^1} \quad (13)$$

2 *R,R*-**2** ⇌ **Y** $$\Delta G = -RTln\frac{[\mathbf{Y}]}{[R,R-\mathbf{2}]^2} \quad (14)$$

3 *S,S*-**1** + *R,R*-**2** ⇌ *DAAA* (RSSS) Macrocycle

$$\Delta G = -RTln\frac{[DAAA\ (\mathrm{RSSS})\ \mathrm{Macrocycle}]}{[S,S-\mathbf{1}]^3[R,R-\mathbf{2}]^1} \quad (15)$$

2 *S,S*-**1** + *R,R*-**2** ⇌ *DAA* (RSS) Macrocycle

$$\Delta G = -RTln\frac{[DAA\ (\mathrm{RSS})\ \mathrm{Macrocycle}]}{[S,S-\mathbf{1}]^2[R,R-\mathbf{2}]^1} \quad (16)$$

2 *S,S*-**1** ⇌ *S,S*-**1** dimer $$\Delta G = -RTln\frac{[S,S-\mathbf{1}\ \mathrm{dimer}]^1}{[S,S-\mathbf{1}]^2} \quad (17)$$

5. DCL of *S,S*-**1** and *R,R*-**2** with NDI-*S,S*-serine:

Supplementary Table 15: Concentration of building blocks and of competitor

Simulated Conditions	Conc (M)
R,R-**2**	0.0025
S.S-**1**	0.0025
NDI-*S,S*-serine	0.005

Supplementary Table 16: Gibbs free energies of formation of library members, simulated *vs.* observed percentages of library members, their association constant with the competitor and its identification from text

Species	ΔG	K_a (M^{-1})	K_a i.d. in Supplementary Fig. 25	Sim %	Obs %
**Cat II RSRR**	-32.75	0	-	16.20	15.91
**Z**	-3.88	84.9	K_{a8}	33.43	30.80
**Y**	-3.93	46.4	K_{a5}	29.30	27.76
DAAA (RSSS) Macrocycle	-32.49	88.4	-	19.58	18.67
DAA (RSS) Macrocycle	0	0	-	0.01	3.87
S,S-**1** dimer	2.98	0	-	1.48	2.97

S,S-**1** + 3 *R,R*-**2** ⇌ **Cat II RSRR** $$\Delta G = -RTln\frac{[\mathbf{Cat\ II\ RSRR}]}{[S,S-\mathbf{1}]^1[R,R-\mathbf{2}]^3} \quad (18)$$

S,S-**1** + *R,R*-**2** ⇌ **Z** $$\Delta G = -RTln\frac{[\mathbf{Z}]}{[R,R-\mathbf{1}]^1[R,R-\mathbf{2}]^1} \quad (19)$$

2 *R,R*-**2** ⇌ **Y** $$\Delta G = -RTln\frac{[\mathbf{Y}]}{[R,R-\mathbf{2}]^2} \quad (20)$$

3 *S,S*-**1** + *R,R*-**2** ⇌ *DAAA* (RSSS) Macrocycle

$$\Delta G = -RTln\frac{[DAAA\ (\mathrm{RSSS})\mathrm{Macrocycle}]}{[S,S-\mathbf{1}]^3[R,R-\mathbf{2}]^1} \quad (21)$$

2 *S,S*-**1** + *R,R*-**2** ⇌ *DAA* (RSS) Macrocycle

$$\Delta G = -RTln\frac{[DAA\ (\mathrm{RSS})\ \mathrm{Macrocycle}]}{[S,S-\mathbf{1}]^2[R,R-\mathbf{2}]^1} \quad (22)$$

2 *S,S*-**1** ⇌ *S,S*-**1** dimer $$\Delta G = -RTln\frac{[S,S-\mathbf{1}\ \mathrm{dimer}]^1}{[S,S-\mathbf{1}]^2} \quad (23)$$

Z + NDI-*S,S*-serine ⇌ [Z · NDI-*S,S*-serine] $$K_{a8} = \frac{[\mathrm{Z}\cdot \mathrm{NDI}-S,S-\mathrm{serine}]}{[\mathbf{Z}]^1[\mathrm{NDI}-S,S-\mathrm{serine}]^1} \quad (24)$$

Y + NDI-*S,S*-serine ⇌ [Y · NDI-*S,S*-serine] $$K_{a5} = \frac{[\mathrm{Y}\cdot \mathrm{NDI}-S,S-\mathrm{serine}]}{[\mathbf{Y}]^1[\mathrm{NDI}-S,S-\mathrm{serine}]^1} \quad (25)$$

DAAA (RSSS) Macrocycle + NDI-*S,S*-serine ⇌ [*DAAA* (RSSS) Macrocycle · NDI-*S,S*-serine]

$$K_a = \frac{[DAAA\ (\mathrm{RSSS})\ \mathrm{Macrocyle}\cdot \mathrm{NDI}-S,S-\mathrm{serine}]}{[\boldsymbol{DAAA}\ (\mathrm{RSSS})\ \mathrm{Macrocyle}]^1[\mathrm{NDI}-S,S-\mathrm{serine}]^1} \quad (26)$$

6. DCL of *S,S*-**1** and *R,R*-**2** with NDI-*R,R*-serine:

Supplementary Table 17: Concentration of building blocks and of competitor

Simulated Conditions	Conc (M)
R,R-**2**	0.0025
S.S-**1**	0.0025
NDI-*R,R*-serine	0.005

Supplementary Table 18: Gibbs free energies of formation of library members, simulated *vs.* observed percentages of library members, their association constant with the competitor and its identification from text

Species	ΔG	K_a (M^{-1})	K_a i.d. in Supplementary Fig. 24	Sim %	Obs %
**Cat II RSRR**	-32.75	0	-	8.37	7.76
**Z**	-3.88	285.3	K_{a9}	31.59	30.28
**Y**	-3.93	297	K_{a6}	32.73	29.24
DAAA (RSSS) Macrocycle	-32.49	566.7	-	26.48	24.76
DAA (RSS) Macrocycle	0	0	-	0.01	5.35
S,S-**1** dimer	2.98	0	-	0.83	2.61

S,S-**1** + 3 *R,R*-**2** ⇌ **Cat II RSRR** $\Delta G = -RT ln \frac{[\textbf{Cat II RSRR}]}{[S,S-\mathbf{1}]^1[R,R-\mathbf{2}]^3}$ (27)

S,S-**1** + *R,R*-**2** ⇌ **Z** $\Delta G = -RT ln \frac{[\mathbf{Z}]}{[R,R-\mathbf{1}]^1[R,R-\mathbf{2}]^1}$ (28)

2 *R,R*-**2** ⇌ **Y** $\Delta G = -RT ln \frac{[\mathbf{Y}]}{[R,R-\mathbf{2}]^2}$ (29)

3 *S,S*-**1** + *R,R*-**2** ⇌ *DAAA* (RSSS) Macrocycle

$$\Delta G = -RT ln \frac{[DAAA\ (\text{RSSS})\ \text{Macrocycle}]}{[S,S-\mathbf{1}]^3[R,R-\mathbf{2}]^1} \quad (30)$$

2 *S,S*-**1** + *R,R*-**2** ⇌ *DAA* (RSS) Macrocycle

$$\Delta G = -RT ln \frac{[DAA\ (\text{RSS})\ \text{Macrocycle}]}{[S,S-\mathbf{1}]^2[R,R-\mathbf{2}]^1} \quad (31)$$

2 *S,S*-**1** ⇌ *S,S*-**1** dimer $\Delta G = -RT ln \frac{[S,S-\mathbf{1}\ \text{dimer}]^1}{[S,S-\mathbf{1}]^2}$ (32)

Z + NDI-*R,R*-serine ⇌ [Z · NDI-*R,R*-serine] $K_{a9} = \frac{[\text{Z} \cdot \text{NDI}-R,R-\text{serine}]}{[\mathbf{Z}]^1[\text{NDI}-R,R-\text{serine}]^1}$ (33)

Y + NDI-*R,R*-serine ⇌ [Y · NDI-*R,R*-serine] $K_{a6} = \frac{[\text{Y} \cdot \text{NDI}-R,R-\text{serine}]}{[\mathbf{Y}]^1[\text{NDI}-R,R-\text{serine}]^1}$ (34)

DAAA (RSSS) Macrocycle + NDI-*R,R*-serine ⇌ [*DAAA* macrocycle · NDI-*R,R*-serine]

$$K_a = \frac{[DAAA\ (\text{RSSS})\ \text{Macrocyle} \cdot \text{NDI}-R,R-\text{serine}]}{[DAAA\ (\text{RSSS})\ \text{Macrocyle}]^1[\text{NDI}-R,R-\text{serine}]^1} \quad (35)$$

a)

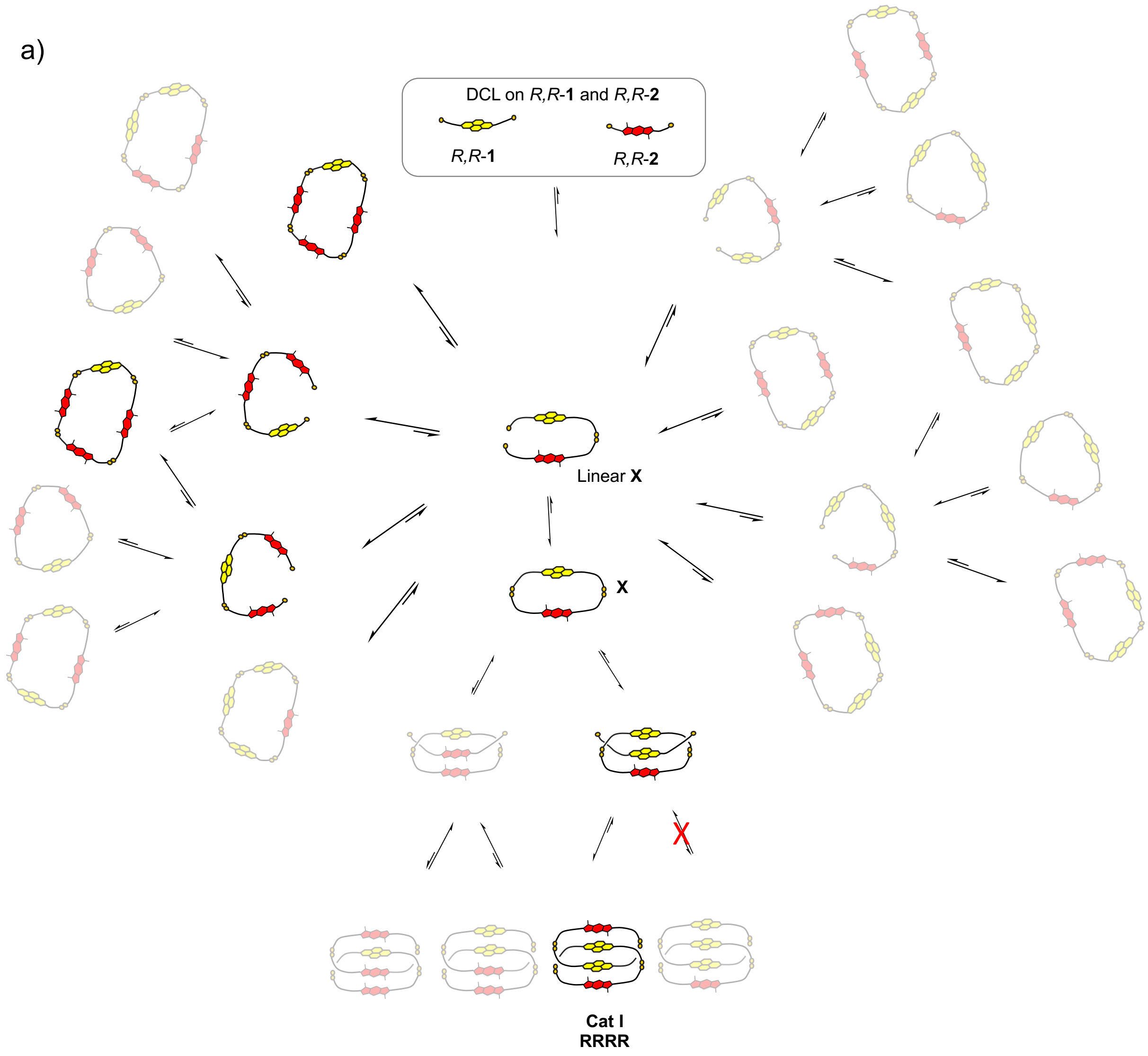

b)

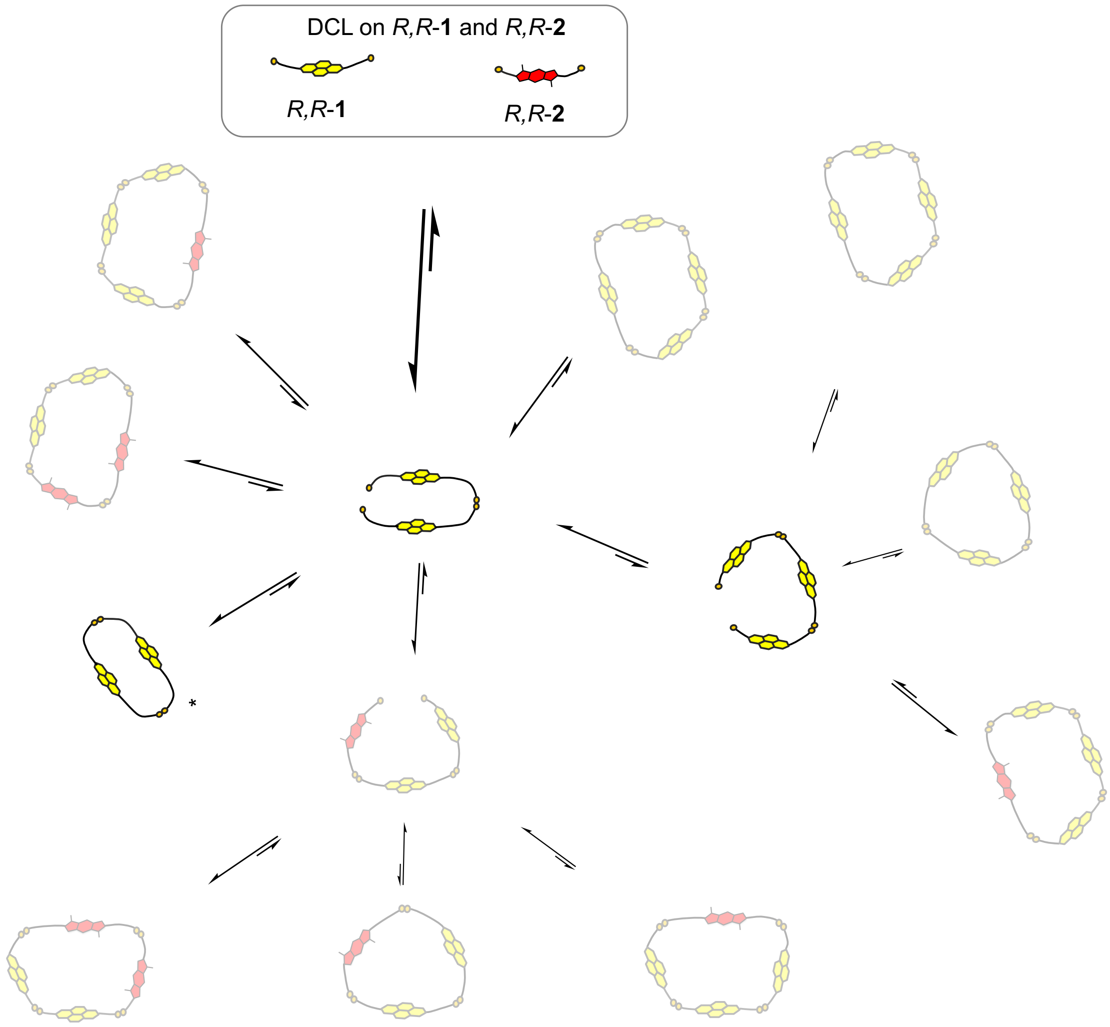

* The cavity is too small to allow any threading

c)

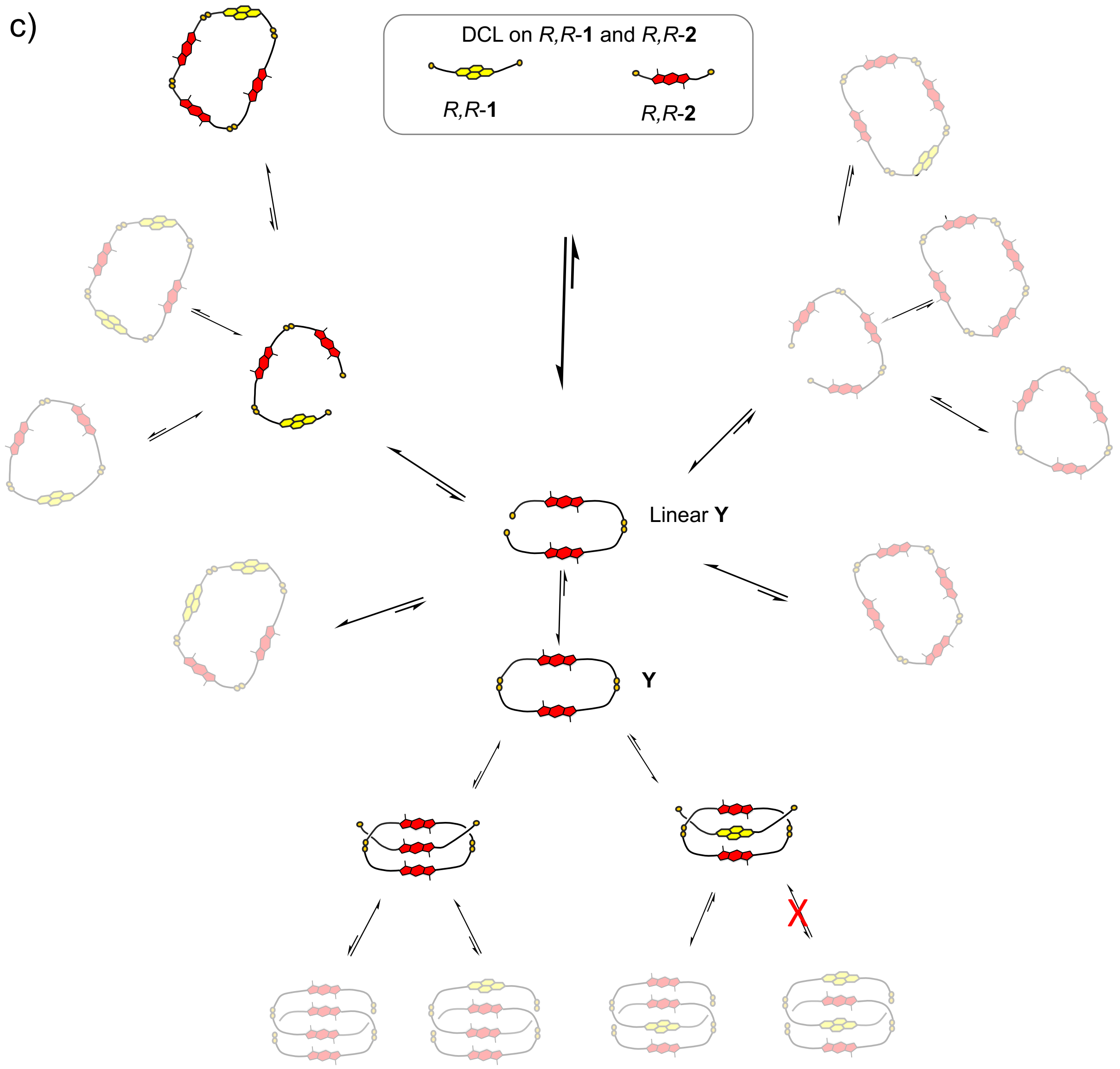

Supplementary Figure 91. The possible equilibria in the library formed between *R,R*-**1** and *R,R*-**2**. Red symbol X suggests the impossibility of formation of the species, whereas the faded species show their less chance of formation based on the library between *R,R*-**1** and *R,R*-**2** (1:1 molar ratio, in the presence of 1 M $NaNO_3$). Due to the size of the figure it has been split in three panels: a), b) and c).

Supplementary References

1. Ota, S. *et al.* Synthesis and properties of a benzo[1,2-b:4,5-b′]dithiophene core π-system that bears alkyl, alkylthio and alkoxy groups at 3,7-positions. *RSC Adv.* **3**, 12356–12365 (2013).
2. Au-Yeung, H. Y., Pantoş, G. D. & Sanders, J. K. M. Dynamic combinatorial synthesis of a catenane based on donor-acceptor interactions in water. *Proc. Natl. Acad. Sci. U. S. A.* **106**, 10466–10470 (2009).
3. Hanwell, M. D. *et al.* Avogadro: an advanced semantic chemical editor, visualization, and analysis platform. *J. Cheminformatics* **4**, 17 (2012).
4. Stewart, J. J. P. Optimization of parameters for semiempirical methods VI: more modifications to the NDDO approximations and re-optimization of parameters. *J. Mol. Model.* **19**, 1–32 (2013).
5. Corbett, P. T., Otto, S. & Sanders, J. K. M. Correlation between Host–Guest Binding and Host Amplification in Simulated Dynamic Combinatorial Libraries. *Chem. - Eur. J.* **10**, 3139–3143 (2004).
